# Liver Dysfunction and Phosphatidylinositol-3-Kinase Signalling in Early Sepsis: Experimental Studies in Rodent Models of Peritonitis

**DOI:** 10.1371/journal.pmed.1001338

**Published:** 2012-11-13

**Authors:** Peter Recknagel, Falk A. Gonnert, Martin Westermann, Sandro Lambeck, Amelie Lupp, Alain Rudiger, Alex Dyson, Jane E. Carré, Andreas Kortgen, Christoph Krafft, Jürgen Popp, Christoph Sponholz, Valentin Fuhrmann, Ingrid Hilger, Ralf A. Claus, Niels C. Riedemann, Reinhard Wetzker, Mervyn Singer, Michael Trauner, Michael Bauer

**Affiliations:** 1 Integrated Research and Treatment Center, Center for Sepsis Control and Care, Jena University Hospital, Jena, Germany; 2 Bloomsbury Institute of Intensive Care Medicine, Division of Medicine, University College London, London, United Kingdom; 3 Electron Microscopy Center, Jena University Hospital, Jena, Germany; 4 Department of Pharmacology and Toxicology, Jena University Hospital, Jena, Germany; 5 Institute of Photonic Technology, Jena, Germany; 6 Division of Gastroenterology and Hepatology, Department of Internal Medicine III, Medical University of Vienna, Vienna, Austria; 7 Institute of Diagnostic and Interventional Radiology, Jena University Hospital, Jena, Germany; 8 Department of Molecular Cell Biology, Center for Molecular Biomedicine, Jena University Hospital, Jena, Germany; Free University of Brussels, Belgium

## Abstract

**Background:**

Hepatic dysfunction and jaundice are traditionally viewed as late features of sepsis and portend poor outcomes. We hypothesized that changes in liver function occur early in the onset of sepsis, yet pass undetected by standard laboratory tests.

**Methods and Findings:**

In a long-term rat model of faecal peritonitis, biotransformation and hepatobiliary transport were impaired, depending on subsequent disease severity, as early as 6 h after peritoneal contamination. Phosphatidylinositol-3-kinase (PI3K) signalling was simultaneously induced at this time point. At 15 h there was hepatocellular accumulation of bilirubin, bile acids, and xenobiotics, with disturbed bile acid conjugation and drug metabolism. Cholestasis was preceded by disruption of the bile acid and organic anion transport machinery at the canalicular pole. Inhibitors of PI3K partially prevented cytokine-induced loss of villi in cultured HepG2 cells. Notably, mice lacking the PI3Kγ gene were protected against cholestasis and impaired bile acid conjugation. This was partially confirmed by an increase in plasma bile acids (e.g., chenodeoxycholic acid [CDCA] and taurodeoxycholic acid [TDCA]) observed in 48 patients on the day severe sepsis was diagnosed; unlike bilirubin (area under the receiver-operating curve: 0.59), these bile acids predicted 28-d mortality with high sensitivity and specificity (area under the receiver-operating curve: CDCA: 0.77; TDCA: 0.72; CDCA+TDCA: 0.87).

**Conclusions:**

Liver dysfunction is an early and commonplace event in the rat model of sepsis studied here; PI3K signalling seems to play a crucial role. All aspects of hepatic biotransformation are affected, with severity relating to subsequent prognosis. Detected changes significantly precede conventional markers and are reflected by early alterations in plasma bile acids. These observations carry important implications for the diagnosis of liver dysfunction and pharmacotherapy in the critically ill. Further clinical work is necessary to extend these concepts into clinical practice.

*Please see later in the article for the Editors' Summary*

## Abstract 2

Experimental studies in a rat model of fecal peritonitis conducted by Michael Bauer and colleagues show that in this model, changes in liver function occur early in the development of sepsis, with potential implications for prognosis and development of new therapeutic approaches.

## Editors' Summary

**Background:**

Sepsis (blood poisoning)—a life-threatening condition caused by an inappropriate immune response to an infection—is a major global cause of death. Normally, when bacteria or other microbes enter the human body, the immune system efficiently destroys the invaders. In sepsis the immune system goes into overdrive, and the chemicals it releases into the blood to combat the infection trigger widespread inflammation (swelling). This leads to the formation of small blood clots and leaky blood vessels that block the flow of blood to vital organs such as the kidneys and liver. In the most severe cases, multiple organs fail and the patient dies. Anyone can get sepsis, but people with weakened immune systems, the very young, and the elderly are most vulnerable. Symptoms of sepsis include fever, chills, rapid breathing, a fast heart rate, and confusion. In its early stages, sepsis can be treated with antibiotics alone, but people with severe sepsis need to be admitted to an intensive care unit where the vital organs can be supported while the infection is treated.

**Why Was This Study Done?:**

Thirty to fifty percent of people who develop severe sepsis die. If sepsis could be diagnosed in its early stages, it might be possible to save more people. Unfortunately, the symptoms of sepsis mimic those of other conditions, and, because sepsis tends to develop very quickly, it is often not diagnosed until it is too late to save the patient's life. The development of liver (hepatic) dysfunction and jaundice are both regarded as late features of sepsis (jaundice is yellowing of the skin and eyes caused by a build-up of bilirubin in the blood). However, the researchers hypothesized that changes in liver function occur early in sepsis and could, therefore, be used to improve the diagnosis and management of sepsis.

**What Did the Researchers Do and Find?:**

The researchers induced sepsis in rats by injecting bacteria into the peritoneal cavity (the gap between the abdominal wall and the abdominal organs), separated the infected animals into predicted survivors and non-survivors based on their heart stroke volume measured using cardiac ultrasound, and then examined their liver function. The expression of genes encoding proteins involved in “biotransformation” and “hepatobiliary transport” (the processes that convert waste products and toxic chemicals into substances that can be conjugated to increase solubility and then excreted) was down-regulated within six hours of sepsis induction in the predicted non-survivors compared to the predicted survivors. Functional changes such as bilirubin and bile acid accumulation in the liver (cholestasis), poor excretion of xenobiotics (molecules not usually found in the body such as antibiotics), and disturbed bile acid conjugation were also seen in predicted non-survivors but not in survivors. Moreover, phosphatidylinositol-3-kinase (PI3K) signaling (which is involved in several immune processes) increased soon after sepsis induction in non-survivor but not in survivor animals. Notably, mice lacking the PI3Kγ gene did not develop cholestasis or show impaired bile acid conjugation after induction of sepsis. Finally, in human patients, plasma bile acids were increased in 48 patients on the day that severe sepsis was diagnosed, and these increases accurately predicted death in these patients.

**What Do These Findings Mean?:**

These findings show that liver dysfunction is an early event in animal models of sepsis and that PI3K signalling plays a crucial role in the development of liver dysfunction. They show that all aspects of liver biotransformation are affected during sepsis and suggest that outcomes are related to the severity of these changes. The limited clinical data included in this study also support the hypothesis that changes in liver function occur early in sepsis, although these data need confirming and extending. Taken together, these findings suggest that liver function tests might aid early diagnosis of sepsis and might also provide information about likely outcomes. They also have important implications for the use of drugs in patients who are critically ill with sepsis, in that some of the drugs routinely administered to such patients may not be adequately detoxified and may, therefore, contribute to organ injury. Finally, these findings suggest that inhibition of PI3Kγ may alleviate sepsis-associated cholestasis.

**Additional Information:**

Please access these websites via the online version of this summary at http://dx.doi.org/10.1371/journal.pmed.1001338.

- This study is further discussed in a *PLOS Medicine* Perspective by John Marshall
- The US National Institute of General Medical Sciences has a fact sheet on sepsis
- The UK National Health Service Choices website has information about sepsis and about jaundice
- The Surviving Sepsis Campaign, which was developed to improve the management, diagnosis, and treatment of sepsis, provides basic information about sepsis
- The Sepsis Alliance, a US not-for-profit organization, also provides information about sepsis for patients and their families, including personal stories about sepsis
- The not-for profit UK Sepsis Trust is another useful source of information about sepsis that includes patient stories
- MedlinePlus provides links to additional resources about sepsis and jaundice (in English and Spanish)

## Introduction

Extrahepatic bacterial infection and the ensuing host inflammatory response (sepsis) account for approximately 20% of cases of jaundice [1]. Hyperbilirubinaemia (bilirubin >34.2 µmol/l), noted in 11% of critically ill patients, is a strong independent risk factor for mortality [2]. This reflects cholestasis and parallels an increase in serum bile acid levels in the late phase of the disease [3]. Laboratory studies support the concept that more subtle alterations occur at the hepatocellular level soon after the onset of sepsis, and that these too may be prognostic [4],[5].

Pathogen-associated molecular patterns trigger release of cytokines and other inflammatory mediators by Kupffer cells, resulting in a local inflammatory milieu [6]. Influx of polymorphonuclear neutrophils, representing a secondary damaging surge, may critically augment the development of excretory dysfunction [7],[8]. These factors impact upon hepatocellular transport proteins, notably the energy-dependent canalicular ATP-binding cassette transporters required for normal bile secretion [9] that underlie sepsis-associated cholestasis [10].

These hepatobiliary transport processes involved in bile formation, also known as phase III biotransformation, frequently require prior processing of hydrophobic or amphiphilic substrates. This is achieved by cytochrome P450–dependent phase I modifications that allow conjugation with various compounds (e.g., glucuronic acid, sulfonates, glutathione, or amino acids) in phase II biotransformation. Apart from their fundamental role in the metabolism of endogenous compounds, these processes are critical determinants of hepatic drug clearance [11]. Thus, altered phase I, II, and III biotransformation may be a critical yet under-appreciated aspect of liver damage in sepsis, where patients often receive >20 different drugs daily [12].

Phosphatidylinositol-3-kinases (PI3Ks) constitute a family of enzymes involved in intracellular signal transduction. The only class IB member, PI3Kγ, has an important role in a number of immune processes and is considered a particularly promising target for adjunctive treatment of (systemic) inflammation [13],[14].

With regard to the physiological regulation of hepatobiliary transport and bile secretion, PI3Ks are essential for intracellular trafficking, selectively regulating ATP-dependent canalicular transporters in both health and disease [15],[16]. Failure of hepatobiliary transport processes results in hepatocellular retention of bile acids, with subsequent triggering of hepatocyte injury due to oxidative stress, apoptosis/necrosis, and mitochondrial toxicity [17]. Although PI3K signalling has received considerable attention in the context of bile acid–mediated apoptosis, its role in hepatic phase I and II metabolism of bile acids has not yet been addressed.

We thus hypothesized that the paradigm of excretory liver dysfunction reflecting a late and uncommon event in sepsis underestimates significant changes in biotransformation that involve PI3K signalling. Using a rat model of polymicrobial sepsis in which echocardiography was used to identify animals likely to die, we herein uncover novel early markers of sepsis-induced liver dysfunction. The systems biology approach applied within this study supports the concept that, in addition to impaired active hepatobiliary transport, key steps of phase I and II metabolism that crucially involve PI3K signalling represent viable therapeutic targets.

## Methods

### Patient Samples

With approval from the ethics committees of the Jena University Hospital and the Medical University of Vienna (2160-11/07), plasma from 48 patients fulfilling standard criteria for severe sepsis/septic shock was sampled on the day of diagnosis and subjected to targeted metabolomic analysis in a prospective, observational manner. Severe sepsis and septic shock were defined according to published criteria [18]. Patients (*n* = 17 at Jena University Hospital, and *n* = 31 at Vienna General Hospital) were eligible for study enrolment if the onset of the syndrome was <24 h prior to study inclusion. Written informed consent was obtained from all patients or their legal representatives. For patients in whom prior consent could not be obtained because of critical illness or the use of sedative or anaesthetic drugs, a surrogate decision maker was fully informed and gave consent. Site investigators used standardised definitions to determine site of infection, the clinical severity scores, and final outcome measures. All patients received sepsis treatment for cardiovascular, respiratory, renal, and metabolic failure according to the guidelines of the German Sepsis Society [19].

### Rat Experiments

All animal procedures were approved by the local authorities (Thuringian State Office for Consumer Protection and Food Safety or the ethics committee of University College London) and were conducted in accordance with UK Home Office guidelines under the 1986 Scientific Procedures Act and with US National Institutes of Health guidelines. Animals were maintained under artificial day–night cycles (12 h light–dark cycles; 23±1°C room temperature, 30%–60% environment humidity), received a standard rat diet and water ad libitum, and were allowed to adapt to laboratory conditions for at least 3 d. All surgical procedures were carried out under aseptic conditions after application of local anaesthesia, while body temperature was maintained at 37°C.

Under a brief period of inhalational anaesthesia, male Wistar rats (12–16 wk old) received tunnelled right jugular vein and left carotid artery catheters that were subsequently mounted onto a swivel-tether system, allowing the rat, on recovery from anaesthesia, to have unimpeded movement in its cage and free access to food and water.

Twenty-four hours later, sepsis was induced by peritoneal contamination and infection with a stool suspension, as previously described [20],[21]. Sham animals of similar age, gender, and weight underwent identical instrumentation and fluid administration, but did not receive faecal slurry.

In previous experiments utilising transthoracic echocardiography, 13 of 14 animals with a 6-h (post-insult) stroke volume <0.17 ml died within 72 h (mortality 93%), compared with one of five animals with a stroke volume ≥0.17 ml (mortality 20%, *p* = 0.006). Hence, a 6-h stroke volume <0.17 ml could prognosticate 3-d mortality with high positive (93%) and negative (80%) predictive values. Consequently, this cutoff value was used in subsequent experiments to distinguish predicted survivors from predicted non-survivors [22]. In these studies, at 6 h after induction of sepsis, liver tissue was taken under terminal anaesthesia for whole genome transcriptomic profiling.

Experiments focussing on protein expression, structural alterations, and functional analysis were performed in both sham-treated animals and animals subjected to lethal sepsis (achieved by increasing the stool inoculum) [21] at 15 h post-insult to allow changes to occur at the functional level and to reduce variability. For determination of cumulative bile flow, groups of animals were instrumented and treated identically, but with additional cannulation of the choledochal duct. Biliary output of glutathione and bile acids was determined as previously described [23]. For non-invasive, whole body near-infrared fluorescence imaging using indocyanine green (ICG), separate groups of animals were instrumented and treated identically but, in addition, were fed with a specific manganese-reduced diet over a 4-wk period prior to instrumentation because of the intrinsic near-infrared properties of manganese. The whole body near-infrared fluorescence imaging device (Maestro, Cri) was equipped with a 710- to 760-nm excitation band pass and an 800-nm emission long pass filter, enabling transcutaneous assessment of whole body ICG fluorescence. Fifteen hours after sepsis induction, native images were taken, followed by administration of ICG (14 pmol/g body weight). Fluorescence intensity was subsequently measured at 0, 30, 150, and 300 min post-injection. Animals were examined at these time points under a brief period of isoflurane anaesthesia and returned to their cages in between. After 300 min, animals underwent laparotomy, and images were taken of the exposed abdominal cavity. The liver was then excised and re-examined by epifluorescence microscopy (AxioObserver Z1, Zeiss), as described previously [24]. Overview images (50× magnification) were generated with the DAPI filter set (365- to 395-nm excitation and 445- to 450-nm emission band pass filters) and ICG filter set (775- to 805-nm excitation and 845- to 855-nm emission band pass filters) using an integrated multichannel mode. At least four perivenous and four periportal areas per animal were further analysed (200× magnification) to assess regional relative pixel intensities.

Hepatic microcirculation and NADH autofluorescence were assessed in vivo 15 h post-insult using the inverted epifluorescence microscope. Video sequences (five regions of interest per animal) were recorded using 20× objective lenses and the DAPI filter set. Sinusoidal perfusion failure was determined by the number of non-perfused sinusoids expressed as a percentage of all sinusoids.

### Mouse Experiments

PI3Kγ^−/−^ (12–16 wk) mice were derived by ten generations of successive backcrosses of heterozygous male knockout mice from chimeric C57BL6/129Sv PI3Kγ^−/−^ mice [25] with C57BL/6 females (Jackson Laboratory). Polymicrobial sepsis was induced by peritoneal contamination and infection, as described previously [26]. Leukocyte–endothelium interactions in the liver were studied 6 h after the septic insult [21]. Briefly, esterase-positive leukocytes were visualised by staining in vivo with 1 mg/kg carboxyfluorescein diacetate succinimidyl ester (Molecular Probes). Esterase-positive leukocytes that migrated into the parenchyma were counted in five regions per animal (10× objective lens) and are indicated as leukocytes per square millimetre of liver surface area.

### Primary and Cultured Hepatocytes

For freeze-fracture replica immunolabelling (FRIL) of multidrug resistance-associated protein 2 (Mrp2), primary hepatocytes were isolated from sham and septic rats by a two-step collagenase perfusion method performed at 15 h. After inserting a portal venous catheter, the liver was immediately flushed with a Ca^2+^-free perfusion medium to remove erythrocytes and plasma proteins. Subsequently, the liver was perfused with a collagenase H (Roche)–containing perfusion medium oxygenated with carbogen (95% O_2_, 5% CO_2_) at 37°C at a flow rate of 40 ml/min (recirculating via backflow through the vena cava), followed by various filtration and washing steps.

HepG2 cells for scanning electron microscopy were cultivated in DMEM/F12 medium supplemented with 10% FCS at 37°C. For either specific inhibition of PI3Kγ or general inhibition of all PI3K subtypes, cells were incubated with AS605240 (Alexis) at an end concentration of 1 µM or wortmannin at an end concentration of 100 nM, respectively. After 1 h, a cytokine mix comprising TNF-α (50 ng/ml), IL-1β (10 ng/ml), IFN-γ (10 ng/ml), and LPS (100 ng/ml; serotype 0111:B4) was added, and cells were incubated for a further 6 h. Untreated cells and cells stimulated with cytokine mix but no inhibitor served as controls.

For FRIL, HepG2 cells were scraped from the culture dish, subsequently centrifuged at 15*g* for 5 min, frozen, and processed as described for Mrp2 labelling of primary hepatocytes.

### RNA Isolation and Genome-Wide Expression Profiling

Liver samples (30–50 mg) were homogenized in 900 µl of lysis buffer from RNeasy Mini Kit (Qiagen) with the Qiagen tissue lyser, and further processed following manufacturer's instructions. RNA was eluted with DEPC water, its concentration determined by UV-spectrophotometry (Nanodrop), and its integrity verified by denaturing RNA gel electrophoresis and analysis on a Bioanalyzer platform (Agilent Technologies). After reverse transcription and amplification of 200 ng of total RNA (Illumina RNA Amplification Kit, Ambion), cRNA was quantified spectrophotometrically, followed by hybridization of 750 ng of cRNA onto an Illumina Sentrix RatRef-12 Expression BeadChip. Each array covers 22,523 rat gene targets based on RefSeq content, with each feature represented on average 30 times. Streptavidin-coupled Cy3 (FluoroLink Cy3, GE Biosciences) was used for staining. Thereafter, arrays were washed, dried, and immediately scanned on an Illumina BeadArray Reader.

### Western Blot Analysis

Similar amounts of protein (50 µg) from microsomal, peroxisomal, or cytosolic fractions were separated by SDS-PAGE, transferred to a PVDF membrane, and probed to bile acid-Coenzyme A: amino acid N-acyltransferase (BAAT) (Santa Cruz Biotechnology). Immune complexes were detected using ECL Western HRP substrate (Millipore). Equal protein loading was confirmed by re-probing the membranes for β-actin (Abcam).

### Immunofluorescence Analysis of Transporter Expression

Frozen rat liver sections were stained for bile salt export pump (Bsep) using rabbit anti-Bsep (1∶100, provided by Bruno Stieger), or double-stained overnight using a mixture of rabbit anti-Mrp2 (1∶500, Sigma) and mouse anti-Na,K-ATPase (1∶200, Abcam). The sections were then washed and subsequently incubated for 2 h with a mixture of Cy3 anti-rabbit IgG (1∶100) and Cy5 anti-mouse IgG (1∶100) (both Jackson ImmunoResearch Laboratories). Negative controls were performed by omitting primary antibodies. Pictures were taken on a confocal laser scanning microscope (LSM510 META, Carl Zeiss). To compare the immunofluorescence intensity of different livers, instrument settings were kept constant across the complete series of experiments.

### Electron Microscopy (FRIL, Scanning Electron Microscopy, Ultrathin Sections)

FRIL was performed using overnight incubation with a rabbit polyclonal anti-MRP2 primary antibody (1∶50) (M8316, Sigma), followed by incubation with a gold-conjugated (10 nm of gold) goat anti-rabbit IgG secondary antibody (1∶50, British Biocell International) for 1 h [27]. Images were taken as digital pictures on an EM902A transmission electron microscope (Carl Zeiss) using a 1k FastScan CCD camera (TVIPS).

For scanning electron microscopy, HepG2 cells grown on glass coverslips were washed in 0.1 M (pH 7.2) sodium cacodylate buffer (SCB), fixed with glutaraldehyde (2.5% in SCB) for 45 min and post-fixed with osmium tetroxide (1% in SCB). Samples were then dehydrated by rising ethanol concentrations in a BAL-TEC CPD 030 Critical Point Dryer (BAL-TEC) and gold sputter coated (layer thickness 20 nm) in a SCD005 Sputter Coater (BAL-TEC). Samples were examined in a LEO 1450VP scanning electron microscope (Carl Zeiss) at 8 kV acceleration voltage and a working distance of 6 mm using a secondary electron detector.

Ultrathin sections were prepared from liver biopsies fixed with glutaraldehyde (2.5% in SCB) and osmium tetroxide (1% in SCB). Samples were dehydrated in rising ethanol concentrations and infiltrated with Araldite resin. Curing of the resin was performed for 48 h at 60°C. Embedded samples were ultrathin-sectioned with a LKB 8800A Ultratome III (LKB Produkter), picked onto Formvar-coated grids and stained with lead citrate for viewing in the transmission electron microscope (see above).

### Laboratory Markers of Inflammation and Sepsis-Associated Liver Injury and Cholestasis

For quantification of IL-6, MCP-1, and TNF-α in mice, a commercially available flow cytometric bead array was used according to the manufacturer's instructions (Mouse Inflammation Kit, BD Biosciences). Plasma samples of rats and mice were analysed for markers of organ dysfunction and injury, including total bilirubin, gamma-glutamyltransferase, and alanine aminotransferase by an automated veterinary clinical chemistry analyser (Fuji Dri-Chem 3500i and Poch-100iv-Diff, Sysmex).

### Determination of ATP Content

ATP was extracted from 10-mg freeze-dried tissue samples by homogenization in nine volumes of ice-cold 0.5 M perchloric acid. After neutralising the supernatant with 3 M potassium phosphate solution, the ATP concentration was determined by a luciferase-based luminometric assay (ATPLite, PerkinElmer) and quantified against a standard curve. The acidic protein pellet was then re-solubilised in 0.1 N NaOH and used for determination of protein content for standardisation of ATP content to tissue protein.

### Phase I and II Model Reactions

Activities of all biotransformation reactions were assessed in 9,000*g* supernatants of liver homogenates in 0.1 M sodium phosphate buffer (pH 7.4) (1∶3 w/v) and referenced to the protein content of this fraction. For assessment of rat CYP1A, CYP2A, CYP2B, CYP2C, and CYP2E activity, ethoxycoumarin O-deethylation was performed, determining the main metabolite 7-hydroxycoumarin fluorometrically [28]. To measure CYP3A activity, ethylmorphine N-demethylation was performed according to the method of Klinger and Müller [29], determining the reaction product (formaldehyde) photometrically. Glutathione-S-transferase activity was determined by performing 1-chloro-2,4-dinitrobenzene conjugation and measuring the resulting dinitrobenzene-glutathione conjugate, GS-DNB, photometrically [30]. Bilirubin glucuronidation was also assessed photometrically, using Burchell's method [31].

### Micro-Raman Spectroscopy

Images were recorded using the Raman spectrometer RXN1 (Kaiser Optical Systems) equipped with a 785-nm diode laser for excitation and a Leica microscope as microprobe. Raman images were recorded in serial mapping mode using a 100×/0.9 objective with a step size of 2.5 µm and an 8-s exposure time per spectrum. HoloGrams software (Kaiser Optical Systems) controlled data acquisition, intensity normalisation, and wave number calibration. Hyperspectral datasets were processed by vertex component analysis. This linear spectral unmixing algorithm estimates the number of pure substances (“end-members”), their spectral signatures, and abundance fractions. The goal was to describe all spectra by a linear combination of these end-members. Details regarding Raman images from liver tissue sections have been previously published [32].

### Targeted Metabolomics

Frozen tissue samples were weighted into 2-ml Precellys tubes equipped with ceramic beads (Peqlab Biotechnologie). A mixture of ethanol/10 mM phosphate buffer 85∶15 (v/v) was then added to the tissue sample in a 1∶3 ratio (w/v). The Percellys tubes and contents were inserted into a homogenizer (Peqlab Biotechnologie) set with a figure-eight motion at 0–4°C, with the program (frequency, cycles, cycle time, pause between cycles) set at 5,800 rpm, 3×30 s, 25 s. After homogenization, the homogenates were centrifuged at 18,000*g* at 2°C for 5 min. The resulting supernatants were pipetted into cryovials (1.5 ml, Biozym) and analysed immediately to avoid degradation. In brief, a mixture of isotopic internal standards and a 20-µl sample volume of plasma derived from either human or rodent sources or tissue supernatant after homogenization were placed together into a 96-well format capture plate. Proteins were precipitated by addition of 200 µl of acetonitrile and centrifugation. Supernatants were transferred onto a new filter plate with 7-mm filter spots, dried down, and hydrolysed with 0.35 M KOH in 95% ethanol. Metabolites were extracted with 100 µl of aqueous methanol. An aliquot of 20 µl of extracted sample was injected into an API4000 QTrap tandem HPLC-MS/MS system (AB Sciex/MDS Analytical Technologies). Acquired analytical data were quantified with Analyst 1.4.2 software (Applied Biosystems) and exported for statistical analysis.

### Statistics

In brief, analysis of microarray data was performed using R software in combination with Bioconductor packages, and included quality control and normalisation [33]. Statistical significance was assessed by false discovery rate–adjusted *p*-values from one-way ANOVA for each bead type [34]. Unsupervised *k*-means clustering was applied to group the bead types with highly similar expression profiles, which were then plotted in a heatmap. Results from *k*-means clustering were based on a minimal intra-class clustering error obtained from 500 iterations for up to *n* = 10 clusters. The transformed log_2_ expression values of each transcript taken from the sham, predicted sepsis survivor, and predicted sepsis non-survivor groups were compared with those of a naïve group, using a cutoff of ≥2-fold down- or up-regulation as significantly regulated. Transcripts regulated <2-fold in all three groups compared to naïve animals were excluded. Additionally, significant bead types were subjected to Ingenuity Pathway Analysis software (Ingenuity Systems), disclosing probable pathophysiologic inter-relationships associated with the expression pattern. The microarray experiment description file according to the MIAME checklist is available on request.

All other data are presented as boxplots illustrating medians within boxes from first quartile (25th percentile) to the third quartile (75th percentile) and whiskers ranging from the 10th to the 90th percentiles (extreme values are marked outside) or are depicted for average sham or wild-type adjusted concentration ratios. Differences between groups were analysed by non-parametric Mann–Whitney *U* test or Kruskal–Wallis test followed by Tukey's or Dunn's post-hoc analysis, as appropriate. A *p*-value<0.05 was considered significant.

## Results

### Rat Sepsis Models

**Figure 1:**
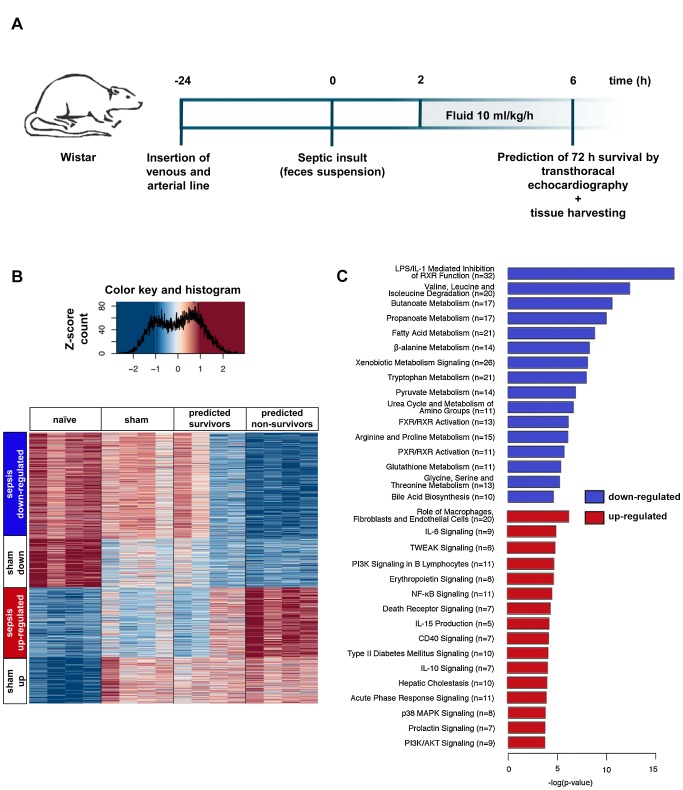
Rat model allowing assessment of early changes in gene expression, and relationships to outcome. (A) Protocol for instrumentation, induction of sepsis, echo-guided prognosis stratification, and tissue harvesting. (B) The heatmap displays expression profiles of 1,373 genes with significantly different expression levels among the naïve, sham, predicted sepsis survivor, and predicted non-survivor groups (*n* = 4 per group). *k*-Means clustering revealed four gene clusters with comparable behaviour. Associated colours represent variance-normalised expression for individual genes: red colour intensity indicates a relative expression greater than the mean, blue indicates the opposite. (C) The transcripts identified in (B) were analysed by Ingenuity Pathway Analysis to identify canonical pathways found to be up- or down-regulated in animals predicted to die. Presented is a bar chart of the most statistically significant (corresponding to *p*-value) down-regulated (blue) and up-regulated (red) pathways associated with poor prognosis. Numbers in parentheses represent the number of differentially regulated transcripts involved in a defined pathway.

As a first step toward identifying molecular mechanisms of incipient liver failure in septic rats, a model of faecal peritonitis utilising echocardiography to predict 3-d mortality as early as 6 h after sepsis insult was applied (Figure 1A). Overall 72-h mortality in this model was 75%. For analysis of the functional implications of the detected transcriptomic changes, a lethal sepsis model was generated using higher volumes of stool suspension (Figure S1).

### Early Changes in the Hepatic Transcriptome Are Associated with Unfavourable Outcomes in Sepsis

Echocardiography-based prognostic stratification enabled identification of early changes in the hepatic transcriptome between predicted survivors and predicted non-survivors. Analysis of gene expression from septic survivors, non-survivors, and naïve and sham-treated animals (*n* = 4 per group) revealed 1,373 significantly altered transcripts (false discovery rate–adjusted *p*-values<0.001) (Figure 1B). These were allocated to four clusters by unsupervised *k*-means cluster analysis. “Sham up” and “sham down” clusters represent transcripts related to anaesthesia and instrumentation. “Sepsis up-regulated” and “sepsis down-regulated” clusters depict changes in transcript abundance that were specific for sepsis, with a significantly more pronounced regulation in either direction in predicted non-survivors. In a cluster-free approach, we compared the average log_2_ fold change values from non-survivors to those from survivors (Figure S2A). Contrast-filtering by 2-fold differences yielded 104 and 184 up- and down-regulated genes, respectively, validating a subset of sepsis-specific candidate genes obtained from clustering with stringent filtering. Transcripts discriminating the predicted outcome were changed in non-survivors by a higher amplitude (linear fit: slope = 1.6).

Next, a detailed search was made for critical pathways that could distinguish survivors from non-survivors. Ingenuity Pathway Analysis revealed a common decrease in metabolic processes related to amino acid and fatty acid metabolism, bile acid and xenobiotic metabolism, and upstream transcriptional regulation by nuclear receptors. These metabolomic processes also discriminated for predicted outcome, including the primarily down-regulated canonical pathway “bile acid biosynthesis” (Figure S2B). With regard to up-regulated processes, non-parenchymal effector functions including cytokine signalling and phosphate transfer (by kinases) were mainly identified. With this approach, the most statistically significant regulated pathways associated with poor prognosis could be identified (Figure 1C). These involved down-regulation of phase I, II, and III metabolism in both comparisons (Figure S3). A full list of up- and down-regulated transcripts in the sepsis-related clusters is shown as Table S1.

### Lethal Sepsis Causes Severe Cholestasis with Hepatocellular Accumulation of Bilirubin, and Failure to Excrete Xenobiotics in the Perivenous Region

Having identified early and potentially dysfunctional signalling cascades, we then characterised their contribution to the development of organ failure in rats subjected to lethal sepsis by extending the duration of sepsis to 15 h to allow development of overt organ dysfunction (5–12 per group). Animals displayed laboratory signs of severe cholestasis at 15 h after sepsis induction, however only minimal increases in alanine aminotransferase and gamma-glutamyltransferase, traditional markers of hepatocellular injury, were observed (Table 1). Serum concentrations of total bilirubin doubled in rats subjected to lethal sepsis compared to sham-treated animals, while cumulative bile flow was reduced to approximately 40% of normal. There was a concomitant reduction of biliary output of bile acids and glutathione, or even absence of the latter. As a reflection of potential mechanisms underlying cholestasis in polymicrobial sepsis, impairments in microvascular perfusion—denoted by a near 3-fold increase in the number of non-perfused sinusoids (21.5% [interquartile range: 15.2–46.5] versus 8.5% [4.5–17.5], *p*<0.001, *n* = 8 per group) and bioenergetic failure, with a significant decrease in ATP content (4.3 nmol ATP/mg protein [3.1–5.6] versus 1.0 [0.7–1.1], *p* = 0.006, *n* = 5 sham, *n* = 8 sepsis)—were observed despite maintained NADH autofluorescence (10,685 arbitrary units [9,667–12,433] versus 11,539 [10,857–25,589], *p* = not significant, *n* = 8 per group).

**Table 1:** Assessment of markers for hepatocellular injury and sepsis-associated cholestasis at 15 h after infection.

Marker	Units	Sham Median (25th; 75th Percentile)	Sepsis Median (25th; 75th Percentile)
Alanine aminotransferase (serum)	U/l	28 (20.5; 39.5)	103.0* (33.0; 119.5)
Gamma-glutamyltransferase (serum)	U/l	4 (1.0; 4.5)	11.5** (8.0; 17.3)
Total bilirubin (serum)	µmol/l	6.8 (5.6; 8.1)	16.3** (12.0; 18.4)
Cumulative bile flow	µl·kg^−1^·min^−1^	61.7 (50.9; 70.3)	22.8** (18.1; 34.4)
Glutathione (biliary)	mol/l	1.9 (1.2; 2.5)	n.d.**
Bile acids (biliary)	mmol/l	29.2 (24.3; 45.8)	21.4* (18.3; 25.4)

Markers were assessed in serum or bile, as specified.

* *p*<0.05 or

** *p*<0.01 compared to sham; *n* = 12 animals for each condition.

n.d., not detectable.

**Figure 2:**
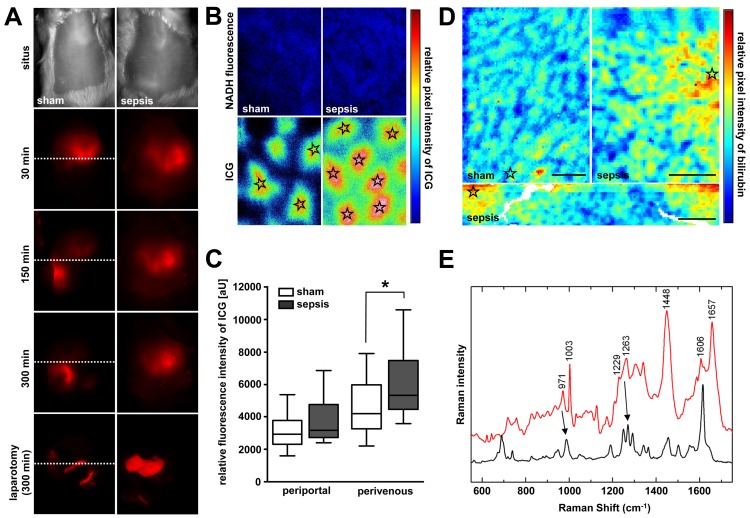
Sepsis-induced excretory dysfunction, as visualised by accumulation of the xenobiotic indocyanine green and bilirubin. (A) Non-invasive whole body near-infrared fluorescence imaging of ICG at 15 h after sepsis induction. Shown are representative images from sham-treated animals (left panels) and animals subjected to lethal sepsis (right panels) for five biological replicates each. While ICG is eliminated via hepatobiliary excretion into the duodenum within the 300-min observation period in sham animals, the dye accumulates in the livers of septic animals, with an almost absence of fluorescence signal in the gut, as confirmed after laparotomy (lower panels). The dotted line divides upper and lower abdominal quadrants for orientation. (B) Subsequent epifluorescence microscopic examination of liver surfaces at 300 min after ICG administration in sham and septic animals. Stars indicate central veins (magnification 50×, pseudo-coloured). (C) ICG fluorescence intensities around central veins were significantly higher in septic compared to sham-operated animals (*n* = 4 animals/group, five perivenous and five periportal areas per animal, *p = 0.031 compared to sham). (D) Representative micro-Raman images of tissue sections from liver sections obtained from sham-operated and septic rats 15 h post-insult. In the livers of the septic rats, relative intensities of the bilirubin component are elevated in the perivenous region (stars). In contrast, only minor local spots of accumulated bilirubin could be verified in livers of control animals. Scale bars: 50 µm. (E) Raman spectrum of the bilirubin component (red trace) with crystalline bilirubin for comparison (black trace). Main spectral contributions of bilirubin are resolved near 971, 1,229, and 1,606 cm^−1^ (arrows at 971 and 1,229 cm^−1^; high peak at 1,606 cm^−1^).

Microvascular perfusion and energetic failure are likely contributors to excretory dysfunction. Impaired blood flow delivery due to sinusoidal shutdown reduces the basolateral availability of ligands, while ATP depletion further accenuates canalicular transport failure. Therefore, excretion of bilirubin, representing clearance of an endogenous molecule, and ICG, reflecting the fate of xenobiotics, was examined at the whole organ, subacinar, and cellular levels to permit localisation of excretory dysfunction. When we applied near-infrared whole body imaging to sham animals, specific ICG fluorescence appeared in the upper right abdominal quadrant (liver) 30 min after dye administration, and shifted towards the lower quadrants within the 5-h observation period. On laparotomy and exposure of the liver, no remaining hepatic fluorescence was detectable. Instead, the dye was entirely present within the duodenum, consistent with the normal excretory route of ICG via bile without further enterohepatic recirculation (Figure 2A). By contrast, septic animals manifested considerable hepatic accumulation of dye, with near-absent fluorescence within the gut. Subsequent inspection of these livers by epifluorescence microscopy revealed a significantly higher amount of ICG in the area surrounding the central veins (Figure 2B and 2C). Endogenous bilirubin was elevated in the perivenous region, assessed using micro-Raman spectroscopy (Figures 2D and 2E). Both bilirubin and ICG are excreted via an Mrp2-dependent mechanism [35]; transcripts encoding Mrp2 were among those mRNAs that showed a strong association with predicted outcome (Figures S3 and S4).

### Polymicrobial Sepsis Impairs Canalicular Bile Acid and Organic Anion Conjugate Transport Machinery

**Figure 3:**
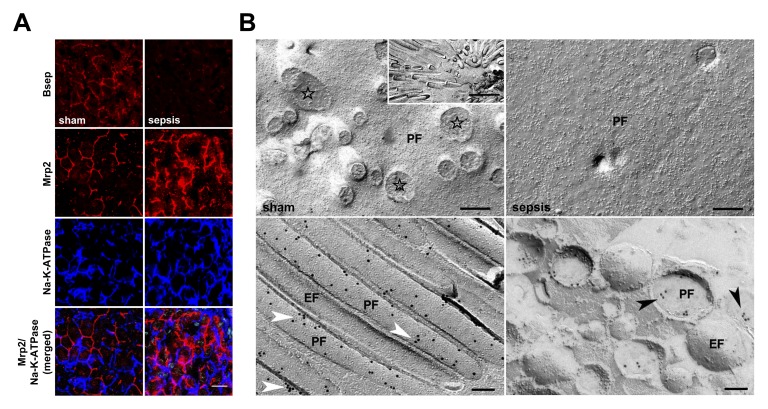
Altered expression of canalicular bile acid and organic anion transporters in sepsis. (A) Distribution and localisation pattern of Bsep and Mrp2 in livers obtained from control and septic rats using confocal microscopy. Na,K-ATPase was used to stain the basolateral plasma membrane. Staining for Bsep revealed a substantial decrease in protein expression after sepsis induction. In sham-treated animals immunoreactive Mrp2 delineates bile canaliculi. The Mrp2 staining pattern in septic rats is irregular, disrupted, and accompanied by recovery of Mrp2 inside the cell, as shown by punctuate staining in the pericanalicular region. Lowermost panels represent merged pictures of Na,K-ATPase and Mrp2. Scale bar: 20 µm. (B) Electron micrographs of freeze-fracture-immunolabelled Mrp2 protein in the plasma membrane of isolated hepatocytes at 15 h post-infection to further study irregular staining observed in sepsis. The cell surface of sham-treated animals is densely covered with microvilli, visible as cross-fractured stubs (stars). A lengthwise fracture of the microvilli membrane exposes a dense labelling with Mrp2 protein arranged in small clusters along microvilli (white arrowheads). By contrast, hepatocytes isolated from septic animals exhibited a loss of microvilli, while Mrp2 was predominantly found in vesicles located closely beneath the canalicular membrane (black arrowheads). The inset shows a top view of a bile canaliculus formed by two adjacent hepatocytes (scale bar: 1 µm). Scale bars in upper and lower panels indicate 200 nm and 100 nm, respectively. EF, exoplasmic fracture face; PF, protoplasmic fracture face.

In view of the association between Mrp2 transcripts and predicted outcome in sepsis, as well as functional data indicating accumulation of bilirubin and ICG, we further characterised the regulation of critical ATP-binding cassette bile acid and organic anion transporters at the canalicular pole. Notably, expression of Bsep at the transcript level remained unchanged in survivors, while steady state concentrations of transcripts for Mrp2 and Bsep were substantially decreased in non-survivors (Figure S4). Immunofluorescence for Bsep within the canalicular membrane was diminished at 15 h in rats subjected to lethal sepsis (Figure 3A). Using confocal laser scanning and electron microscopy, the qualitative distribution and localisation of Mrp2 was assessed in sham and septic rat livers. Figure 3A illustrates the distinct canalicular staining pattern of Mrp2 in control animals, with Mrp2 almost exclusively localised to the canalicular pole of the hepatocytes delineating the bile canaliculi. However, at 15 h after induction of sepsis, this pattern became irregular and disrupted, with fuzzy labelling of the canalicular membrane. Considerable amounts of immunoreactive Mrp2 were recovered inside the cell. These were seen as punctuate staining in the pericanalicular region, consistent with localisation in putative subapical vesicular compartments.

Electron microscopy on primary hepatocytes isolated from sham and septic animals 15 h after sepsis induction confirmed the observed (post-)translational changes, and provided additional information about structural alterations and potential short-term cholestasis-promoting mechanisms. While the cell surface of control hepatocytes was densely covered with microvilli coated with Mrp2 protein, the plasma membranes of hepatocytes from septic rats had almost entirely lost their microvilli (Figure 3B). In these cells, immunoreactive Mrp2 protein was predominantly found in small vesicles of 200–400 nm diameter located closely beneath the canalicular plasma membrane. Quantitative analysis by counting the number of immunogold particles bound to Mrp2 per area coating the plane membrane revealed 8.7 versus 4.9 particles/µm^2^ in hepatocytes recovered from sham and septic animals, respectively. As the microvilli are almost absent in septic animal hepatocytes, only the density of gold particles in microvilli (sham only) and subapical vesicles (sepsis only) was quantified. The lower density of gold particles in septic hepatocytes (130.1 particles/µm^2^ on sham microvilli, 107.2 particles/µm^2^ in septic vesicles) was consistent with an observed 30% decrease of immunoreactive protein assessed by immunoblotting (data not shown).

### Altered Biotransformation of Endo- and Xenobiotics in Sepsis

**Figure 4:**
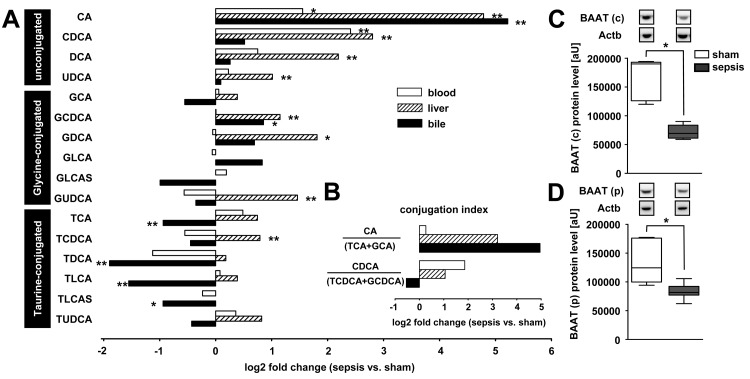
Polymicrobial sepsis causes deranged bile acid conjugation and transport. At 15 h after sepsis induction, plasma, liver tissue, and bile were subjected to targeted metabolomics. Expression of BAAT, facilitating conjugation to taurine and glycine, was quantified by immunoblotting. (A) The plot depicts median log_2_ fold changes of unconjugated as well as glycine- and taurine-conjugated bile acid in plasma, liver, and bile, comparing septic to sham-operated rats (*n* = 12 per group, *p<0.05 or **p<0.01 compared to sham). (B) Conjugation index as a surrogate for the observed conjugation defect reflected by the ratio of unconjugated bile acids CA and CDCA to the corresponding taurine (TCA and taurochenodeoxycholic acid) and glycine (GCA and glycochenodeoxycholic acid) conjugates in plasma, liver and bile (ratio given as log_2_ fold change, *n* = 12 per group). (C and D) Representative immunoblots of BAAT 15 h after sepsis induction in cytosolic (c) as well as peroxisomal (p) fractions, with corresponding densitometric analysis (*n* = 5 for sham, *n* = 8 for sepsis; BAAT (c): *p = 0.002; BAAT (p): *p = 0.006 compared to sham). Densitomentric values are normalised to β-actin. DCA, deoxycholic acid; GDCA, glycodeoxycholic acid; GCDCA, glycochenodeoxycholic acid; GLCA, glycolithocholic acid; GLCAS, glycolithocholic acid sulphate; GUDCA, glycoursodeoxycholic acid; TCDCA, taurochenodeoxycholic acid; TLCA, taurolithocholic acid; TLCAS, taurolithocholic acid sulphate; TUDCA, tauroursodeoxycholic acid; UDCA, ursodeoxycholic acid.

The altered hepatic gene transcripts relating to outcome in the sepsis model included not only transporters but also cytochromes crucial for phase I enzymes related to phase II detoxification (Figures S3 and S4). As observed for active hepatocellular transport, these processes also affect handling of endogenous ligands, notably bile acids, as well as xenobiotics. Animals subjected to lethal sepsis exhibited a bile acid pattern indicative of a profound conjugation defect. The fraction of unconjugated bile acids, particularly cholic acid (CA) and chenodeoxycholic acid (CDCA), was significantly elevated in all three compartments, i.e., blood, hepatic tissue, and bile. Concentrations of glycine- and taurine-conjugated bile acids were increased within the hepatocellular parenchyma, and significantly lower in bile fluid taken from rats subjected to lethal sepsis (Figure 4A). The ratio of unconjugated CA (as substrate) and its primary conjugation products taurocholic acid (TCA) and glycocholic acid (GCA) indicated a >8-fold increase of unconjugated over conjugated bile acids within septic liver parenchyma, and a 32-fold increase in CA within bile (Figure 4B). Likewise, free CDCA was elevated in blood and liver tissue compared to its conjugation products, and reduced within bile. We next assessed steady state protein levels of BAAT as the key enzyme facilitating conjugation of bile acids with either taurine or glycine within the cytosolic and peroxisomal fractions. Fractionation was performed to address the bimodal distribution of BAAT to cytosol and to peroxisomes performing bile acid conjugation after enterohepatic circulation or amidation of de novo synthesised bile acids, respectively [36]. BAAT immunoreactive protein levels were diminished by approximately 60% and 40% within the cytosolic and peroxisomal fractions, respectively, at 15 h after sepsis induction (Figure 4C and 4D).

**Figure 5:**
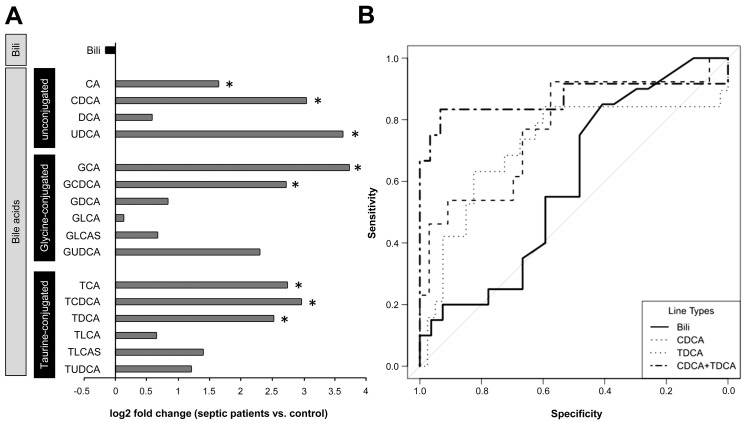
Plasma bilirubin and (un)conjugated bile acid levels in patients on the day of diagnosis of severe sepsis. (A) The plot depicts median log_2_ fold changes of bilirubin (Bili), and unconjugated as well as glycine- and taurine-conjugated bile acid quantities in plasma of severely septic patients (*n* = 48) fulfilling American College of Chest Physicians/Society of Critical Care Medicine consensus criteria compared to non-septic controls (*n* = 20) (*p<0.05 compared to controls). (B) Receiver operating characteristics of bilirubin, CDCA, TDCA, or the combined performance of CDCA+TDCA in predicting 28-d mortality. DCA, deoxycholic acid; GDCA, glycodeoxycholic acid; GCDCA, glycochenodeoxycholic acid; GLCA, glycolithocholic acid; GLCAS, glycolithocholic acid sulphate; GUDCA, glycoursodeoxycholic acid; TCDCA, taurochenodeoxycholic acid; TLCA, taurolithocholic acid; TLCAS, taurolithocholic acid sulphate; TUDCA, tauroursodeoxycholic acid; UDCA, ursodeoxycholic acid.

To explore whether similar deficits in bile acid conjugation occur in septic patients, we obtained plasma from 48 patients fulfilling American College of Chest Physicians/Society of Critical Care Medicine criteria for severe sepsis or septic shock on their day of diagnosis. Clinical characteristics of these patients are summarised in Table 2 and confirm the expected morbidity and mortality (28-d mortality approximately 45%) and the typical sources of sepsis, i.e., primarily pneumonia and abdominal infections followed by blood stream and urinary tract infections. Alterations in unconjugated and conjugated bile acids were consistent with those seen in the rodent model; however, the conjugation deficit remains elusive and requires further investigation, as conjugated bile acids were similarly increased (Figures 5A and S5). In contrast to bilirubin, the increases of CDCA and taurodeoxycholic acid (TDCA) on the day of diagnosis predicted 28-d mortality with high sensitivity and specificity (AUROC: bilirubin: 0.59; CDCA: 0.77; TDCA: 0.72; CDCA+TDCA: 0.87) (Figure 5B).

**Table 2:** Clinical characteristics of septic patients subjected to targeted metabolomic analysis of bile acids.

Characteristic	Units	Value
**Number of patients**	*n*	48
**Age in years**	median (25th; 75th percentile)	64.5 (58.8; 73.5)
**Gender male**	*n* (percent)	30 (62.5)
**28-d survivor**	*n* (percent)	27 (56.3)
**APACHE II**	median (25th; 75th percentile)	28.0 (21.8; 37.3)
**SAPS II**	median (25th; 75th percentile)	59.0 (44.5; 77.0)
**SOFA**	median (25th; 75th percentile)	11.0 (8.0; 14.0)
**Site/Type of infection**		
Pneumonia	*n* (percent)	25 (52.1)
Abdominal infection	*n* (percent)	9 (18.8)
Primary bacteraemia	*n* (percent)	6 (12.5)
Urosepsis	*n* (percent)	5 (10.4)
Other	*n* (percent)	3 (6.25)

APACHE II, Acute Physiology and Chronic Health Evaluation II; SAPS II, Simplified Acute Physiology Score; SOFA, Sequential Organ Failure Assessment.

**Figure 6:**
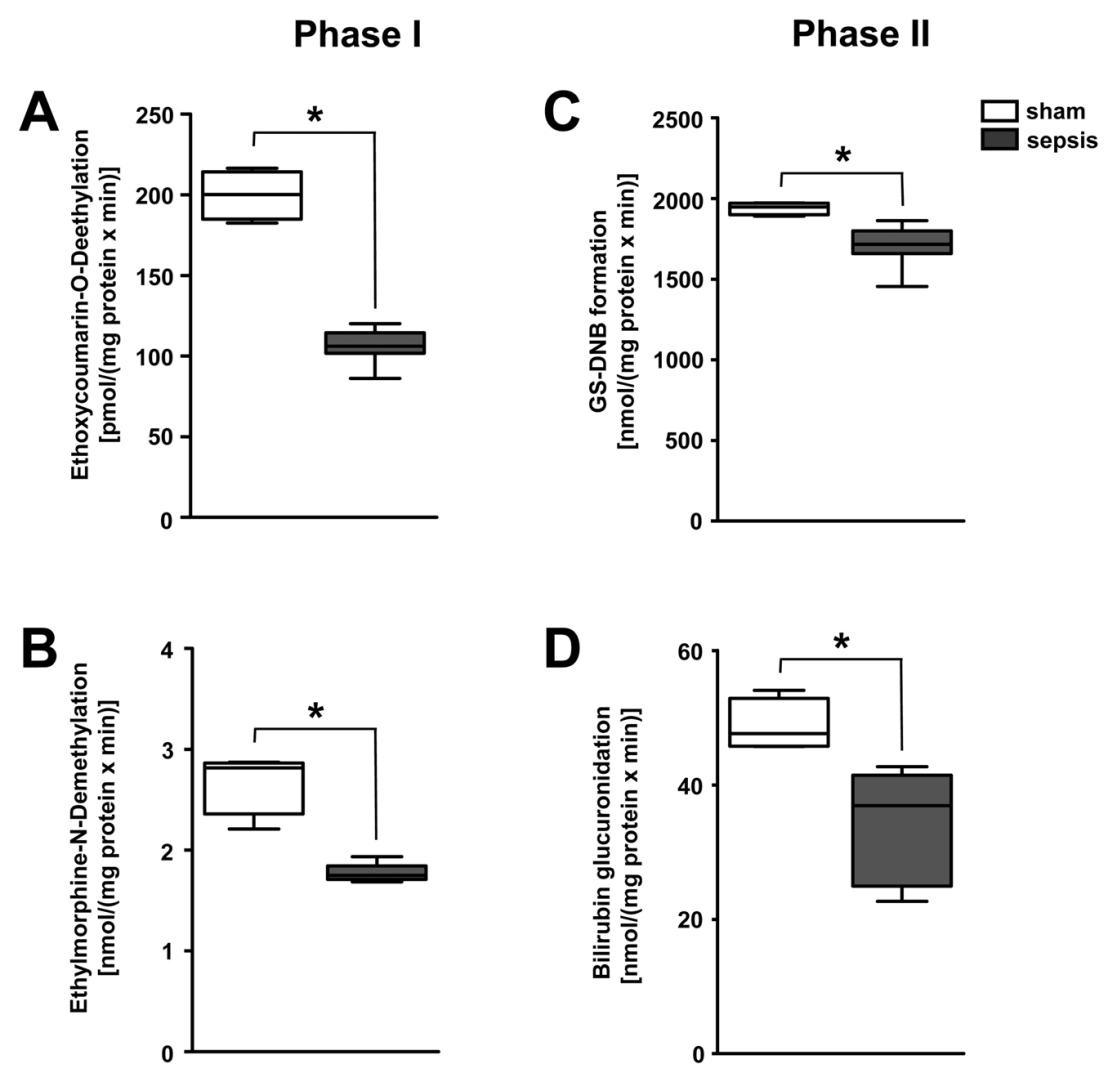
Polymicrobial sepsis severely impairs the activity of enzymes responsible for phase I and II biotransformation. (A) Activities of CYP1A, CYP2A, CYP2B, CYP2C, and CYP2E were assessed by ethoxycoumarin O-deethylation, while (B) CYP3A activity was quantified using the ethylmorphine N-demethylation model reaction for phase I biotransformation. Glutathione-S-transferase activity (C) and bilirubin glucuronidation (D), representing typical phase II conjugation reactions, were assessed by the model reaction 1-chloro-2,4-dinitrobenzene conjugation, resulting in the formation of dinitrobenzene-glutathione conjugate (GS-DNB), or the Burchell method, respectively (*n* = 5 for sham, *n* = 8 for sepsis, *p = 0.004 compared to sham).

To delineate whether impairment of other biotransformation processes important for drug pharmacokinetics exists, typical phase I detoxifying, cytochrome P450–dependent, monooxygenase functions and phase II conjugation activities were analysed in liver homogenates taken from the rodent model (*n* = 5–8 per group). Activities of rat CYP1A, CYP2A, CYP2B, CYP2C, and CYP2E (as assessed by the ethoxycoumarin O-deethylation model reaction) and CYP3A activity (as assessed by ethylmorphine N-demethylation) were significantly impaired in animals subjected to lethal sepsis compared to sham-treated animals (Figure 6A and 6B). Likewise, glutathione-S-transferase activity and bilirubin glucuronidation, representing typical phase II conjugation reactions, were similarly reduced (Figure 6C and 6D).

### Unravelling PI3K as Key Molecular Switch of Sepsis-Associated Cholestasis In Vitro

Since PI3Ks are crucial for the host response and for transporter trafficking, and in view of the association between their (up-)regulation and unfavourable outcomes (Figure 1C), this family of signalling proteins was evaluated further for a mechanistic explanation of sepsis-associated cholestasis.

**Figure 7:**
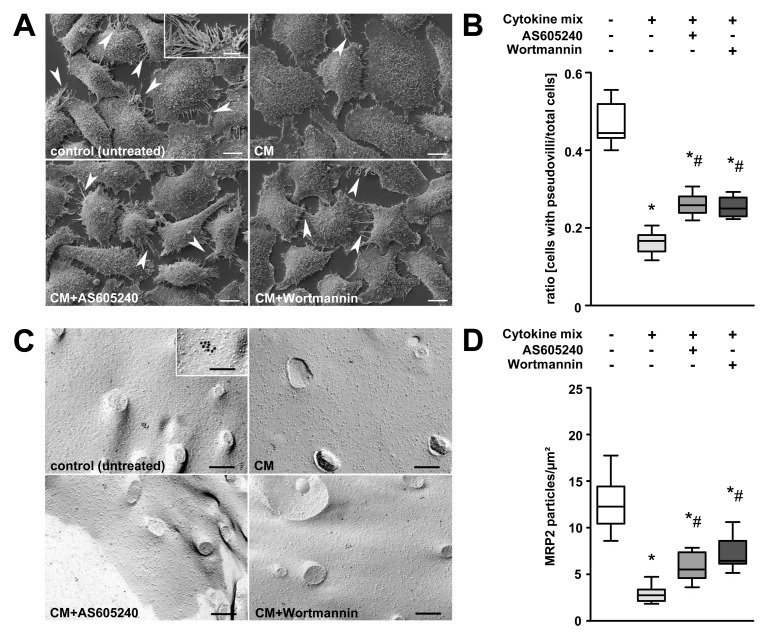
Stimulation of HepG2 cells with prototypical sepsis-associated mediators triggers internalisation of pseudovilli via PI3K signalling. (A) Representative electron microscopic images of HepG2 cells subjected to non-specific (Wortmannin) or specific (AS605240) inhibition of PI3Kγ prior to stimulation with a mix of TNF-α (50 ng/ml), IL-1β (10 ng/ml), IFN-γ (10 ng/ml), and LPS (100 ng/ml) for 6 h. Untreated and cytokine-mix-stimulated cells served as controls. Arrowheads and the inset indicate pseudovilli, representing the equivalent for canalicular microvilli formed by this cell line. Scale bars: 10 µm, inset scale bar: 5 µm. (B) For quantification, the ratio of cells carrying pseudovilli to the total number of cells within the observation field was analysed (*p*<0.001, post-hoc test: *p<0.05 compared to control, ^#^ *p*<0.05 compared to cytokine mix, *n* = 9–13 fields of interest per treatment). (C) Representative electron micrographs of freeze-fracture-immunolabelled Mrp2 transporters in the plasma membrane of HepG2 cells. Cells were treated as described in (A). Inset indicates a cluster of membrane-incorporated immunolabelled Mrp2 protein. Scale bars: 200 nm, inset scale bar: 100 nm. (D) For quantification, Mrp2 particles were assessed and normalised to the protoplasmic fracture face area. (*p*<0.001, post-hoc test: *p<0.05 compared to control, ^#^ *p*<0.05 compared to cytokine mix, *n* = 23–25 fields of interest per treatment).

Two complementary in vitro experiments were performed to characterise the effect of PI3K on insertion/internalisation processes of MRP2/pseudovilli in human hepatoblastoma cells (HepG2) under inflammatory conditions. Treatment of HepG2 cells with a mix of TNF-α, IL-1β, IFN-γ, and LPS induced a retrieval of pseudovilli that represents the equivalent for canalicular microvilli formed by this cell line. While the ratio of cells with pseudovilli to total cells was 0.47 in controls, this dropped to 0.16 in cytokine-mix-stimulated cells (Figure 7A and 7B). Non-specific (Wortmannin) or specific (AS605240) inhibition of PI3Kγ prior to stimulation gave partial protection (Figure 7B).

FRIL of corresponding cells confirmed pseudovilli loss but additionally revealed an 80% decrease of MRP2 protein on the membranes of cells stimulated with cytokine mix. Pre-incubation with AS605240 and Wortmannin significantly suppressed loss of pseudovilli and membrane-bound Mrp2, suggesting a critical role of PI3Kγ in the control of the cytokine-induced internalisation process (Figure 7C and 7D).

### PI3Kγ^−/−^ Mice Are Protected against Hepatic Neutrophil Accumulation and Cholestasis despite an Enhanced Cytokine Response

Given the partial protection of PI3K inhibition against retrieval of pseudovilli in human hepatoblastoma cells, PI3Kγ^−/−^ mice were used to study canalicular microvilli integrity and concomitant development of cholestasis in vivo (*n* = 3–9 per group).

**Figure 8:**
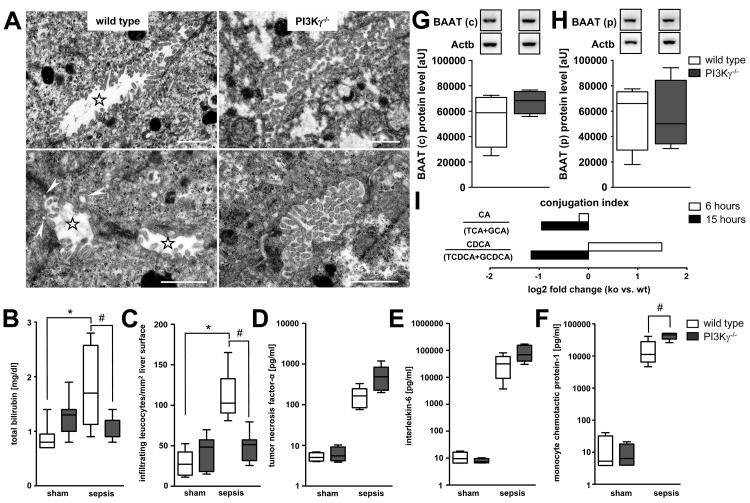
PI3Kγ^−/−^ mice are protected against hepatic neutrophil infiltration and cholestasis despite increased inflammatory mediators. (A) Representative electron micrographs for morphologic assessment of bile canaliculi in liver tissue obtained from wild-type and PI3Kγ^−/−^ mice 6 h after sepsis induction. Stars indicate widened bile canaliculi with disrupted brush borders in wild-type mice. Small vesicles located closely beneath the canalicular plasma membrane were observed in wildtype mice, indicative of subapical vesicles (arrowheads). By contrast, brush borders of PI3Kγ knock-out mice were well maintained. Scale bars: 1 µm. (B) Levels of total bilirubin in sham-operated and septic wild-type and PI3Kγ^−/−^ animals (*p*<0.001, post-hoc test: *p<0.001 wild-type sham versus wild-type sepsis, ^#^ *p* = 0.008 wild-type sepsis versus knock-out sepsis; *n* = 7–9 per group). (C) Assessment by intravital microscopy of infiltrating esterase-positive leucocytes per square millimetre of liver surface in wild-type and PI3Kγ^−/−^ mice at 6 h under sham or septic conditions (*p*<0.001, post-hoc test: *^,#^ *p*<0.05 wild-type sham versus wild-type sepsis, as well as wild-type sepsis versus knock-out sepsis; *n* = 3 animals per group with five regions of interest per animal). (D–F) Mean levels of TNF-α, IL-6, and MCP-1 from wild-type and PI3Kγ-deficient mice at 6 h under sham and septic conditions (*p*<0.001, post-hoc test: ^#^ *p*<0.001 for wild-type sham versus wild-type sepsis, knock-out sham versus knock-out sepsis and wild-type sepsis versus knock-out sepsis; *n* = 7–9 per group). (G and H) Representative immunoblots of BAAT 6 h after sepsis induction in cytosolic (c) and peroxisomal (p) fractions, with corresponding densitometric analysis (*n* = 4 each for wild-type and PI3Kγ^−/−^ samples). Densitomentric values are normalised to β-actin. (I) Conjugation index reflected by the ratio of unconjugated bile acids CA and CDCA to the corresponding taurine (TCA and taurochenodeoxycholic acid [TCDCA]) and glycine (GCA and glycochenodeoxycholic acid [GCDCA]) conjugates in plasma of wild-type and PI3Kγ^−/−^ mice at 6 and 15 h (ratio given as log_2_ fold change, *n* = 5–7 animals/group).

At 6 h after sepsis induction, morphologic examination using transmission electron microscopy revealed disrupted brush borders with widened bile canaliculi in wild-type mice (Figure 8A). Small subapical vesicles located beneath the canalicular plasma membrane were observed in these animals. By contrast, brush borders in septic PI3Kγ^−/−^ mice were well maintained (Figure 8A), and this was paralleled by an absence of hyperbilirubinaemia (Figure 8B).

Overall, in vivo protection in the PI3Kγ^−/−^ mice was more pronounced than the partial protection seen in cultured HepG2 cells. This is consistent with a contribution of cells other than hepatocytes towards mediation of in vivo excretory dysfunction.

As neutrophils may be critical effectors of cholestasis [8],[37], we analysed neutrophil recruitment in this model using in vivo microscopy. This facet of acute inflammation was specifically inhibited in PI3Kγ^−/−^ mice (Figure 8C). Absence of hyperbilirubinaemia and the inability of neutrophils to migrate into liver tissue in the septic PI3Kγ^−/−^ mice were seen, notwithstanding higher levels of inflammatory cytokines (TNF-α and IL-6) and the chemoattractant MCP-1 (Figure 8D–8F).

As our results supported the concept that loss of the transport machinery reflects only one facet of sepsis-associated cholestasis, and as an inflammation-mediated down-regulation of BAAT was identified as the mechanism for impaired bile acid conjugation, we further assessed BAAT and bile conjugation in wild-type and PI3Kγ^−/−^ mice.

Compared to septic wild types, BAAT expression was maintained in the peroxisomal fraction and increased in the cytosolic fraction in PI3Kγ^−/−^ mice 6 h after sepsis induction (Figure 8G and 8H). Because of plasma levels of unconjugated CA and CDCA, PI3Kγ^−/−^ mice maintained the ability to conjugate bile acids. This was most pronounced at 15 h, i.e., a time by which organ failure was fully developed (Figure 8I).

## Discussion

Jaundice is a clinical hallmark associated with a poor prognosis in sepsis. However, as its development does not usually become apparent in the clinical context until days after the onset of disease, liver failure is traditionally considered a late manifestation of sepsis-induced multiple organ failure. The experimental data presented herein provide strong evidence that liver dysfunction affecting both biotransformation and transport of endo- and xenobiotics is an early and frequent event in sepsis, with subclinical alterations occurring within hours of infection. Moreover, we reveal PI3K as a key molecular switch for this series of cellular events in sepsis-associated liver dysfunction.

We used clinically pertinent animal models of faecal peritonitis, in which stroke volume measured 6 h post-sepsis could reliably distinguish between eventual survivors and non-survivors with high sensitivity and specificity [20]. This model is particularly valuable since tissues are harvested at a time point reflecting early (mal)adaptive changes, and when clinical severity is relatively mild and still indistinguishable between survivors and non-survivors.

Genome-wide gene expression analysis combined with a structured network knowledge-based approach enabled us to study early changes in steady state transcript levels that were associated with eventual death. Mediators involved in inflammatory signalling showed a marked increase, principally indicating an enhanced pro-inflammatory, “acute phase” signal derived from both parenchymal and non-parenchymal cells. Simultaneously, down-regulation of housekeeping metabolic functions affecting primarily phase I and II metabolism and molecular transport/phase III metabolism, but also of negative acute-phase reactants, was observed. Of note, the intensity of regulation of gene expression (in either direction) related to disease severity, with a likelihood of death within 72 h being detectable as early as 6 h post-insult. The consistent pattern differentiating survivors from non-survivors points towards a maladaptive response in animals that proceed to die, with transcript levels in eventual survivors being similar to those in sham-treated animals. A similar association of impaired biotransformation with outcome was observed in mice with pneumococcal disease if pneumonia progressed to sepsis [38]. In line with these findings, our present clinical data are consistent with the concept that cholestasis reflects a more generalised response to life-threatening infection and is not restricted to peritonitis, as a similar accumulation pattern of bile acids was observed in the largest subset of included patients presenting with pneumonia.

The orchestrated reprogramming of the transcriptome to infection implies a differential regulation of transcription factors. Coordinated repression of critical nuclear receptors (FXR, RARα, CAR, and PXR) is seen during the acute-phase response (Figure S3). This tallies with prior evidence from endotoxin challenge models [39],[40]. These data confirm and extend previous studies that identified pathways altered by sepsis [41], and additionally determine those transcriptomic changes related to outcome.

To explore the functional implications and underlying cellular mechanisms of cholestasis and altered biotransformation in the animal model, we studied phase I and II biotransformation and transport at 15 h post-insult. Use of this time point enabled transcriptional changes to be manifest at both protein and functional levels. A substantial degree of excretory dysfunction was noted, despite only mild-to-moderate hepatocellular injury consistent with clinical manifestations seen in the critically ill [10],[42]. A substantial increase in plasma bile acids in early sepsis, which was also consistently seen in the patient samples, prompted us to examine changes in the transporter machinery.

The canalicular conjugate export pump Mrp2 is critically involved in the functional measures of cholestasis [43] including excretion of bilirubin and glutathione and its conjugates, and the disposition of divalent bile acids. Our data indicate a substantial functional impairment of this transporter, as reported in rodent endotoxemic models [44],[45]. We noted a severity-related decrease in mRNA and protein for Bsep, the rate-limiting transporter for bile acids, whereas Mrp2 showed a decrease in transcripts in prognosticated non-survivors (Figure S4). However, at 15 h, there was only a moderate decrease in the membrane fraction of Mrp2 protein. In parallel, sizable amounts of Mrp2 were localised within cells close to the canalicular pole. This trafficking implies mobilisation predominantly of canalicular transporters that reside within subapical endosomal pools, but also a reverse pathway resulting in retrieval of transporters from the canalicular membrane due to stimuli such as endotoxin, cytokines, or oxidative stress [46].

Simultaneous withdrawal of microvilli and related transporters from the canalicular membrane suggests substantial alterations to the cytoskeleton. This could include disruption of microtubules and derangement of actin microfilaments in the pericanalicular region, with subsequent loss of microvilli and diminished contractility of the canalicular membrane [47]. These changes are consistent with a PI3K-mediated effect. With the data indicating up-regulation of this critical cellular regulator, we were prompted to study the functional role of PI3K in mediating post-translational alterations in biliary transport. We used ICG as a xenobiotic model substance to gain functional insights into excretory dysfunction. Typically, transcellular ICG transport is microtubule-dependent. Decreased binding of ICG to ligandin(s) due to intracellular accumulation of bilirubin glucuronides may contribute to the impaired ICG excretion seen in Mrp2-deficient hyperbilirubinaemic rats [48]. The distribution and storage of ICG into organelles (“deep compartments”) seen in these rats is consistent with the observed ICG accumulation seen in the septic rats in our study. Failure of canalicular excretion is reflected in the accumulation of both the dye and endogenous bilirubin around the central veins (Figure 2B and 2C), and consistent with the fact that inflammation-induced down-regulation of canalicular transporters, especially Mrp2, is most pronounced in pericentral hepatocytes [49]. Excretory dysfunction may pave the way for accumulation of xenobiotics requiring metabolism in pericentral hepatocytes, i.e., the region prone to injury in the critically ill.

Drugs routinely administered to critically ill patients have poorly understood pharmacokinetics, and concerns regarding their safety and toxicity are increasing [50]. Altered xenobiotic metabolism may further contribute to organ injury. For example, single-dose administration of atorvastatin to septic patients resulted in supra-therapeutic (up to 100-fold) plasma concentrations for up to 20 h [51]. Apart from impaired hepatobiliary transport, a defect in phase I detoxification machinery, including the cytochrome P450 enzymes predominantly involved in xenobiotic biotransformation (i.e., P450 family CYP1, CYP2, and CYP3) and phase II conjugating enzymes (glutathione-S-transferases, bilirubin-UDP-glucuronyltransferase), would result in non-detoxification of substances that are normally bile-excreted. In a cohort of septic children, antipyrine clearance, measuring the detoxifying properties of several CYP enzymes, was reduced by 70% [52].

Impaired phase I and II metabolism was similarly present in septic rats when we analysed biotransformation of bile acids. Septic animals exhibited a profound defect in bile acid conjugation. This key result can be explained by reduced protein levels of BAAT, which facilitates bile acid conjugation. Lack of side chain conjugation may have profound impacts on bile acid distribution (e.g., through cholehepatic shunting within the liver, further contributing to elevated serum bile acid levels) and signalling (e.g., through altered affinity to dedicated G protein–coupled and nuclear receptors) [53]. The present results as well as other recent data [3] indicate bile acid accumulation on the day of diagnosis of sepsis in the absence of hyperbilirubinaemia, and a persistent pattern of increase in bile acids and bilirubin, in those patients who proceed to die [3]. Unlike bilirubin levels, the observed early increase in bile acids predicted 28-d outcome.

Our studies reveal PI3K as a key molecular switch for several major cellular events in sepsis-associated excretory liver dysfunction. Sepsis-induced cholestasis crucially involves neutrophil migration via P-selectin-mediated recruitment [8],[37]. Migration into liver tissue was completely prevented in PI3Kγ^−/−^ mice. Neutrophil and macrophage chemotaxis is mediated by a wide variety of PI3Kγ-dependent factors, and PI3Kγ is also involved in selectin-mediated capture and rolling, integrin-mediated adhesion, and leukocyte extravasation [14]. Thus, PI3Kγ represents a promising target to alleviate sepsis-associated cholestasis [16],[54],[55].

Both hydrophobic and hydrophilic bile acids can activate PI3K. Effects range from cell survival to cell death, a phenomenon termed the “PI3K paradox” [54]. While glycochenodeoxycholic acid and taurolithocholic acid represent the main hydrophobic bile acids exhibiting potent necrotic/apoptotic and cholestatic effects, most hydrophilic bile acids (e.g., TCA and tauroursodeoxycholic acid) promote cell survival, enhancement of bile flow, and restoration of liver function in cholestasis [56]. A deficit in the conjugation process, as demonstrated in the present study, results in further accumulation and potential cytotoxicity of unconjugated bile acids [57]–[59]. In contrast to septic wild-type mice, PI3Kγ^−/−^ mice maintained the ability to conjugate bile acids, implying an additional influence of PI3Kγ on phase I and II bile acid metabolism. Despite higher levels of circulating cytokines, BAAT expression in hepatocytes was preserved or increased in PI3Kγ^−/−^ mice 6 h post-sepsis compared to wild-type septic controls, suggesting that BAAT may be a downstream target of PI3Kγ signalling.

Taken together, our findings challenge the current clinical paradigm that cholestasis reflects an infrequent and late event in sepsis. Strong evidence from the provided animal data support the concept that impaired biotransformation of bile acids (the most prominent endogenous substrates for phase I, II, and III biotransformation machinery) is a consistent and reproducible finding that develops within hours after the onset of sepsis, predicts adverse outcome, and may even represent a therapeutic target. These changes critically depend upon PI3K activity as a molecular switch. Diagnostic tests with superior sensitivity and specificity to reflect altered biotransformation and hepatobiliary transport, as well as novel biomarkers to identify disturbed pharmacokinetics in the critically ill, are required. On a long-term basis, PI3K signalling in sepsis-associated liver dysfunction may offer a promising therapeutic target.

## Supporting Information

Figure S1 **Kaplan-Meier survival analysis of both applied rat sepsis models.** (A) Depicted are survival curves for the lethal sepsis model (black line; *n* = 14), for the survivable sepsis model (grey line; *n* = 19), and for sham-treated animals (dotted line; *n* = 14). Log-rank statistic indicates a significant difference between all survival curves (*p*<0.001). (B) In this survival analysis only the non-survivors (*n* = 14) in the prognosis-stratified sepsis model are included. Log-rank statistic indicates no significant difference between the survival curves of both sepsis models.
(TIF)

Figure S2 **Scatterplot and top ten enriched canonical pathways for the average disparity of transcripts in predicted non-survivors as opposed to predicted survivors.** (A) Average log_2_ fold change for all 11,988 detected transcripts. Red dots: up-regulated more than 2-fold in predicted non-survivors compared to predicted survivors (*n* = 104). Blue dots: down-regulated more than 2-fold in predicted non-survivors compared to predicted survivors (*n* = 184). The linear fits for all log_2_ fold change values exhibit higher amplitudes of expression changes for predicted non-survivors (slope = 1.633). (B) Biplot of top enriched results from Ingenuity Pathway Analysis for the input set of corresponding transcripts (coloured as in [A]). False discovery rate–adjusted *p*-values after Benjamini-Hochberg (B–H, right axis) were computed using Fisher's exact test accounting for the number of hits, the number of uniquely mapped gene IDs (*n* = 260), and the total number of entities in the defined pathway (indicated on top of each bar).
(TIF)

Figure S3 **Ingenuity Pathway Analysis of cellular events discriminating predicted non-survivors from predicted survivors.** The canonical pathway of biotransformation generated with Ingenuity Pathway Analysis software using data from the clusters “sepsis up-regulated” and “sepsis down-regulated” in the heatmap depicts altered transcripts and presumed interactions of the encoded proteins. Coloured molecules (red = up-regulated; green = down-regulated, red/green = differential regulation of isoforms) represent regulated transcripts. While genes involved in inflammatory signalling (i.e., interleukin-1 receptor [IL-1R] and TNF receptor-associated factor 2 [Traf2]) were up-regulated, the majority of genes participating in cellular metabolic functions showed steady state transcript levels that were substantially decreased in prognosticated septic non-survivors as compared to predicted survivors. Transcript levels encoding critical nuclear receptors, such as farnesoid X receptor (FXR), retinoic acid receptor-α (RARα), constitutive androstane receptor (CAR), and pregnane X receptor (PXR), were coordinately down-regulated. Genes involved in downstream processes responsible for phase I/II metabolism (i.e., cytochrome P450 [CYPs], sulfotransferases [SULTs], and glutathione-S-transferases [GSTs]) and phase III transport (Mrp2, Na^+^-taurocholate cotransporting polypeptide [Ntcp], and organic anion transporting polypeptides [Oatps]) of endobiotic as well as xenobiotic compounds were affected, with a decreased gene expression associated with poor prognosis.
(TIF)

Figure S4 **Disease severity–dependent changes of transcripts involved in phase I/II bile acid metabolism and transport.** The figure depicts log_2_ transformed fluorescence units of critical transcripts encoding proteins involved in bile acid biosynthesis and transport—including CYP7a1 (cholesterol-7-alpha-hydroxylase), CYP27a1 (sterol 27-hydroxylase), BAAT, Ntcp (Na^+^-taurocholate cotransporting polypeptide), Bsep (bile salt export pump), and Mrp2—that were regulated according to disease severity.
(TIF)

Figure S5 **Raw data of bilirubin and unconjugated, glycine-, and taurine-conjugated bile acids stratified according to 28-d outcome.** Bilirubin levels are compared to reference control value (21 µmol/l). The number of detected analytes is given below each boxplot. Significance of differences for survivors versus control (*p<0.05, **p<0.01) and non-survivors versus control (^#^ *p*<0.05; ^##^ *p*<0.01) is provided below the analyte symbol.
(TIF)

Table S1 **Full list of up- and down-regulated transcripts out of cluster 1 and 3 for the comparison of predicted non-survivors with predicted survivors.**
(DOC)

## Abbreviations

AUROC: area under the receiver-operating curve
BAAT: bile acid-Coenzyme A: amino acid N-acyltransferase
Bsep: bile salt export pump
CA: cholic acid
CDCA: chenodeoxycholic acid
FRIL: freeze-fracture replica immunolabelling
GCA: glycocholic acid
ICG: indocyanine green
Mrp2: multidrug resistance-associated protein 2
PI3K: phosphatidylinositol-3-kinase
SCB: sodium cacodylate buffer
TCA: taurocholic acid
TDCA: taurodeoxycholic acid
