# Supplementary figures and images for: Liver Dysfunction and Phosphatidylinositol-3-Kinase Signalling in Early Sepsis: Experimental Studies in Rodent Models of Peritonitis
Source: PLoS Med. 2012 Nov 13;9(11):e1001338. doi: 10.1371/journal.pmed.1001338 (PMC3496669; doi:10.1371/journal.pmed.1001338)

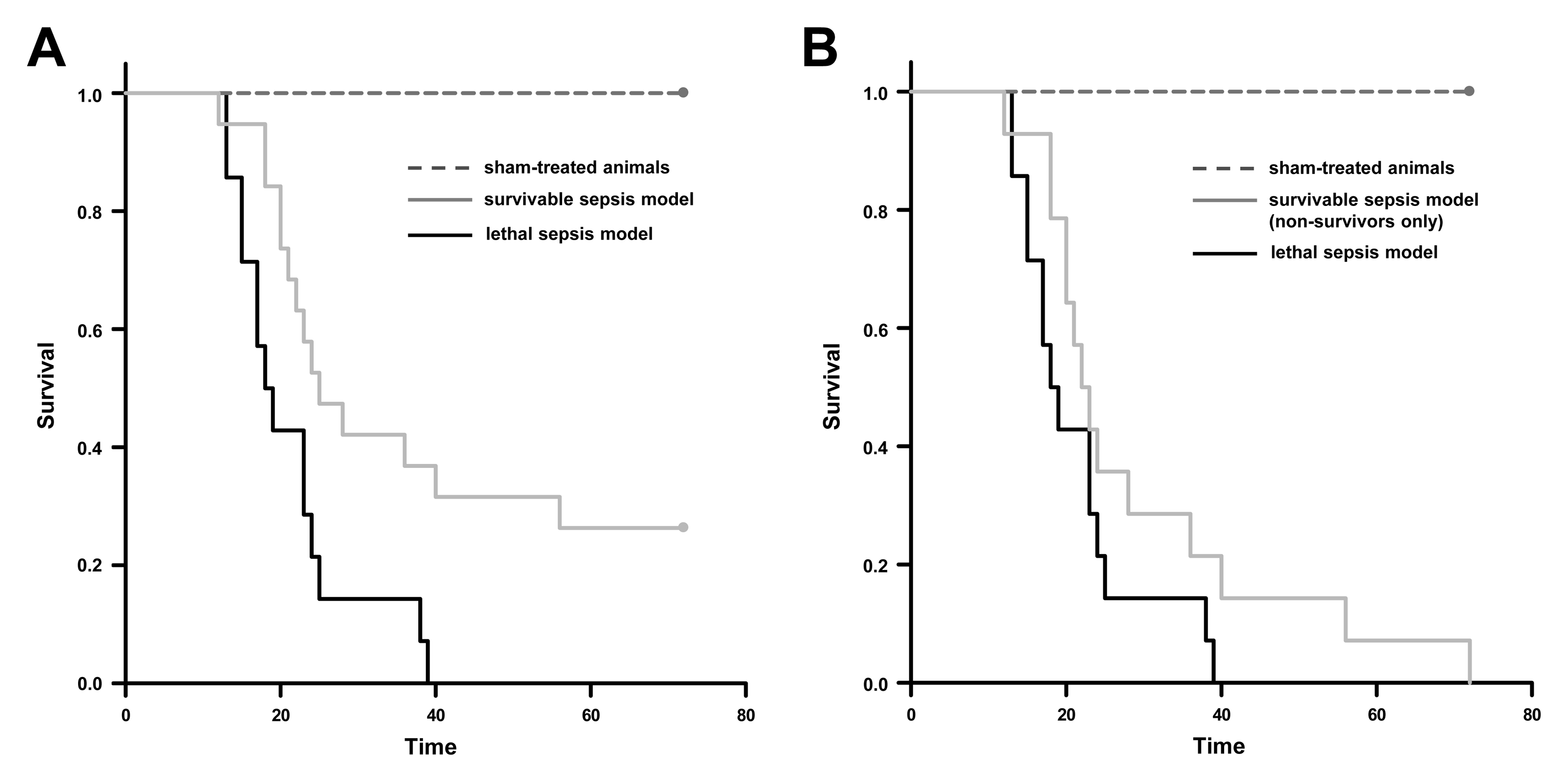

Supplement: Figure S1 — Kaplan-Meier survival analysis of both applied rat sepsis models. (A) Depicted are survival curves for the lethal sepsis model (black line; n = 14), for the survivable sepsis model (grey line; n = 19), and for sham-treated animals (dotted line; n = 14). Log-rank statistic indicates a significant difference between all survival curves (p<0.001). (B) In this survival analysis only the non-survivors (n = 14) in the prognosis-stratified sepsis model are included. Log-rank statistic indicates no significant difference between the survival curves of both sepsis models. (TIF) [file pmed.1001338.s001.tif]

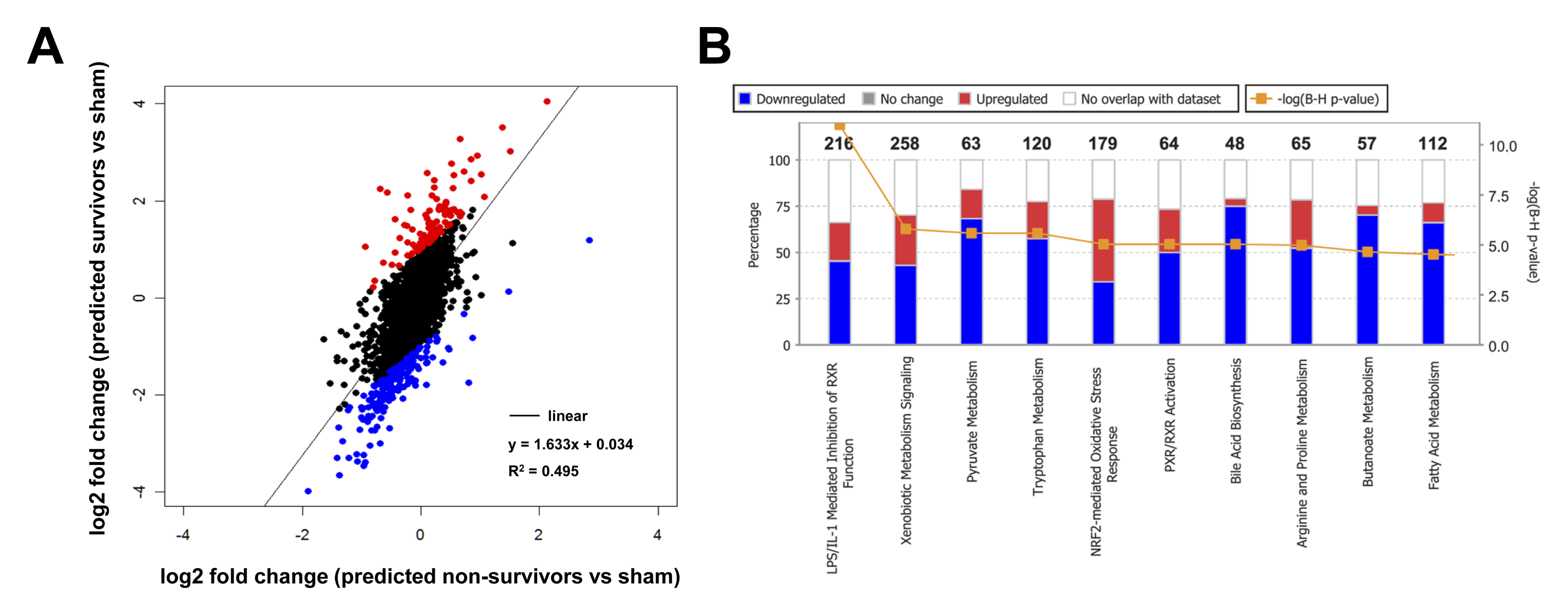

Supplement: Figure S2 — Scatterplot and top ten enriched canonical pathways for the average disparity of transcripts in predicted non-survivors as opposed to predicted survivors. (A) Average log2 fold change for all 11,988 detected transcripts. Red dots: up-regulated more than 2-fold in predicted non-survivors compared to predicted survivors (n = 104). Blue dots: down-regulated more than 2-fold in predicted non-survivors compared to predicted survivors (n = 184). The linear fits for all log2 fold change values exhibit higher amplitudes of expression changes for predicted non-survivors (slope = 1.633). (B) Biplot of top enriched results from Ingenuity Pathway Analysis for the input set of corresponding transcripts (coloured as in [A]). False discovery rate–adjusted p-values after Benjamini-Hochberg (B–H, right axis) were computed using Fisher's exact test accounting for the number of hits, the number of uniquely mapped gene IDs (n = 260), and the total number of entities in the defined pathway (indicated on top of each bar). (TIF) [file pmed.1001338.s002.tif]

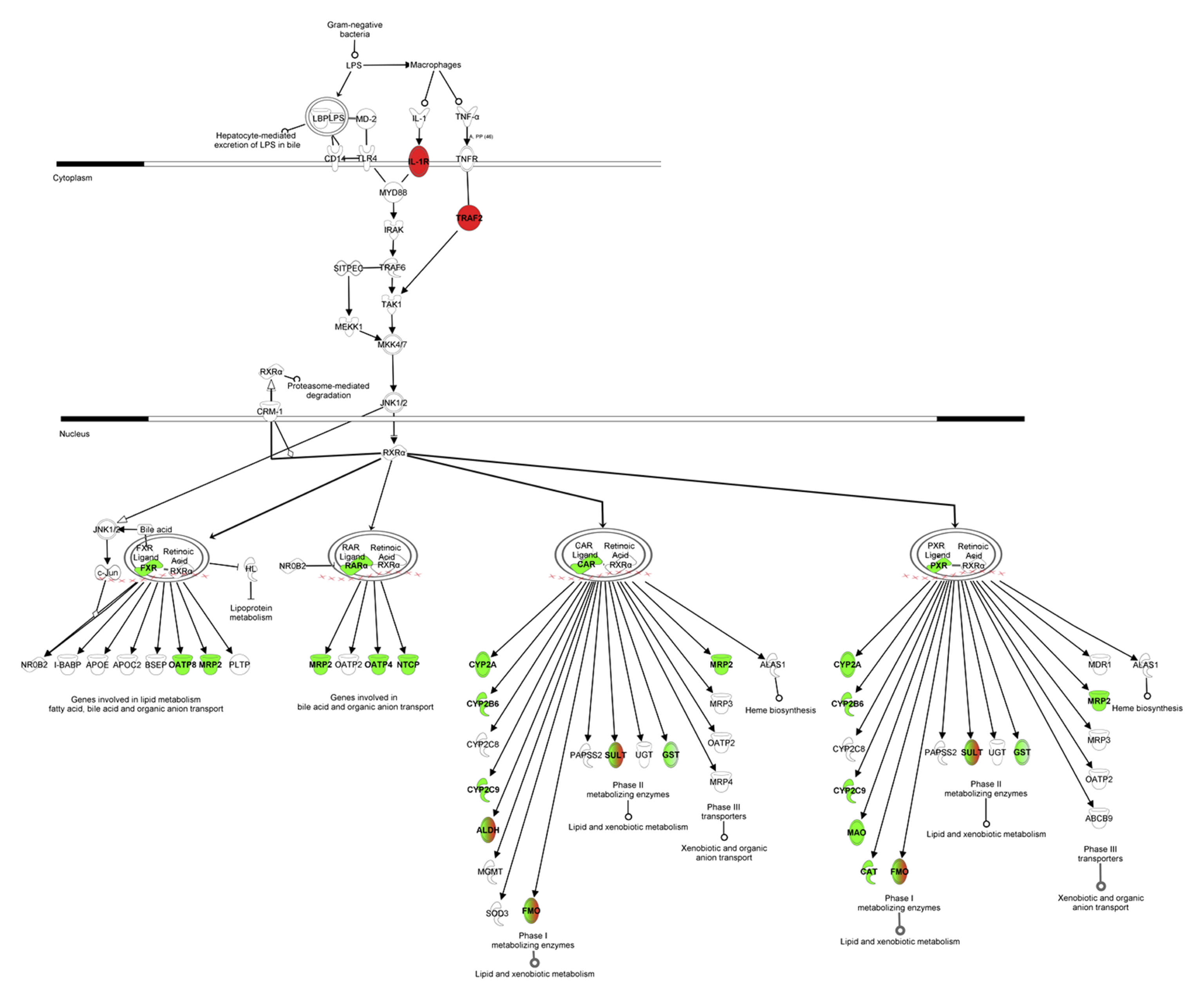

Supplement: Figure S3 — Ingenuity Pathway Analysis of cellular events discriminating predicted non-survivors from predicted survivors. The canonical pathway of biotransformation generated with Ingenuity Pathway Analysis software using data from the clusters “sepsis up-regulated” and “sepsis down-regulated” in the heatmap depicts altered transcripts and presumed interactions of the encoded proteins. Coloured molecules (red = up-regulated; green = down-regulated, red/green = differential regulation of isoforms) represent regulated transcripts. While genes involved in inflammatory signalling (i.e., interleukin-1 receptor [IL-1R] and TNF receptor-associated factor 2 [Traf2]) were up-regulated, the majority of genes participating in cellular metabolic functions showed steady state transcript levels that were substantially decreased in prognosticated septic non-survivors as compared to predicted survivors. Transcript levels encoding critical nuclear receptors, such as farnesoid X receptor (FXR), retinoic acid receptor-α (RARα), constitutive androstane receptor (CAR), and pregnane X receptor (PXR), were coordinately down-regulated. Genes involved in downstream processes responsible for phase I/II metabolism (i.e., cytochrome P450 [CYPs], sulfotransferases [SULTs], and glutathione-S-transferases [GSTs]) and phase III transport (Mrp2, Na+-taurocholate cotransporting polypeptide [Ntcp], and organic anion transporting polypeptides [Oatps]) of endobiotic as well as xenobiotic compounds were affected, with a decreased gene expression associated with poor prognosis. (TIF) [file pmed.1001338.s003.tif]

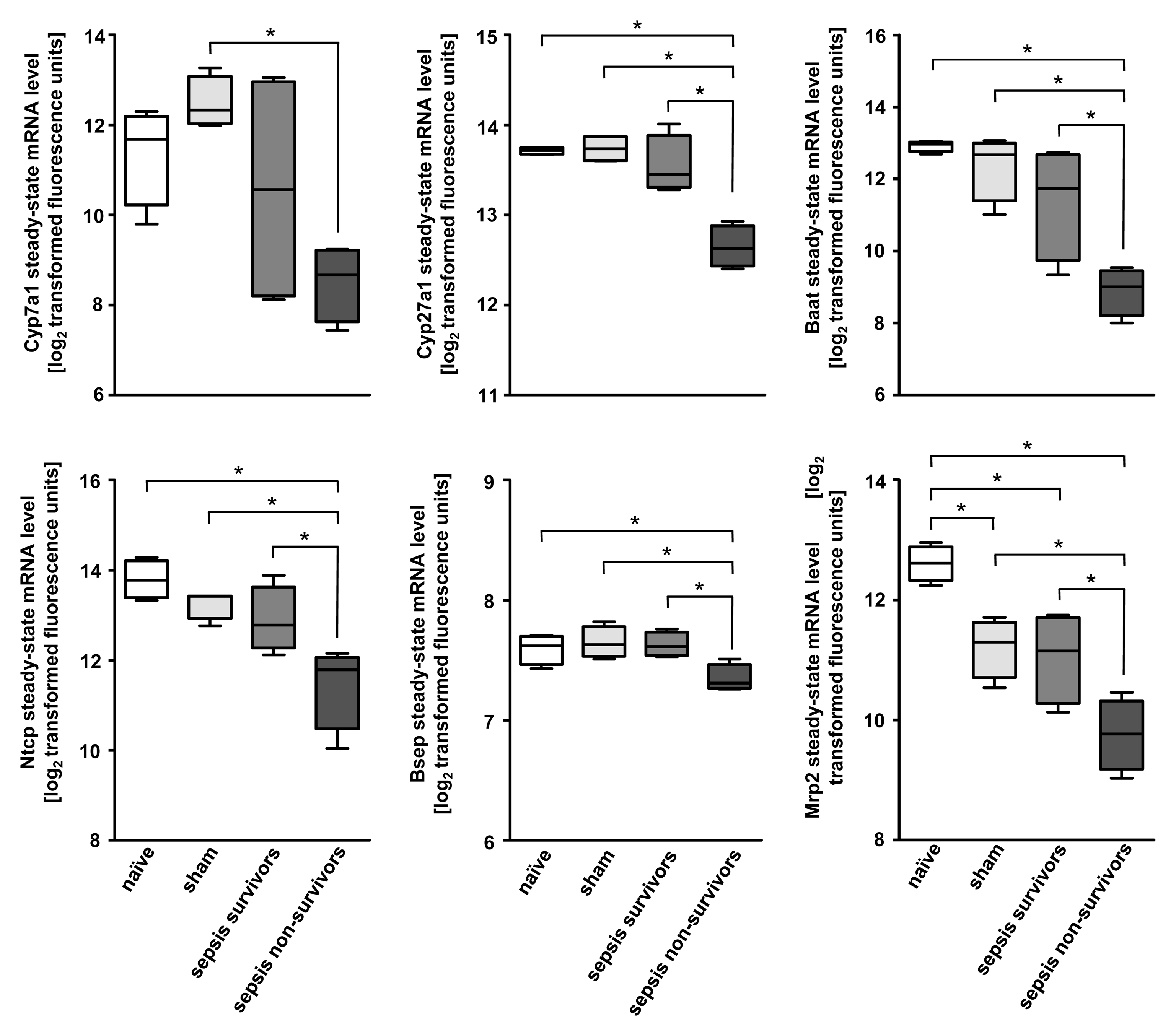

Supplement: Figure S4 — Disease severity–dependent changes of transcripts involved in phase I/II bile acid metabolism and transport. The figure depicts log2 transformed fluorescence units of critical transcripts encoding proteins involved in bile acid biosynthesis and transport—including CYP7a1 (cholesterol-7-alpha-hydroxylase), CYP27a1 (sterol 27-hydroxylase), BAAT, Ntcp (Na+-taurocholate cotransporting polypeptide), Bsep (bile salt export pump), and Mrp2—that were regulated according to disease severity. (TIF) [file pmed.1001338.s004.tif]

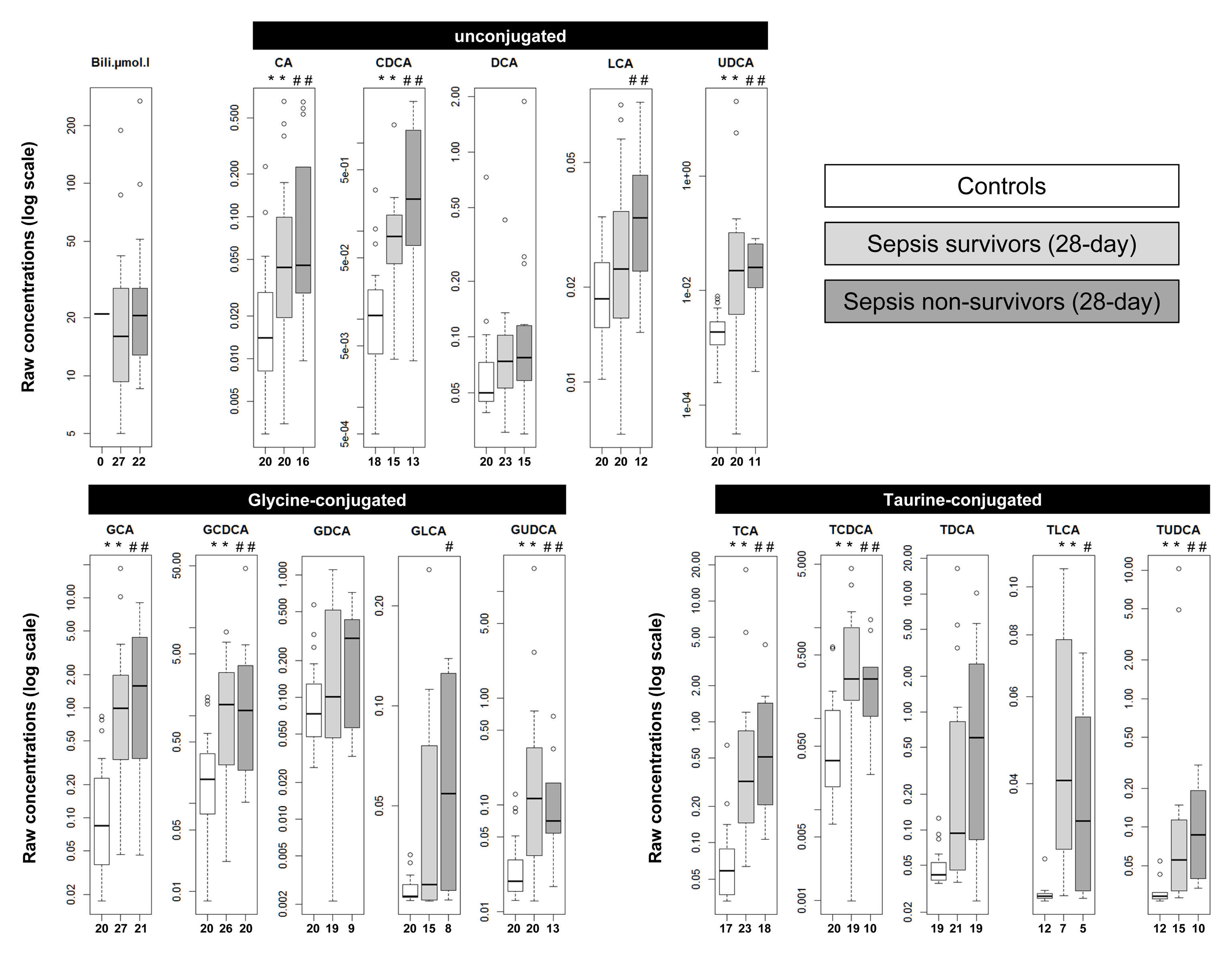

Supplement: Figure S5 — Raw data of bilirubin and unconjugated, glycine-, and taurine-conjugated bile acids stratified according to 28-d outcome. Bilirubin levels are compared to reference control value (21 µmol/l). The number of detected analytes is given below each boxplot. Significance of differences for survivors versus control (*p<0.05, **p<0.01) and non-survivors versus control (# p<0.05; ## p<0.01) is provided below the analyte symbol. (TIF) [file pmed.1001338.s005.tif]
