# Supplementary material for: Liver Dysfunction and Phosphatidylinositol-3-Kinase Signalling in Early Sepsis: Experimental Studies in Rodent Models of Peritonitis
Source: PLoS Med. 2012 Nov 13;9(11):e1001338. doi: 10.1371/journal.pmed.1001338 (PMC3496669; doi:10.1371/journal.pmed.1001338)
Supplement: Table S1 — Full list of up- and down-regulated transcripts out of cluster 1 and 3 for the comparison of predicted non-survivors with predicted survivors. (DOC) [file pmed.1001338.s006.doc]

Supplementary table 1: Full list of up- and down-regulated transcripts out of cluster 1 and 3 for the comparison of prognosticated non-survivors with predicted survivors

| **Cluster** | **Gene regulation** | **Illumina ID** | **Molecules** | **Description** | **Location** | **Type** |
| --- | --- | --- | --- | --- | --- | --- |
| 1 | down | ILMN_58352 | AADAC | arylacetamide deacetylase (esterase) | Cytoplasm | enzyme |
| 1 | down | ILMN_52336 | ABAT | 4-aminobutyrate aminotransferase | Cytoplasm | enzyme |
| 1 | down | ILMN_55670 | ABCA6 | ATP-binding cassette, sub-family A (ABC1), member 6 | Plasma Membrane | transporter |
| 1 | down | ILMN_59060 | ABCB6 | ATP-binding cassette, sub-family B (MDR/TAP), member 6 | Cytoplasm | transporter |
| 1 | down | ILMN_61124 | ABCB7 | ATP-binding cassette, sub-family B (MDR/TAP), member 7 | Cytoplasm | transporter |
| 1 | down | ILMN_53283 | ABCC2 | ATP-binding cassette, sub-family C (CFTR/MRP), member 2 | Plasma Membrane | transporter |
| 1 | down | ILMN_53785 | ABCC6 | ATP-binding cassette, sub-family C (CFTR/MRP), member 6 | Plasma Membrane | transporter |
| 1 | down | ILMN_51315 | ABCD3 | ATP-binding cassette, sub-family D (ALD), member 3 | Cytoplasm | transporter |
| 1 | down | ILMN_48132 | ABCG3 (includes EG:27405) | ATP-binding cassette, sub-family G (WHITE), member 3 | Plasma Membrane | transporter |
| 1 | down | ILMN_51831 | ABHD14B | abhydrolase domain containing 14B | Unknown | enzyme |
| 1 | down | ILMN_51332 | ABHD6 | abhydrolase domain containing 6 | Unknown | enzyme |
| 1 | down | ILMN_64320 | ACAA1 | acetyl-Coenzyme A acyltransferase 1 (peroxisomal 3-oxoacyl-Coenzyme A thiolase) | Cytoplasm | enzyme |
| 1 | down | ILMN_56900 | ACAA2 | acetyl-Coenzyme A acyltransferase 2 (mitochondrial 3-oxoacyl-Coenzyme A thiolase) | Cytoplasm | enzyme |
| 1 | down | ILMN_69215 | ACADL | acyl-Coenzyme A dehydrogenase, long chain | Cytoplasm | enzyme |
| 1 | down | ILMN_63879 | ACADM | acyl-Coenzyme A dehydrogenase, C-4 to Cdown2 straight chain | Cytoplasm | enzyme |
| 1 | down | ILMN_62717 | ACADS | acyl-Coenzyme A dehydrogenase, C-2 to C-3 short chain | Cytoplasm | enzyme |
| 1 | down | ILMN_52763 | ACAT1 | acetyl-Coenzyme A acetyltransferase 1 (acetoacetyl Coenzyme A thiolase) | Cytoplasm | enzyme |
| 1 | down | ILMN_66636 | ACBD5 | acyl-Coenzyme A binding domain containing 5 | Unknown | other |
| 1 | down | ILMN_57843 | ACO1 | aconitase 1, soluble | Cytoplasm | enzyme |
| 1 | down | ILMN_64941 | ACOT12 | acyl-CoA thioesterase 12 | Cytoplasm | enzyme |
| 1 | down | ILMN_64996 | ACOX2 | acyl-Coenzyme A oxidase 2, branched chain | Cytoplasm | enzyme |
| 1 | down | ILMN_67027 | ACSL1 | acyl-CoA synthetase long-chain family member 1 | Cytoplasm | enzyme |
| 1 | down | ILMN_69414 | ACSS2 | acyl-CoA synthetase short-chain family member 2 | Cytoplasm | enzyme |
| 1 | down | ILMN_65165 | ACY1 | aminoacylase 1 | Cytoplasm | peptidase |
| 1 | down | ILMN_54676 | ACY3 | aspartoacylase (aminocyclase) 3 | Unknown | enzyme |
| 1 | down | ILMN_65542 | ADAMTS18 | ADAM metallopeptidase with thrombospondin type 1 motif, 18 | Extracellular Space | peptidase |
| 1 | down | ILMN_63302 | ADCK5 | aarF domain containing kinase 5 | Unknown | kinase |
| 1 | down | ILMN_54287 | ADH1C (includes EG:126) | alcohol dehydrogenase 1C (class I), gamma polypeptide | Cytoplasm | enzyme |
| 1 | down | ILMN_69393 | ADI1 | acireductone dioxygenase 1 | Unknown | enzyme |
| 1 | down | ILMN_62348 | ADORA1 | adenosine A1 receptor | Plasma Membrane | G-protein coupled receptor |
| 1 | down | ILMN_65953 | ADRA1B | adrenergic, alphadownB-, receptor | Plasma Membrane | G-protein coupled receptor |
| 1 | down | ILMN_64594 | AFP | alpha-fetoprotein | Extracellular Space | transporter |
| 1 | down | ILMN_49802 | AGA | aspartylglucosaminidase | Cytoplasm | enzyme |
| 1 | down | ILMN_57216 | AGMAT | agmatine ureohydrolase (agmatinase) | Cytoplasm | enzyme |
| 1 | down | ILMN_48699 | AGPAT2 | 1-acylglycerol-3-phosphate O-acyltransferase 2 (lysophosphatidic acid acyltransferase, beta) | Cytoplasm | enzyme |
| 1 | down | ILMN_56459 | AGTR1 | angiotensin II receptor, type 1 | Plasma Membrane | G-protein coupled receptor |
| 1 | down | ILMN_69991 | AHCY | S-adenosylhomocysteine hydrolase | Cytoplasm | enzyme |
| 1 | down | ILMN_67944 | AHR | aryl hydrocarbon receptor | Nucleus | ligand-dependent nuclear receptor |
| 1 | down | ILMN_59702 | AI182371 | expressed sequence AI182371 | Unknown | other |
| 1 | down | ILMN_64240 | AIFM1 | apoptosis-inducing factor, mitochondrion-associated, 1 | Cytoplasm | enzyme |
| 1 | down | ILMN_51770 | AIFM2 | apoptosis-inducing factor, mitochondrion-associated, 2 | Cytoplasm | enzyme |
| 1 | down | ILMN_48870 | AIM1L | absent in melanoma 1-like | Unknown | other |
| 1 | down | ILMN_57354 | AKAP1 | A kinase (PRKA) anchor protein 1 | Cytoplasm | other |
| 1 | down | ILMN_56158 | AKR1C14 | aldo-keto reductase family 1, member C14 | Cytoplasm | enzyme |
| 1 | down | ILMN_52580 | AKR1C3 | aldo-keto reductase family 1, member C3 (3-alpha hydroxysteroid dehydrogenase, type II) | Cytoplasm | enzyme |
| 1 | down | ILMN_51299 | AKR1CL2 | aldo-keto reductase family 1, member C-like 2 | Cytoplasm | enzyme |
| 1 | down | ILMN_60608 | AKR1D1 | aldo-keto reductase family 1, member D1 (delta 4-3-ketosteroid-5-beta-reductase) | Cytoplasm | enzyme |
| 1 | down | ILMN_56623 | AKR7A2 | aldo-keto reductase family 7, member A2 (aflatoxin aldehyde reductase) | Cytoplasm | enzyme |
| 1 | down | ILMN_49467 | ALAD | aminolevulinate, delta-, dehydratase | Cytoplasm | enzyme |
| 1 | down | ILMN_48166 | ALDH1A1 | aldehyde dehydrogenase 1 family, member A1 | Cytoplasm | enzyme |
| 1 | down | ILMN_55014 | ALDH3A2 | aldehyde dehydrogenase 3 family, member A2 | Cytoplasm | enzyme |
| 1 | down | ILMN_56609 | ALDH5A1 | aldehyde dehydrogenase 5 family, member A1 (succinate-semialdehyde dehydrogenase) | Cytoplasm | enzyme |
| 1 | down | ILMN_66389 | ALDH8A1 PREDICTED | aldehyde dehydrogenase 8 family, member A1 (predicted) | Unknown | enzyme |
| 1 | down | ILMN_68429 | ALDH9A1 | aldehyde dehydrogenase 9 family, member A1 | Cytoplasm | enzyme |
| 1 | down | ILMN_64417 | ALDOB | aldolase B, fructose-bisphosphate | Cytoplasm | enzyme |
| 1 | down | ILMN_48048 | AMT (includes EG:275) | aminomethyltransferase | Cytoplasm | enzyme |
| 1 | down | ILMN_57016 | AMY2A | amylase, alpha 2A (pancreatic) | Extracellular Space | enzyme |
| 1 | down | ILMN_56782 | ANAPC4 | anaphase promoting complex subunit 4 | Unknown | enzyme |
| 1 | down | ILMN_49101 | ANKRD15 | ankyrin repeat domain 15 | Nucleus | transcription regulator |
| 1 | down | ILMN_52564 | ANXA11 | annexin A11 | Nucleus | other |
| 1 | down | ILMN_63561 | AP1GBP1 | AP1 gamma subunit binding protein 1 | Cytoplasm | other |
| 1 | down | ILMN_62308 | APOA1BP | apolipoprotein A-I binding protein | Extracellular Space | other |
| 1 | down | ILMN_51822 | APOF | apolipoprotein F | Extracellular Space | transporter |
| 1 | down | ILMN_59294 | APOL3 (includes EG:315108) | apolipoprotein L, 3 | Unknown | transporter |
| 1 | down | ILMN_67993 | AQP11 | aquaporin 11 | Unknown | transporter |
| 1 | down | ILMN_48890 | ARHGAP24 | Rho GTPase activating protein 24 | Cytoplasm | other |
| 1 | down | ILMN_55650 | ARHGEF19 | Rho guanine nucleotide exchange factor (GEF) 19 | Unknown | other |
| 1 | down | ILMN_51780 | ATP5C1 | ATP synthase, H+ transporting, mitochondrial F1 complex, gamma polypeptide 1 | Cytoplasm | transporter |
| 1 | down | ILMN_59596 | ATP5I | ATP synthase, H+ transporting, mitochondrial F0 complex, subunit E | Cytoplasm | transporter |
| 1 | down | ILMN_54318 | ATP5O | ATP synthase, H+ transporting, mitochondrial F1 complex, O subunit (oligomycin sensitivity conferring protein) | Cytoplasm | transporter |
| 1 | down | ILMN_63704 | ATP6V0E2 | ATPase, H+ transporting V0 subunit e2 | Unknown | enzyme |
| 1 | down | ILMN_52052 | AUH | AU RNA binding protein/enoyl-Coenzyme A hydratase | Cytoplasm | enzyme |
| 1 | down | ILMN_49987 | AVPR1A | arginine vasopressin receptor 1A | Plasma Membrane | G-protein coupled receptor |
| 1 | down | ILMN_56365 | BAAT | bile acid Coenzyme A: amino acid N-acyltransferase (glycine N-choloyltransferase) | Cytoplasm | enzyme |
| 1 | down | ILMN_49962 | BAT5 | HLA-B associated transcript 5 | Unknown | other |
| 1 | down | ILMN_60493 | BBOX1 | butyrobetaine (gamma), 2-oxoglutarate dioxygenase (gamma-butyrobetaine hydroxylase) 1 | Cytoplasm | enzyme |
| 1 | down | ILMN_53371 | BC021614 | cDNA sequence BC021614 | Unknown | other |
| 1 | down | ILMN_51120 | BCAR1 | breast cancer anti-estrogen resistance 1 | Plasma Membrane | other |
| 1 | down | ILMN_64510 | BCKDHA | branched chain keto acid dehydrogenase E1, alpha polypeptide | Cytoplasm | enzyme |
| 1 | down | ILMN_60448 | BCKDHB | branched chain keto acid dehydrogenase E1, beta polypeptide (maple syrup urine disease) | Cytoplasm | enzyme |
| 1 | down | ILMN_65020 | BDH1 | 3-hydroxybutyrate dehydrogenase, type 1 | Cytoplasm | enzyme |
| 1 | down | ILMN_50334 | BHMT | betaine-homocysteine methyltransferase | Cytoplasm | enzyme |
| 1 | down | ILMN_56608 | BPHL | biphenyl hydrolase-like (serine hydrolase; breast epithelial mucin-associated antigen) | Cytoplasm | enzyme |
| 1 | down | ILMN_51904 | BUCS1 PREDICTED | butyryl Coenzyme A synthetase 1 (predicted) | Cytoplasm | enzyme |
| 1 | down | ILMN_63898 | C10ORF32 | chromosome 10 open reading frame 32 | Unknown | other |
| 1 | down | ILMN_51302 | C10ORF33 | chromosome 10 open reading frame 33 | Unknown | other |
| 1 | down | ILMN_52152 | C10ORF35 | chromosome 10 open reading frame 35 | Unknown | other |
| 1 | down | ILMN_59519 | C10ORF65 | chromosome 10 open reading frame 65 | Cytoplasm | other |
| 1 | down | ILMN_60716 | C11ORF60 | chromosome 11 open reading frame 60 | Cytoplasm | other |
| 1 | down | ILMN_62213 | C12ORF10 | chromosome 12 open reading frame 10 | Unknown | other |
| 1 | down | ILMN_50993 | C12ORF26 | chromosome 12 open reading frame 26 | Unknown | other |
| 1 | down | ILMN_65504 | C14ORF68 | chromosome 14 open reading frame 68 | Cytoplasm | other |
| 1 | down | ILMN_53038 | C16ORF30 | chromosome 16 open reading frame 30 | Plasma Membrane | other |
| 1 | down | ILMN_68582 | C16ORF5 | chromosome 16 open reading frame 5 | Unknown | other |
| 1 | down | ILMN_50988 | C1ORF128 | chromosome 1 open reading frame 128 | Unknown | other |
| 1 | down | ILMN_49972 | C1ORF172 | chromosome 1 open reading frame 172 | Unknown | other |
| 1 | down | ILMN_49966 | C1ORF57 | chromosome 1 open reading frame 57 | Unknown | other |
| 1 | down | ILMN_69434 | C22ORF16 | chromosome 22 open reading frame 16 | Unknown | other |
| 1 | down | ILMN_55117 | C22ORF25 | chromosome 22 open reading frame 25 | Unknown | other |
| 1 | down | ILMN_58400 | C2ORF28 | chromosome 2 open reading frame 28 | Unknown | other |
| 1 | down | ILMN_51951 | C4ORF19 | chromosome 4 open reading frame 19 | Unknown | other |
| 1 | down | ILMN_51129 | C6ORF108 | chromosome 6 open reading frame 108 | Nucleus | other |
| 1 | down | ILMN_56157 | C6ORF145 | chromosome 6 open reading frame 145 | Unknown | other |
| 1 | down | ILMN_63823 | C6ORF162 | chromosome 6 open reading frame 162 | Unknown | other |
| 1 | down | ILMN_50879 | C9ORF46 | chromosome 9 open reading frame 46 | Unknown | other |
| 1 | down | ILMN_48268 | CA14 | carbonic anhydrase XIV | Plasma Membrane | enzyme |
| 1 | down | ILMN_58560 | CA5A | carbonic anhydrase VA, mitochondrial | Cytoplasm | enzyme |
| 1 | down | ILMN_52222 | CABC1 | chaperone, ABC1 activity of bc1 complex homolog (S. pombe) | Cytoplasm | kinase |
| 1 | down | ILMN_53457 | CABP2 | calcium binding protein 2 | Unknown | other |
| 1 | down | ILMN_48416 | CALD1 | caldesmon 1 | Cytoplasm | other |
| 1 | down | ILMN_63406 | CARS2 | cysteinyl-tRNA synthetase 2, mitochondrial (putative) | Unknown | enzyme |
| 1 | down | ILMN_56091 | CAT | catalase | Cytoplasm | enzyme |
| 1 | down | ILMN_62437 | CBR4 | carbonyl reductase 4 | Unknown | enzyme |
| 1 | down | ILMN_69621 | CCS | copper chaperone for superoxide dismutase | Cytoplasm | enzyme |
| 1 | down | ILMN_53809 | CD36 | CD36 molecule (thrombospondin receptor) | Plasma Membrane | other |
| 1 | down | ILMN_55333 | CD82 | CD82 molecule | Plasma Membrane | other |
| 1 | down | ILMN_67196 | CETN2 | centrin, EF-hand protein, 2 | Nucleus | enzyme |
| 1 | down | ILMN_68234 | CIDEB | cell death-inducing DFFA-like effector b | Cytoplasm | other |
| 1 | down | ILMN_49581 | CLDN1 | claudin 1 | Plasma Membrane | other |
| 1 | down | ILMN_64855 | CMBL | carboxymethylenebutenolidase homolog (Pseudomonas) | Unknown | enzyme |
| 1 | down | ILMN_53709 | CMTM8 | CKLF-like MARVEL transmembrane domain containing 8 | Extracellular Space | cytokine |
| 1 | down | ILMN_63412 | COQ9 | coenzyme Q9 homolog (S. cerevisiae) | Cytoplasm | other |
| 1 | down | ILMN_64731 | CPS1 | carbamoyl-phosphate synthetase 1, mitochondrial | Cytoplasm | enzyme |
| 1 | down | ILMN_69382 | CPT2 | carnitine palmitoyltransferase II | Cytoplasm | enzyme |
| 1 | down | ILMN_58547 | CROT | carnitine O-octanoyltransferase | Cytoplasm | enzyme |
| 1 | down | ILMN_51307 | CRYL1 | crystallin, lambda 1 | Unknown | other |
| 1 | down | ILMN_62814 | CSAD | cysteine sulfinic acid decarboxylase | Unknown | enzyme |
| 1 | down | ILMN_67413 | CTH | cystathionase (cystathionine gamma-lyase) | Cytoplasm | enzyme |
| 1 | down | ILMN_53125 | CUL1 | cullin 1 | Nucleus | enzyme |
| 1 | down | ILMN_62173 | CUL2 | cullin 2 | Nucleus | enzyme |
| 1 | down | ILMN_59478 | CUL4A | cullin 4A | Nucleus | other |
| 1 | down | ILMN_58464 | CUTC | cutC copper transporter homolog (E. coli) | Unknown | other |
| 1 | down | ILMN_54776 | CYB5R3 | cytochrome b5 reductase 3 | Cytoplasm | enzyme |
| 1 | down | ILMN_64821 | CYP27A1 | cytochrome P450, family 27, subfamily A, polypeptide 1 | Cytoplasm | enzyme |
| 1 | down | ILMN_59498 | CYP2A2 | cytochrome P450, subfamily 2A, polypeptide 1 | Cytoplasm | enzyme |
| 1 | down | ILMN_65398 | CYP2B6 (includes EG:1555) | cytochrome P450, family 2, subfamily B, polypeptide 6 | Cytoplasm | enzyme |
| 1 | down | ILMN_56711 | CYP2B9 | cytochrome P450, family 2, subfamily b, polypeptide 9 | Cytoplasm | enzyme |
| 1 | down | ILMN_60675 | CYP2C9 | cytochrome P450, family 2, subfamily C, polypeptide 9 | Cytoplasm | enzyme |
| 1 | down | ILMN_49996 | CYP2R1 | cytochrome P450, family 2, subfamily R, polypeptide 1 | Cytoplasm | enzyme |
| 1 | down | ILMN_59276 | D730039F16RIK | RIKEN cDNA D730039F16 gene | Unknown | other |
| 1 | down | ILMN_54865 | DAB2IP | DAB2 interacting protein | Plasma Membrane | other |
| 1 | down | ILMN_57638 | DAO | D-amino-acid oxidase | Cytoplasm | enzyme |
| 1 | down | ILMN_67468 | DDT (includes EG:1652) | D-dopachrome tautomerase | Cytoplasm | enzyme |
| 1 | down | ILMN_69996 | DECR2 | 2,4-dienoyl CoA reductase 2, peroxisomal | Cytoplasm | enzyme |
| 1 | down | ILMN_61157 | DEPDC7 | DEP domain containing 7 | Unknown | other |
| 1 | down | ILMN_64139 | DGKA | diacylglycerol kinase, alpha 80kDa | Cytoplasm | kinase |
| 1 | down | ILMN_55799 | DHTKD1 | dehydrogenase E1 and transketolase domain containing 1 | Unknown | enzyme |
| 1 | down | ILMN_48800 | DPY19L1 | dpydown9-like 1 (C. elegans) | Unknown | other |
| 1 | down | ILMN_49157 | DPYS | dihydropyrimidinase | Cytoplasm | enzyme |
| 1 | down | ILMN_57279 | DUT (includes EG:1854) | deoxyuridine triphosphatase | Nucleus | enzyme |
| 1 | down | ILMN_56270 | DVL1 | dishevelled, dsh homolog 1 (Drosophila) | Cytoplasm | other |
| 1 | down | ILMN_49351 | ECHDC2 | enoyl Coenzyme A hydratase domain containing 2 | Unknown | other |
| 1 | down | ILMN_68949 | ECHS1 | enoyl Coenzyme A hydratase, short chain, 1, mitochondrial | Cytoplasm | enzyme |
| 1 | down | ILMN_64789 | EG240549 | predicted gene, EG240549 | Unknown | other |
| 1 | down | ILMN_55723 | EIF2B4 | eukaryotic translation initiation factor 2B, subunit 4 delta, 67kDa | Cytoplasm | translation regulator |
| 1 | down | ILMN_67501 | ENPP2 | ectonucleotide pyrophosphatase/phosphodiesterase 2 (autotaxin) | Plasma Membrane | enzyme |
| 1 | down | ILMN_58634 | ENTPD5 | ectonucleoside triphosphate diphosphohydrolase 5 | Cytoplasm | enzyme |
| 1 | down | ILMN_67462 | EPHX2 | epoxide hydrolase 2, cytoplasmic | Cytoplasm | enzyme |
| 1 | down | ILMN_50149 | ETFDH | electron-transferring-flavoprotein dehydrogenase | Cytoplasm | enzyme |
| 1 | down | ILMN_62078 | ETNK2 | ethanolamine kinase 2 | Unknown | kinase |
| 1 | down | ILMN_69716 | F11R | F11 receptor | Plasma Membrane | other |
| 1 | down | ILMN_70140 | F13B | coagulation factor XIII, B polypeptide | Cytoplasm | enzyme |
| 1 | down | ILMN_69138 | FAAH | fatty acid amide hydrolase | Plasma Membrane | enzyme |
| 1 | down | ILMN_53865 | FAH | fumarylacetoacetate hydrolase (fumarylacetoacetase) | Cytoplasm | enzyme |
| 1 | down | ILMN_57316 | FAHD1 | fumarylacetoacetate hydrolase domain containing 1 | Cytoplasm | enzyme |
| 1 | down | ILMN_66061 | FAM69A | family with sequence similarity 69, member A | Unknown | other |
| 1 | down | ILMN_60074 | FAM82B | family with sequence similarity 82, member B | Unknown | other |
| 1 | down | ILMN_54196 | FBXO9 | F-box protein 9 | Cytoplasm | enzyme |
| 1 | down | ILMN_54160 | FDX1 | ferredoxin 1 | Cytoplasm | transporter |
| 1 | down | ILMN_161856 | FECH | ferrochelatase (protoporphyria) | Cytoplasm | enzyme |
| 1 | down | ILMN_62623 | FECH | ferrochelatase (protoporphyria) | Cytoplasm | enzyme |
| 1 | down | ILMN_66172 | FIG4 | FIG4 homolog (S. cerevisiae) | Unknown | other |
| 1 | down | ILMN_63138 | FLJ11151 | hypothetical protein FLJ11151 | Unknown | enzyme |
| 1 | down | ILMN_61469 | FLJ37464 | hypothetical protein FLJ37464 | Extracellular Space | enzyme |
| 1 | down | ILMN_69655 | FMO1 | flavin containing monooxygenase 1 | Cytoplasm | enzyme |
| 1 | down | ILMN_51217 | FMO4 | flavin containing monooxygenase 4 | Cytoplasm | enzyme |
| 1 | down | ILMN_68828 | FN3KRP | fructosamine-3-kinase-related protein | Unknown | kinase |
| 1 | down | ILMN_69833 | FTCD | formiminotransferase cyclodeaminase | Cytoplasm | enzyme |
| 1 | down | ILMN_57428 | FUNDC1 | FUN14 domain containing 1 | Unknown | other |
| 1 | down | ILMN_63408 | FZD8 | frizzled homolog 8 (Drosophila) | Plasma Membrane | G-protein coupled receptor |
| 1 | down | ILMN_51221 | GALK1 | galactokinase 1 | Cytoplasm | kinase |
| 1 | down | ILMN_64854 | GALM | galactose mutarotase (aldose 1-epimerase) | Cytoplasm | enzyme |
| 1 | down | ILMN_64599 | GAMT | guanidinoacetate N-methyltransferase | Cytoplasm | enzyme |
| 1 | down | ILMN_66203 | GAS2 | growth arrest-specific 2 | Cytoplasm | other |
| 1 | down | ILMN_55866 | GBE1 | glucan (1,4-alpha-), branching enzyme 1 (glycogen branching enzyme, Andersen disease, glycogen storage disease type IV) | Cytoplasm | enzyme |
| 1 | down | ILMN_58545 | GBL | G protein beta subunit-like | Unknown | other |
| 1 | down | ILMN_54159 | GCGR | glucagon receptor | Plasma Membrane | G-protein coupled receptor |
| 1 | down | ILMN_58986 | GCGR | glucagon receptor | Plasma Membrane | G-protein coupled receptor |
| 1 | down | ILMN_65390 | GCLM | glutamate-cysteine ligase, modifier subunit | Cytoplasm | enzyme |
| 1 | down | ILMN_62002 | GCSH | glycine cleavage system protein H (aminomethyl carrier) | Cytoplasm | enzyme |
| 1 | down | ILMN_65439 | GDPD1 | glycerophosphodiester phosphodiesterase domain containing 1 | Unknown | enzyme |
| 1 | down | ILMN_52217 | GGNBP2 | gametogenetin binding protein 2 | Unknown | other |
| 1 | down | ILMN_69129 | GJB1 | gap junction protein, beta 1, 32kDa | Plasma Membrane | transporter |
| 1 | down | ILMN_51906 | GLDC | glycine dehydrogenase (decarboxylating) | Cytoplasm | enzyme |
| 1 | down | ILMN_62601 | GLTPD2 | glycolipid transfer protein domain containing 2 | Unknown | other |
| 1 | down | ILMN_59253 | GLUD1 | glutamate dehydrogenase 1 | Cytoplasm | enzyme |
| 1 | down | ILMN_54049 | GLYAT | glycine-N-acyltransferase | Cytoplasm | enzyme |
| 1 | down | ILMN_69634 | GNMT | glycine N-methyltransferase | Cytoplasm | enzyme |
| 1 | down | ILMN_66450 | GOLPH2 PREDICTED | golgi phosphoprotein 2 (predicted) | Unknown | other |
| 1 | down | ILMN_61988 | GOLT1A | golgi transport 1 homolog A (S. cerevisiae) | Unknown | other |
| 1 | down | ILMN_68085 | GPAM | glycerol-3-phosphate acyltransferase, mitochondrial | Cytoplasm | enzyme |
| 1 | down | ILMN_53363 | GPD1 | glycerol-3-phosphate dehydrogenase 1 (soluble) | Cytoplasm | enzyme |
| 1 | down | ILMN_59628 | GPHN | gephyrin | Plasma Membrane | enzyme |
| 1 | down | ILMN_56869 | GPRC5C | G protein-coupled receptor, family C, group 5, member C | Plasma Membrane | G-protein coupled receptor |
| 1 | down | ILMN_58885 | GPSN2 | glycoprotein, synaptic 2 | Plasma Membrane | other |
| 1 | down | ILMN_59822 | GRAMD1C | GRAM domain containing 1C | Unknown | other |
| 1 | down | ILMN_54369 | GSTA4 | glutathione S-transferase A4 | Cytoplasm | enzyme |
| 1 | down | ILMN_62378 | GSTA5 | glutathione S-transferase A5 | Cytoplasm | enzyme |
| 1 | down | ILMN_66837 | GSTA5 | glutathione S-transferase A5 | Cytoplasm | enzyme |
| 1 | down | ILMN_68593 | GSTM2 | glutathione S-transferase M2 (muscle) | Cytoplasm | enzyme |
| 1 | down | ILMN_59083 | GSTT2 | glutathione S-transferase theta 2 | Cytoplasm | enzyme |
| 1 | down | ILMN_67518 | GTF2I | general transcription factor II, i | Nucleus | transcription regulator |
| 1 | down | ILMN_60583 | GTF3C1 | general transcription factor IIIC, polypeptide 1, alpha 220kDa | Nucleus | transcription regulator |
| 1 | down | ILMN_53109 | GUSB | glucuronidase, beta | Cytoplasm | enzyme |
| 1 | down | ILMN_65984 | HAAO | 3-hydroxyanthranilate 3,4-dioxygenase | Cytoplasm | enzyme |
| 1 | down | ILMN_64662 | HABP4 | hyaluronan binding protein 4 | Cytoplasm | other |
| 1 | down | ILMN_49010 | HACL1 | 2-hydroxyacyl-CoA lyase 1 | Cytoplasm | enzyme |
| 1 | down | ILMN_60853 | HADH | hydroxyacyl-Coenzyme A dehydrogenase | Cytoplasm | enzyme |
| 1 | down | ILMN_59192 | HAO1 | hydroxyacid oxidase (glycolate oxidase) 1 | Cytoplasm | enzyme |
| 1 | down | ILMN_67806 | HAO2 (includes EG:51179) | hydroxyacid oxidase 2 (long chain) | Cytoplasm | enzyme |
| 1 | down | ILMN_64577 | HDAC8 | histone deacetylase 8 | Nucleus | transcription regulator |
| 1 | down | ILMN_53816 | HDDC3 | HD domain containing 3 | Unknown | other |
| 1 | down | ILMN_48467 | HECTD1 | HECT domain containing 1 | Unknown | enzyme |
| 1 | down | ILMN_67510 | HES6 | hairy and enhancer of split 6 (Drosophila) | Nucleus | transcription regulator |
| 1 | down | ILMN_61719 | HGD | homogentisate 1,2-dioxygenase (homogentisate oxidase) | Cytoplasm | enzyme |
| 1 | down | ILMN_47714 | HINT3 | histidine triad nucleotide binding protein 3 | Unknown | other |
| 1 | down | ILMN_60404 | HMGCS2 | 3-hydroxy-3-methylglutaryl-Coenzyme A synthase 2 (mitochondrial) | Cytoplasm | enzyme |
| 1 | down | ILMN_55971 | HPN | hepsin (transmembrane protease, serine 1) | Plasma Membrane | peptidase |
| 1 | down | ILMN_69903 | HSD17B11 | hydroxysteroid (17-beta) dehydrogenase 11 | Cytoplasm | enzyme |
| 1 | down | ILMN_67645 | HSD17B7 | hydroxysteroid (17-beta) dehydrogenase 7 | Cytoplasm | enzyme |
| 1 | down | ILMN_62557 | HSD3B5 | hydroxy-delta-5-steroid dehydrogenase, 3 beta- and steroid delta-isomerase 5 | Cytoplasm | enzyme |
| 1 | down | ILMN_64589 | HSD3B7 | hydroxy-delta-5-steroid dehydrogenase, 3 beta- and steroid delta-isomerase 7 | Cytoplasm | enzyme |
| 1 | down | ILMN_50766 | IDH1 | isocitrate dehydrogenase 1 (NADP+), soluble | Cytoplasm | enzyme |
| 1 | down | ILMN_53112 | IDH2 | isocitrate dehydrogenase 2 (NADP+), mitochondrial | Cytoplasm | enzyme |
| 1 | down | ILMN_56655 | IGF2BP3 | insulin-like growth factor 2 mRNA binding protein 3 | Nucleus | other |
| 1 | down | ILMN_62027 | IGFALS | insulin-like growth factor binding protein, acid labile subunit | Extracellular Space | other |
| 1 | down | ILMN_59153 | IGFBP3 | insulin-like growth factor binding protein 3 | Extracellular Space | other |
| 1 | down | ILMN_54831 | IGSF5 | immunoglobulin superfamily, member 5 | Plasma Membrane | other |
| 1 | down | ILMN_66947 | IL1F8 | interleukin 1 family, member 8 (eta) | Extracellular Space | cytokine |
| 1 | down | ILMN_61229 | INHBA | inhibin, beta A | Extracellular Space | growth factor |
| 1 | down | ILMN_60184 | IQWD1 | IQ motif and WD repeats 1 | Nucleus | other |
| 1 | down | ILMN_60461 | IRF6 | interferon regulatory factor 6 | Nucleus | transcription regulator |
| 1 | down | ILMN_66522 | IRGM | immunity-related GTPase family, M | Cytoplasm | other |
| 1 | down | ILMN_60108 | ISOC1 | isochorismatase domain containing 1 | Cytoplasm | enzyme |
| 1 | down | ILMN_64469 | ITGB5 | integrin, beta 5 | Plasma Membrane | other |
| 1 | down | ILMN_60666 | IVD | isovaleryl Coenzyme A dehydrogenase | Cytoplasm | enzyme |
| 1 | down | ILMN_54956 | JUB | jub, ajuba homolog (Xenopus laevis) | Plasma Membrane | other |
| 1 | down | ILMN_62029 | JUP | junction plakoglobin | Plasma Membrane | other |
| 1 | down | ILMN_57487 | KHK | ketohexokinase (fructokinase) | Cytoplasm | kinase |
| 1 | down | ILMN_49217 | KLB | klotho beta | Unknown | enzyme |
| 1 | down | ILMN_67539 | KLC4 | kinesin light chain 4 | Unknown | other |
| 1 | down | ILMN_63431 | KLRG1 | killer cell lectin-like receptor subfamily G, member 1 | Plasma Membrane | other |
| 1 | down | ILMN_69227 | KMO | kynurenine 3-monooxygenase (kynurenine 3-hydroxylase) | Cytoplasm | enzyme |
| 1 | down | ILMN_68261 | KPNA2 | karyopherin alpha 2 (RAG cohort 1, importin alpha 1) | Nucleus | transporter |
| 1 | down | ILMN_52260 | KRT19 | keratin 19 | Cytoplasm | other |
| 1 | down | ILMN_65973 | KYNU | kynureninase (L-kynurenine hydrolase) | Cytoplasm | enzyme |
| 1 | down | ILMN_50985 | LACTB2 | lactamase, beta 2 | Cytoplasm | other |
| 1 | down | ILMN_57934 | LAG3 | lymphocyte-activation gene 3 | Plasma Membrane | transmembrane receptor |
| 1 | down | ILMN_51502 | LCMT1 | leucine carboxyl methyltransferase 1 | Unknown | enzyme |
| 1 | down | ILMN_66014 | LHPP | phospholysine phosphohistidine inorganic pyrophosphate phosphatase | Unknown | phosphatase |
| 1 | down | ILMN_49136 | LIAS | lipoic acid synthetase | Cytoplasm | enzyme |
| 1 | down | ILMN_65547 | LIMK2 | LIM domain kinase 2 | Cytoplasm | kinase |
| 1 | down | ILMN_59125 | LOC116236 | hypothetical protein LOC116236 | Extracellular Space | other |
| 1 | down | ILMN_161443 | LOC143941 | similar to CDNA sequence BC021608 | Unknown | other |
| 1 | down | ILMN_65700 | LOC143941 | similar to CDNA sequence BC021608 | Unknown | other |
| 1 | down | ILMN_68572 | LOC364773 | similar to liver regeneration-related protein LRRG07 | Unknown | other |
| 1 | down | ILMN_56415 | LOC388335 | similar to RIKEN cDNA A730055C05 gene | Unknown | other |
| 1 | down | ILMN_61406 | LOC57228 | small trans-membrane and glycosylated protein | Unknown | other |
| 1 | down | ILMN_54512 | LOC645619 | similar to Adenylate kinase isoenzyme 4, mitochondrial (ATP-AMP transphosphorylase) | Unknown | other |
| 1 | down | ILMN_69893 | LONP2 | lon peptidase 2, peroxisomal | Cytoplasm | peptidase |
| 1 | down | ILMN_55875 | LPHN1 | latrophilin 1 | Plasma Membrane | G-protein coupled receptor |
| 1 | down | ILMN_60603 | LRRC40 | leucine rich repeat containing 40 | Nucleus | other |
| 1 | down | ILMN_55304 | LYPLAL1 | lysophospholipase-like 1 | Cytoplasm | enzyme |
| 1 | down | ILMN_59487 | MAOB | monoamine oxidase B | Cytoplasm | enzyme |
| 1 | down | ILMN_50869 | MBL2 | mannose-binding lectin (protein C) 2, soluble (opsonic defect) | Extracellular Space | other |
| 1 | down | ILMN_66321 | MBOAT5 | membrane bound O-acyltransferase domain containing 5 | Plasma Membrane | other |
| 1 | down | ILMN_66321 | MBOAT5 | membrane bound O-acyltransferase domain containing 5 | Plasma Membrane | other |
| 1 | down | ILMN_62535 | MCCC1 | methylcrotonoyl-Coenzyme A carboxylase 1 (alpha) | Cytoplasm | enzyme |
| 1 | down | ILMN_48356 | MCEE | methylmalonyl CoA epimerase | Cytoplasm | enzyme |
| 1 | down | ILMN_68069 | MDPdown | magnesium-dependent phosphatase 1 | Unknown | other |
| 1 | down | ILMN_59090 | ME3 | malic enzyme 3, NADP(+)-dependent, mitochondrial | Cytoplasm | enzyme |
| 1 | down | ILMN_48434 | MFN2 | mitofusin 2 | Cytoplasm | enzyme |
| 1 | down | ILMN_67343 | MGAT4B | mannosyl (alphadown,3-)-glycoprotein betadown,4-N-acetylglucosaminyltransferase, isozyme B | Unknown | enzyme |
| 1 | down | ILMN_48433 | MGLL | monoglyceride lipase | Plasma Membrane | enzyme |
| 1 | down | ILMN_47724 | MGST2 | microsomal glutathione S-transferase 2 | Cytoplasm | enzyme |
| 1 | down | ILMN_62452 | MID1IP1 | MID1 interacting protein 1 (gastrulation specific G12 homolog (zebrafish)) | Cytoplasm | other |
| 1 | down | ILMN_67717 | MIPEP | mitochondrial intermediate peptidase | Cytoplasm | peptidase |
| 1 | down | ILMN_56763 | MKNK2 | MAP kinase interacting serine/threonine kinase 2 | Cytoplasm | kinase |
| 1 | down | ILMN_69948 | MLC1 | megalencephalic leukoencephalopathy with subcortical cysts 1 | Plasma Membrane | transporter |
| 1 | down | ILMN_57624 | MLYCD | malonyl-CoA decarboxylase | Cytoplasm | enzyme |
| 1 | down | ILMN_52716 | MMP19 | matrix metallopeptidase 19 | Extracellular Space | peptidase |
| 1 | down | ILMN_50543 | MOCS2 | molybdenum cofactor synthesis 2 | Cytoplasm | enzyme |
| 1 | down | ILMN_60584 | MOSC2 | MOCO sulphurase C-terminal domain containing 2 | Cytoplasm | other |
| 1 | down | ILMN_64403 | MPPE1 | metallophosphoesterase 1 | Unknown | enzyme |
| 1 | down | ILMN_63600 | MRPL16 | mitochondrial ribosomal protein L16 | Unknown | other |
| 1 | down | ILMN_56709 | MRPL37 | mitochondrial ribosomal protein L37 | Cytoplasm | enzyme |
| 1 | down | ILMN_62801 | MSRB2 | methionine sulfoxide reductase B2 | Nucleus | transcription regulator |
| 1 | down | ILMN_55834 | MTCH2 | mitochondrial carrier homolog 2 (C. elegans) | Cytoplasm | other |
| 1 | down | ILMN_61386 | MTFMT | mitochondrial methionyl-tRNA formyltransferase | Cytoplasm | enzyme |
| 1 | down | ILMN_53784 | MTHFD1 | methylenetetrahydrofolate dehydrogenase (NADP+ dependent) 1, methenyltetrahydrofolate cyclohydrolase, formyltetrahydrofolate synthetase | Cytoplasm | enzyme |
| 1 | down | ILMN_51113 | MTHFS | 5,10-methenyltetrahydrofolate synthetase (5-formyltetrahydrofolate cyclo-ligase) | Cytoplasm | enzyme |
| 1 | down | ILMN_54430 | MTTP | microsomal triglyceride transfer protein | Cytoplasm | transporter |
| 1 | down | ILMN_48893 | NAE1 | NEDD8 activating enzyme E1 subunit 1 | Cytoplasm | enzyme |
| 1 | down | ILMN_65520 | NAGS | N-acetylglutamate synthase | Cytoplasm | enzyme |
| 1 | down | ILMN_67102 | NAPRT1 | nicotinate phosphoribosyltransferase domain containing 1 | Unknown | other |
| 1 | down | ILMN_64748 | NDRG2 | NDRG family member 2 | Cytoplasm | other |
| 1 | down | ILMN_51846 | NDST2 | N-deacetylase/N-sulfotransferase (heparan glucosaminyl) 2 | Cytoplasm | enzyme |
| 1 | down | ILMN_60239 | NDUFA8 | NADH dehydrogenase (ubiquinone) 1 alpha subcomplex, 8, 19kDa | Cytoplasm | enzyme |
| 1 | down | ILMN_64809 | NFIA | nuclear factor I/A | Nucleus | transcription regulator |
| 1 | down | ILMN_52904 | NIPSNAP1 | nipsnap homolog 1 (C. elegans) | Cytoplasm | enzyme |
| 1 | down | ILMN_52642 | NIPSNAP3A | nipsnap homolog 3A (C. elegans) | Cytoplasm | other |
| 1 | down | ILMN_59071 | NIT1 | nitrilase 1 | Unknown | enzyme |
| 1 | down | ILMN_47940 | NIT2 | nitrilase family, member 2 | Cytoplasm | enzyme |
| 1 | down | ILMN_54505 | NLN | neurolysin (metallopeptidase M3 family) | Cytoplasm | peptidase |
| 1 | down | ILMN_68868 | NLRP12 | NLR family, pyrin domain containing 12 | Cytoplasm | other |
| 1 | down | ILMN_53431 | NME3 | non-metastatic cells 3, protein expressed in | Extracellular Space | kinase |
| 1 | down | ILMN_67259 | NMRAL1 | NmrA-like family domain containing 1 | Unknown | other |
| 1 | down | ILMN_69238 | NNT | nicotinamide nucleotide transhydrogenase | Cytoplasm | enzyme |
| 1 | down | ILMN_68145 | NOTUM | notum pectinacetylesterase homolog (Drosophila) | Unknown | other |
| 1 | down | ILMN_52135 | NP | nucleoside phosphorylase | Nucleus | enzyme |
| 1 | down | ILMN_56919 | NR1H4 | nuclear receptor subfamily 1, group H, member 4 | Nucleus | ligand-dependent nuclear receptor |
| 1 | down | ILMN_50595 | NR1I2 | nuclear receptor subfamily 1, group I, member 2 | Nucleus | ligand-dependent nuclear receptor |
| 1 | down | ILMN_61940 | NR1I3 | nuclear receptor subfamily 1, group I, member 3 | Nucleus | ligand-dependent nuclear receptor |
| 1 | down | ILMN_66058 | NR2C1 (includes EG:7181) | nuclear receptor subfamily 2, group C, member 1 | Nucleus | transcription regulator |
| 1 | down | ILMN_65988 | NRBF2 | nuclear receptor binding factor 2 | Nucleus | transcription regulator |
| 1 | down | ILMN_52001 | NSDHL | NAD(P) dependent steroid dehydrogenase-like | Cytoplasm | enzyme |
| 1 | down | ILMN_58451 | NT5E | 5'-nucleotidase, ecto (CD73) | Plasma Membrane | phosphatase |
| 1 | down | ILMN_48712 | NUBPL | nucleotide binding protein-like | Extracellular Space | other |
| 1 | down | ILMN_56949 | NUDT14 | nudix (nucleoside diphosphate linked moiety X)-type motif 14 | Cytoplasm | phosphatase |
| 1 | down | ILMN_65374 | OPLAH | 5-oxoprolinase (ATP-hydrolysing) | Unknown | enzyme |
| 1 | down | ILMN_55721 | OPRS1 | opioid receptor, sigma 1 | Plasma Membrane | G-protein coupled receptor |
| 1 | down | ILMN_60044 | OR51E2 | olfactory receptor, family 51, subfamily E, member 2 | Plasma Membrane | G-protein coupled receptor |
| 1 | down | ILMN_60317 | OTC | ornithine carbamoyltransferase | Cytoplasm | enzyme |
| 1 | down | ILMN_66584 | PALMD | palmdelphin | Unknown | other |
| 1 | down | ILMN_53194 | PANK1 | pantothenate kinase 1 | Cytoplasm | kinase |
| 1 | down | ILMN_58548 | PC | pyruvate carboxylase | Cytoplasm | enzyme |
| 1 | down | ILMN_55727 | PCBD1 | pterin-4 alpha-carbinolamine dehydratase/dimerization cofactor of hepatocyte nuclear factor 1 alpha | Nucleus | transcription regulator |
| 1 | down | ILMN_57538 | PCTP | phosphatidylcholine transfer protein | Cytoplasm | transporter |
| 1 | down | ILMN_57723 | PCYOX1 | prenylcysteine oxidase 1 | Cytoplasm | enzyme |
| 1 | down | ILMN_47891 | PCYT2 | phosphate cytidylyltransferase 2, ethanolamine | Cytoplasm | enzyme |
| 1 | down | ILMN_64286 | PDCL3 | phosducin-like 3 | Cytoplasm | other |
| 1 | down | ILMN_47996 | PDK1 | pyruvate dehydrogenase kinase, isozyme 1 | Cytoplasm | kinase |
| 1 | down | ILMN_48078 | PDK2 | pyruvate dehydrogenase kinase, isozyme 2 | Cytoplasm | kinase |
| 1 | down | ILMN_58085 | PDXK | pyridoxal (pyridoxine, vitamin B6) kinase | Cytoplasm | kinase |
| 1 | down | ILMN_61185 | PDZK1 | PDZ domain containing 1 | Plasma Membrane | transporter |
| 1 | down | ILMN_68496 | PECI | peroxisomal D3,D2-enoyl-CoA isomerase | Cytoplasm | enzyme |
| 1 | down | ILMN_66523 | PEMT | phosphatidylethanolamine N-methyltransferase | Cytoplasm | enzyme |
| 1 | down | ILMN_63008 | PERP (includes EG:64065) | PERP, TP53 apoptosis effector | Plasma Membrane | other |
| 1 | down | ILMN_49567 | PEX14 | peroxisomal biogenesis factor 14 | Cytoplasm | other |
| 1 | down | ILMN_57378 | PEX16 | peroxisomal biogenesis factor 16 | Cytoplasm | other |
| 1 | down | ILMN_57378 | PEX16 | peroxisomal biogenesis factor 16 | Cytoplasm | other |
| 1 | down | ILMN_54092 | PEX5 | peroxisomal biogenesis factor 5 | Cytoplasm | transmembrane receptor |
| 1 | down | ILMN_67182 | PFKFB1 | 6-phosphofructo-2-kinase/fructose-2,6-biphosphatase 1 | Cytoplasm | kinase |
| 1 | down | ILMN_58520 | PGPEP1 | pyroglutamyl-peptidase I | Cytoplasm | peptidase |
| 1 | down | ILMN_55596 | PGRMC2 | progesterone receptor membrane component 2 | Nucleus | ligand-dependent nuclear receptor |
| 1 | down | ILMN_70065 | PHKB (includes EG:170631) | phosphorylase kinase, beta | Unknown | kinase |
| 1 | down | ILMN_63884 | PIGU | phosphatidylinositol glycan anchor biosynthesis, class U | Cytoplasm | enzyme |
| 1 | down | ILMN_56505 | PIPOX | pipecolic acid oxidase | Cytoplasm | enzyme |
| 1 | down | ILMN_51508 | PKIG | protein kinase (cAMP-dependent, catalytic) inhibitor gamma | Unknown | other |
| 1 | down | ILMN_56578 | PLD4 | phospholipase D family, member 4 | Unknown | enzyme |
| 1 | down | ILMN_65338 | PLS1 (includes EG:5357) | plastin 1 (I isoform) | Cytoplasm | other |
| 1 | down | ILMN_65634 | PLS3 | plastin 3 (T isoform) | Cytoplasm | other |
| 1 | down | ILMN_59085 | PMPCA | peptidase (mitochondrial processing) alpha | Cytoplasm | peptidase |
| 1 | down | ILMN_64054 | PMVK | phosphomevalonate kinase | Cytoplasm | kinase |
| 1 | down | ILMN_51005 | POLG2 | polymerase (DNA directed), gamma 2, accessory subunit | Cytoplasm | enzyme |
| 1 | down | ILMN_59207 | POLRMT | polymerase (RNA) mitochondrial (DNA directed) | Cytoplasm | enzyme |
| 1 | down | ILMN_60796 | PON2 | paraoxonase 2 | Extracellular Space | enzyme |
| 1 | down | ILMN_60269 | POP5 | processing of precursor 5, ribonuclease P/MRP subunit (S. cerevisiae) | Nucleus | enzyme |
| 1 | down | ILMN_51429 | PPA2 | pyrophosphatase (inorganic) 2 | Cytoplasm | enzyme |
| 1 | down | ILMN_53183 | PPIF | peptidylprolyl isomerase F (cyclophilin F) | Cytoplasm | enzyme |
| 1 | down | ILMN_48291 | PPP1R3B | protein phosphatase 1, regulatory (inhibitor) subunit 3B | Unknown | other |
| 1 | down | ILMN_53633 | PPP1R3C | protein phosphatase 1, regulatory (inhibitor) subunit 3C | Cytoplasm | phosphatase |
| 1 | down | ILMN_52584 | PRIM1 | primase, DNA, polypeptide 1 (49kDa) | Nucleus | enzyme |
| 1 | down | ILMN_50155 | PRKAG2 | protein kinase, AMP-activated, gamma 2 non-catalytic subunit | Unknown | kinase |
| 1 | down | ILMN_50018 | PRKD3 | protein kinase D3 | Unknown | kinase |
| 1 | down | ILMN_52471 | PRSS23 | protease, serine, 23 | Extracellular Space | peptidase |
| 1 | down | ILMN_48693 | PTDSS2 | phosphatidylserine synthase 2 | Cytoplasm | enzyme |
| 1 | down | ILMN_65996 | PTMS | parathymosin | Nucleus | other |
| 1 | down | ILMN_67728 | PXMP2 | peroxisomal membrane protein 2, 22kDa | Cytoplasm | other |
| 1 | down | ILMN_55375 | PYCRL | pyrroline-5-carboxylate reductase-like | Unknown | enzyme |
| 1 | down | ILMN_54065 | PYGL | phosphorylase, glycogen; liver (Hers disease, glycogen storage disease type VI) | Unknown | enzyme |
| 1 | down | ILMN_62820 | QDPR | quinoid dihydropteridine reductase | Cytoplasm | enzyme |
| 1 | down | ILMN_160789 | QPRT | quinolinate phosphoribosyltransferase (nicotinate-nucleotide pyrophosphorylase (carboxylating)) | Extracellular Space | enzyme |
| 1 | down | ILMN_62451 | QPRT | quinolinate phosphoribosyltransferase (nicotinate-nucleotide pyrophosphorylase (carboxylating)) | Extracellular Space | enzyme |
| 1 | down | ILMN_57446 | RARA | retinoic acid receptor, alpha | Nucleus | ligand-dependent nuclear receptor |
| 1 | down | ILMN_55755 | RARRES2 | retinoic acid receptor responder (tazarotene induced) 2 | Plasma Membrane | transmembrane receptor |
| 1 | down | ILMN_56855 | RBKS | ribokinase | Unknown | kinase |
| 1 | down | ILMN_59080 | RBL2 | retinoblastoma-like 2 (p130) | Nucleus | other |
| 1 | down | ILMN_62729 | RCBTB2 | regulator of chromosome condensation (RCC1) and BTB (POZ) domain containing protein 2 | Unknown | other |
| 1 | down | ILMN_60710 | RDH11 | retinol dehydrogenase 11 (all-trans/9-cis/11-cis) | Cytoplasm | enzyme |
| 1 | down | ILMN_55683 | RDX | radixin | Cytoplasm | other |
| 1 | down | ILMN_54215 | RFT1 | RFT1 homolog (S. cerevisiae) | Unknown | other |
| 1 | down | ILMN_49286 | RGD1308143 | similar to D330021B20 protein | Unknown | other |
| 1 | down | ILMN_65633 | RGD1310495 PREDICTED | similar to KIAA1919 protein (predicted) | Unknown | other |
| 1 | down | ILMN_51798 | RGD1559600 PREDICTED | RGD1559600 (predicted) | Unknown | other |
| 1 | down | ILMN_56008 | RGD1560220 PREDICTED | similar to homolog of yeast TIM14 isoform c (predicted) | Unknown | other |
| 1 | down | ILMN_63011 | RGD1560528 PREDICTED | similar to flt3 ligand (predicted) | Unknown | other |
| 1 | down | ILMN_49771 | RGD1560607 PREDICTED | similar to env precursor (predicted) | Unknown | other |
| 1 | down | ILMN_48540 | RGD1561002 PREDICTED | similar to concentrative Na+ nucleoside cotransporter (predicted) | Unknown | other |
| 1 | down | ILMN_67977 | RGD1561069 PREDICTED | similar to F-box only protein 31 (predicted) | Unknown | other |
| 1 | down | ILMN_56401 | RGD1561416 | similar to novel protein (HT036) (predicted) | Unknown | other |
| 1 | down | ILMN_60427 | RGD1561752 PREDICTED | RGD1561752 (predicted) | Unknown | other |
| 1 | down | ILMN_67262 | RGD1562272 PREDICTED | similar to TAF11 RNA polymerase II, TATA box binding protein (TBP)-associated factor (predicted) | Unknown | other |
| 1 | down | ILMN_56308 | RGD1563511 PREDICTED | similar to poly (ADP-ribose) polymerase family, member 10 (predicted) | Unknown | other |
| 1 | down | ILMN_53656 | RGD1563770 PREDICTED | similar to Lysozyme C, type 2 precursor (1,4-beta-N-acetylmuramidase C) (predicted) | Unknown | other |
| 1 | down | ILMN_54216 | RGD1563825 PREDICTED | similar to ENSANGP00000020885 (predicted) | Unknown | other |
| 1 | down | ILMN_67878 | RGD1564865 PREDICTED | similar to 20-alpha-hydroxysteroid dehydrogenase (predicted) | Unknown | other |
| 1 | down | ILMN_50229 | RGD1564881 PREDICTED | similar to U2af1-rs2 (predicted) | Unknown | other |
| 1 | down | ILMN_65454 | RGD1564910 PREDICTED | similar to RIKEN cDNA 8430426H19 (predicted) | Unknown | other |
| 1 | down | ILMN_64546 | RGD1565110 PREDICTED | RGD1565110 (predicted) | Unknown | other |
| 1 | down | ILMN_59111 | RGD1565605 PREDICTED | similar to novel zinc finger protein (predicted) | Unknown | other |
| 1 | down | ILMN_59712 | RGD1565988 | similar to Chain A, Bhmt From Rat Liver (predicted) | Unknown | other |
| 1 | down | ILMN_49577 | RGD1566307 PREDICTED | similar to PIRB1 (predicted) | Unknown | other |
| 1 | down | ILMN_50843 | RGN | regucalcin (senescence marker protein-30) | Nucleus | other |
| 1 | down | ILMN_54423 | RHOD | ras homolog gene family, member D | Cytoplasm | enzyme |
| 1 | down | ILMN_66191 | RNASE4 | ribonuclease, RNase A family, 4 | Extracellular Space | enzyme |
| 1 | down | ILMN_60154 | RNF103 | ring finger protein 103 | Unknown | other |
| 1 | down | ILMN_64344 | RNF141 | ring finger protein 141 | Unknown | other |
| 1 | down | ILMN_54783 | RNPEP | arginyl aminopeptidase (aminopeptidase B) | Cytoplasm | peptidase |
| 1 | down | ILMN_70214 | RP3-402G11.5 | selenoprotein O | Extracellular Space | enzyme |
| 1 | down | ILMN_54485 | RPP21 | ribonuclease P/MRP 21kDa subunit | Nucleus | enzyme |
| 1 | down | ILMN_54197 | RTP3 | receptor (chemosensory) transporter protein 3 | Plasma Membrane | other |
| 1 | down | ILMN_66946 | RWDD3 | RWD domain containing 3 | Unknown | other |
| 1 | down | ILMN_64209 | RXRG | retinoid X receptor, gamma | Nucleus | ligand-dependent nuclear receptor |
| 1 | down | ILMN_52607 | SAPS3 | SAPS domain family, member 3 | Unknown | other |
| 1 | down | ILMN_56716 | SARDH | sarcosine dehydrogenase | Cytoplasm | enzyme |
| 1 | down | ILMN_56545 | SC5DL | sterol-C5-desaturase (ERG3 delta-5-desaturase homolog, S. cerevisiae)-like | Cytoplasm | enzyme |
| 1 | down | ILMN_64154 | SCARB1 | scavenger receptor class B, member 1 | Plasma Membrane | transporter |
| 1 | down | ILMN_51842 | SCCPDH | saccharopine dehydrogenase (putative) | Cytoplasm | other |
| 1 | down | ILMN_54294 | SCRN2 | secernin 2 | Unknown | other |
| 1 | down | ILMN_53433 | SDHC | succinate dehydrogenase complex, subunit C, integral membrane protein, 15kDa | Cytoplasm | enzyme |
| 1 | down | ILMN_63781 | SDHD | succinate dehydrogenase complex, subunit D, integral membrane protein | Cytoplasm | enzyme |
| 1 | down | ILMN_56509 | SEC22A | SEC22 vesicle trafficking protein homolog A (S. cerevisiae) | Cytoplasm | transporter |
| 1 | down | ILMN_48251 | SECTM1B | secreted and transmembrane 1B | Extracellular Space | cytokine |
| 1 | down | ILMN_59759 | SELENBP1 | selenium binding protein 1 | Cytoplasm | other |
| 1 | down | ILMN_61414 | SEMA4G | sema domain, immunoglobulin domain (Ig), transmembrane domain (TM) and short cytoplasmic domain, (semaphorin) 4G | Plasma Membrane | other |
| 1 | down | ILMN_65813 | SERPIND1 | serpin peptidase inhibitor, clade D (heparin cofactor), member 1 | Extracellular Space | other |
| 1 | down | ILMN_52975 | SETD3 | SET domain containing 3 | Unknown | other |
| 1 | down | ILMN_56677 | SEZ6 | seizure related 6 homolog (mouse) | Unknown | other |
| 1 | down | ILMN_52738 | SF4 | splicing factor 4 | Nucleus | other |
| 1 | down | ILMN_55044 | SFRS12IP1 | SFRS12-interacting protein 1 | Unknown | other |
| 1 | down | ILMN_51573 | SFXN1 | sideroflexin 1 | Cytoplasm | transporter |
| 1 | down | ILMN_66383 | SFXN5 | sideroflexin 5 | Cytoplasm | transporter |
| 1 | down | ILMN_53614 | SIRT5 | sirtuin (silent mating type information regulation 2 homolog) 5 (S. cerevisiae) | Cytoplasm | enzyme |
| 1 | down | ILMN_53103 | SLC10A1 | solute carrier family 10 (sodium/bile acid cotransporter family), member 1 | Plasma Membrane | transporter |
| 1 | down | ILMN_65197 | SLC19A1 | solute carrier family 19 (folate transporter), member 1 | Plasma Membrane | transporter |
| 1 | down | ILMN_65027 | SLC22A18 | solute carrier family 22 (organic cation transporter), member 18 | Plasma Membrane | transporter |
| 1 | down | ILMN_63974 | SLC23A1 | solute carrier family 23 (nucleobase transporters), member 1 | Plasma Membrane | transporter |
| 1 | down | ILMN_66843 | SLC25A1 | solute carrier family 25 (mitochondrial carrier; citrate transporter), member 1 | Plasma Membrane | transporter |
| 1 | down | ILMN_66823 | SLC25A11 | solute carrier family 25 (mitochondrial carrier; oxoglutarate carrier), member 11 | Cytoplasm | transporter |
| 1 | down | ILMN_61069 | SLC25A13 | solute carrier family 25, member 13 (citrin) | Cytoplasm | transporter |
| 1 | down | ILMN_65032 | SLC26A1 | solute carrier family 26 (sulfate transporter), member 1 | Plasma Membrane | transporter |
| 1 | down | ILMN_53356 | SLC27A1 | solute carrier family 27 (fatty acid transporter), member 1 | Plasma Membrane | transporter |
| 1 | down | ILMN_56822 | SLC2A2 | solute carrier family 2 (facilitated glucose transporter), member 2 | Plasma Membrane | transporter |
| 1 | down | ILMN_56998 | SLC31A1 | solute carrier family 31 (copper transporters), member 1 | Plasma Membrane | transporter |
| 1 | down | ILMN_54840 | SLC34A2 | solute carrier family 34 (sodium phosphate), member 2 | Plasma Membrane | transporter |
| 1 | down | ILMN_51888 | SLC37A4 | solute carrier family 37 (glucose-6-phosphate transporter), member 4 | Cytoplasm | transporter |
| 1 | down | ILMN_52521 | SLC39A8 | solute carrier family 39 (zinc transporter), member 8 | Extracellular Space | transporter |
| 1 | down | ILMN_48348 | SLCO1B3 | solute carrier organic anion transporter family, member 1B3 | Plasma Membrane | transporter |
| 1 | down | ILMN_60569 | SLCO2A1 | solute carrier organic anion transporter family, member 2A1 | Plasma Membrane | transporter |
| 1 | down | ILMN_64055 | SLCO2B1 | solute carrier organic anion transporter family, member 2B1 | Plasma Membrane | transporter |
| 1 | down | ILMN_51316 | SMP2A | rat senescence marker protein 2A gene, exons 1 and 2 | Cytoplasm | enzyme |
| 1 | down | ILMN_68528 | SNTA1 | syntrophin, alpha 1 (dystrophin-associated protein A1, 59kDa, acidic component) | Plasma Membrane | other |
| 1 | down | ILMN_57551 | SORD | sorbitol dehydrogenase | Unknown | enzyme |
| 1 | down | ILMN_67122 | SPTBN2 | spectrin, beta, non-erythrocytic 2 | Cytoplasm | other |
| 1 | down | ILMN_51834 | SREBF1 | sterol regulatory element binding transcription factor 1 | Nucleus | transcription regulator |
| 1 | down | ILMN_62464 | SRR | serine racemase | Cytoplasm | enzyme |
| 1 | down | ILMN_161750 | ST5 | suppression of tumorigenicity 5 | Unknown | enzyme |
| 1 | down | ILMN_61174 | STAC3 | SH3 and cysteine rich domain 3 | Unknown | other |
| 1 | down | ILMN_54264 | STARD7 | StAR-related lipid transfer (START) domain containing 7 | Unknown | other |
| 1 | down | ILMN_59306 | SUDS3 | suppressor of defective silencing 3 homolog (S. cerevisiae) | Nucleus | other |
| 1 | down | ILMN_52849 | SULT1A1 | sulfotransferase family, cytosolic, 1A, phenol-preferring, member 1 | Cytoplasm | enzyme |
| 1 | down | ILMN_64688 | SULT1B1 | sulfotransferase family, cytosolic, 1B, member 1 | Cytoplasm | enzyme |
| 1 | down | ILMN_55734 | SULT1E1 | sulfotransferase family 1E, estrogen-preferring, member 1 | Cytoplasm | enzyme |
| 1 | down | ILMN_48036 | SUMF2 | sulfatase modifying factor 2 | Cytoplasm | other |
| 1 | down | ILMN_53970 | SUOX | sulfite oxidase | Cytoplasm | enzyme |
| 1 | down | ILMN_61732 | SVIL | supervillin | Plasma Membrane | other |
| 1 | down | ILMN_60662 | TAF11 | TAF11 RNA polymerase II, TATA box binding protein (TBP)-associated factor, 28kDa | Nucleus | transcription regulator |
| 1 | down | ILMN_69115 | TAZ | tafazzin (cardiomyopathy, dilated 3A (X-linked); endocardial fibroelastosis 2; Barth syndrome) | Nucleus | enzyme |
| 1 | down | ILMN_61017 | TBCE | tubulin folding cofactor E | Cytoplasm | other |
| 1 | down | ILMN_52949 | TCIRG1 | T-cell, immune regulator 1, ATPase, H+ transporting, lysosomal V0 subunit A3 | Plasma Membrane | enzyme |
| 1 | down | ILMN_68916 | TEC | tec protein tyrosine kinase | Cytoplasm | kinase |
| 1 | down | ILMN_52467 | TEX9 | testis expressed 9 | Unknown | other |
| 1 | down | ILMN_65512 | TFDP2 | transcription factor Dp-2 (E2F dimerization partner 2) | Nucleus | transcription regulator |
| 1 | down | ILMN_52836 | THNSL2 | threonine synthase-like 2 (S. cerevisiae) | Unknown | other |
| 1 | down | ILMN_50593 | THRSP | thyroid hormone responsive (SPOT14 homolog, rat) | Nucleus | other |
| 1 | down | ILMN_70333 | THUMPD1 | THUMP domain containing 1 | Unknown | other |
| 1 | down | ILMN_64633 | TMEM123 | transmembrane protein 123 | Plasma Membrane | other |
| 1 | down | ILMN_59782 | TMEM126B | transmembrane protein 126B | Unknown | other |
| 1 | down | ILMN_56730 | TMEM140 | transmembrane protein 140 | Unknown | other |
| 1 | down | ILMN_69928 | TMEM141 | transmembrane protein 141 | Unknown | other |
| 1 | down | ILMN_56929 | TMEM161B | transmembrane protein 161B | Unknown | other |
| 1 | down | ILMN_57856 | TMEM16K | transmembrane protein 16K | Unknown | other |
| 1 | down | ILMN_68686 | TMEM19 | transmembrane protein 19 | Unknown | other |
| 1 | down | ILMN_60225 | TMEM37 | transmembrane protein 37 | Plasma Membrane | ion channel |
| 1 | down | ILMN_54006 | TMEM53 | transmembrane protein 53 | Unknown | other |
| 1 | down | ILMN_64744 | TMEM9 | transmembrane protein 9 | Cytoplasm | other |
| 1 | down | ILMN_51305 | TMLHE | trimethyllysine hydroxylase, epsilon | Cytoplasm | enzyme |
| 1 | down | ILMN_67961 | TOM1L1 | target of myb1 (chicken)-like 1 | Cytoplasm | other |
| 1 | down | ILMN_51591 | TPCN1 | two pore segment channel 1 | Plasma Membrane | ion channel |
| 1 | down | ILMN_60762 | TRAP1 | TNF receptor-associated protein 1 | Cytoplasm | enzyme |
| 1 | down | ILMN_55189 | TRIM14 | tripartite motif-containing 14 | Cytoplasm | other |
| 1 | down | ILMN_68183 | TRIM6 | tripartite motif-containing 6 | Unknown | other |
| 1 | down | ILMN_68874 | TRUB1 | TruB pseudouridine (psi) synthase homolog 1 (E. coli) | Unknown | enzyme |
| 1 | down | ILMN_60864 | TSC22D3 | TSC22 domain family, member 3 | Nucleus | transcription regulator |
| 1 | down | ILMN_62812 | TSPAN31 | tetraspanin 31 | Plasma Membrane | other |
| 1 | down | ILMN_60491 | TST | thiosulfate sulfurtransferase (rhodanese) | Cytoplasm | enzyme |
| 1 | down | ILMN_50167 | TTC23 | tetratricopeptide repeat domain 23 | Unknown | other |
| 1 | down | ILMN_62555 | TTPA | tocopherol (alpha) transfer protein | Cytoplasm | transporter |
| 1 | down | ILMN_62515 | UGT2B10 | UDP glucuronosyltransferase 2 family, polypeptide B10 | Cytoplasm | enzyme |
| 1 | down | ILMN_64654 | UGT2B4 | UDP glucuronosyltransferase 2 family, polypeptide B4 | Cytoplasm | enzyme |
| 1 | down | ILMN_57260 | UGT2B5 | UDP glucuronosyltransferase 2 family, polypeptide B5 | Cytoplasm | enzyme |
| 1 | down | ILMN_70352 | UGT2B5 | UDP glucuronosyltransferase 2 family, polypeptide B5 | Cytoplasm | enzyme |
| 1 | down | ILMN_59649 | UGT2B7 | UDP glucuronosyltransferase 2 family, polypeptide B7 | Cytoplasm | enzyme |
| 1 | down | ILMN_69416 | UNQ1940 | HWKM1940 | Unknown | other |
| 1 | down | ILMN_68253 | UPB1 | ureidopropionase, beta | Cytoplasm | enzyme |
| 1 | down | ILMN_53650 | UROD | uroporphyrinogen decarboxylase | Cytoplasm | enzyme |
| 1 | down | ILMN_65908 | USP2 | ubiquitin specific peptidase 2 | Cytoplasm | peptidase |
| 1 | down | ILMN_60127 | VDAC1 | voltage-dependent anion channel 1 | Cytoplasm | ion channel |
| 1 | down | ILMN_60106 | VPS41 | vacuolar protein sorting 41 homolog (S. cerevisiae) | Cytoplasm | transporter |
| 1 | down | ILMN_53759 | WDR23 | WD repeat domain 23 | Unknown | other |
| 1 | down | ILMN_57753 | WFDC3 | WAP four-disulfide core domain 3 | Extracellular Space | other |
| 1 | down | ILMN_60158 | WWP1 | WW domain containing E3 ubiquitin protein ligase 1 | Cytoplasm | enzyme |
| 1 | down | ILMN_62821 | XPNPEP2 | X-prolyl aminopeptidase (aminopeptidase P) 2, membrane-bound | Plasma Membrane | peptidase |
| 1 | down | ILMN_54498 | XRCC1 | X-ray repair complementing defective repair in Chinese hamster cells 1 | Nucleus | other |
| 1 | down | ILMN_61262 | XRCC6BP1 | XRCC6 binding protein 1 | Unknown | kinase |
| 1 | down | ILMN_66933 | YIPF1 | Yip1 domain family, member 1 | Unknown | other |
| 1 | down | ILMN_59698 | ZFHX4 | zinc finger homeobox 4 | Unknown | other |
| 1 | down | ILMN_51230 | ZNF324 | zinc finger protein 324 | Nucleus | other |
| 3 | up | ILMN_63134 | 1600029D21RIK | RIKEN cDNA 1600029D21 gene | Unknown | other |
| 3 | up | ILMN_49518 | 2700097O09RIK | RIKEN cDNA 2700097O09 gene | Extracellular Space | other |
| 3 | up | ILMN_62752 | AADACL1 | arylacetamide deacetylase-like 1 | Plasma Membrane | enzyme |
| 3 | up | ILMN_63617 | ABCB1B | ATP-binding cassette, sub-family B (MDR/TAP), member 1B | Plasma Membrane | transporter |
| 3 | up | ILMN_63540 | ADAMTS9 | ADAM metallopeptidase with thrombospondin type 1 motif, 9 | Extracellular Space | peptidase |
| 3 | up | ILMN_58181 | ADORA2A | adenosine A2a receptor | Plasma Membrane | G-protein coupled receptor |
| 3 | up | ILMN_60014 | AHSA2 (includes EG:268390) | AHA1, activator of heat shock protein ATPase homolog 2 (yeast) | Unknown | other |
| 3 | up | ILMN_68240 | AKTIP | AKT interacting protein | Cytoplasm | other |
| 3 | up | ILMN_55629 | ALDH18A1 | aldehyde dehydrogenase 18 family, member A1 | Cytoplasm | kinase |
| 3 | up | ILMN_67819 | ALDOA | aldolase A, fructose-bisphosphate | Cytoplasm | enzyme |
| 3 | up | ILMN_67819 | ALDOA | aldolase A, fructose-bisphosphate | Cytoplasm | enzyme |
| 3 | up | ILMN_66142 | ALG1 | asparagine-linked glycosylation 1 homolog (S. cerevisiae, betadown,4-mannosyltransferase) | Cytoplasm | enzyme |
| 3 | up | ILMN_52386 | ALKBH5 | alkB, alkylation repair homolog 5 (E. coli) | Unknown | other |
| 3 | up | ILMN_68167 | ANGPTL4 | angiopoietin-like 4 | Extracellular Space | other |
| 3 | up | ILMN_51664 | ANKRD37 | ankyrin repeat domain 37 | Unknown | other |
| 3 | up | ILMN_56061 | AP2B1 | adaptor-related protein complex 2, beta 1 subunit | Cytoplasm | transporter |
| 3 | up | ILMN_54329 | AP2M1 | adaptor-related protein complex 2, mu 1 subunit | Cytoplasm | transporter |
| 3 | up | ILMN_58888 | AP3M1 | adaptor-related protein complex 3, mu 1 subunit | Cytoplasm | transporter |
| 3 | up | ILMN_60393 | ARFIP2 | ADP-ribosylation factor interacting protein 2 (arfaptin 2) | Cytoplasm | other |
| 3 | up | ILMN_69268 | ARHGDIA | Rho GDP dissociation inhibitor (GDI) alpha | Cytoplasm | other |
| 3 | up | ILMN_61891 | ARID5A | AT rich interactive domain 5A (MRF1-like) | Nucleus | transcription regulator |
| 3 | up | ILMN_63568 | ARL8A | ADP-ribosylation factor-like 8A | Cytoplasm | enzyme |
| 3 | up | ILMN_53383 | ARMC10 | armadillo repeat containing 10 | Cytoplasm | other |
| 3 | up | ILMN_70217 | ARMET | arginine-rich, mutated in early stage tumors | Extracellular Space | other |
| 3 | up | ILMN_49151 | ASNS | asparagine synthetase | Unknown | enzyme |
| 3 | up | ILMN_58762 | ATF7 | activating transcription factor 7 | Nucleus | transcription regulator |
| 3 | up | ILMN_53801 | ATP6V0C | ATPase, H+ transporting, lysosomal 16kDa, V0 subunit c | Cytoplasm | transporter |
| 3 | up | ILMN_57022 | ATP6V1G2 | ATPase, H+ transporting, lysosomal 13kDa, V1 subunit G2 | Cytoplasm | transporter |
| 3 | up | ILMN_59393 | B3GNT2 | UDP-GlcNAc:betaGal betadown,3-N-acetylglucosaminyltransferase 2 | Cytoplasm | enzyme |
| 3 | up | ILMN_64889 | B4GALT1 | UDP-Gal:betaGlcNAc beta 1,4- galactosyltransferase, polypeptide 1 | Cytoplasm | enzyme |
| 3 | up | ILMN_53804 | B4GALT3 | UDP-Gal:betaGlcNAc beta 1,4- galactosyltransferase, polypeptide 3 | Cytoplasm | enzyme |
| 3 | up | ILMN_68489 | BANP | BTG3 associated nuclear protein | Nucleus | other |
| 3 | up | ILMN_68430 | BASP1 | brain abundant, membrane attached signal protein 1 | Plasma Membrane | other |
| 3 | up | ILMN_64237 | BCL10 | B-cell CLL/lymphoma 10 | Cytoplasm | transcription regulator |
| 3 | up | ILMN_70415 | BCR | breakpoint cluster region | Cytoplasm | kinase |
| 3 | up | ILMN_63976 | BHLHB2 | basic helix-loop-helix domain containing, class B, 2 | Nucleus | transcription regulator |
| 3 | up | ILMN_48546 | BHLHB3 | basic helix-loop-helix domain containing, class B, 3 | Nucleus | transcription regulator |
| 3 | up | ILMN_57133 | BOP1 | block of proliferation 1 | Nucleus | other |
| 3 | up | ILMN_61214 | BRD2 | bromodomain containing 2 | Nucleus | kinase |
| 3 | up | ILMN_49580 | BTBD10 | BTB (POZ) domain containing 10 | Unknown | ion channel |
| 3 | up | ILMN_54558 | BTG3 | BTG family, member 3 | Nucleus | other |
| 3 | up | ILMN_68789 | C11ORF47 | chromosome 11 open reading frame 47 | Unknown | other |
| 3 | up | ILMN_57720 | C12ORF41 | chromosome 12 open reading frame 41 | Unknown | other |
| 3 | up | ILMN_48956 | C14ORF102 | chromosome 14 open reading frame 102 | Unknown | other |
| 3 | up | ILMN_57070 | C18ORF51 | chromosome 18 open reading frame 51 | Unknown | other |
| 3 | up | ILMN_61296 | C3ORF17 | chromosome 3 open reading frame 17 | Unknown | other |
| 3 | up | ILMN_69848 | C4ORF16 | chromosome 4 open reading frame 16 | Unknown | other |
| 3 | up | ILMN_49366 | C5ORF30 | chromosome 5 open reading frame 30 | Unknown | other |
| 3 | up | ILMN_68131 | C8ORF53 | chromosome 8 open reading frame 53 | Unknown | other |
| 3 | up | ILMN_59018 | C9ORF150 | chromosome 9 open reading frame 150 | Unknown | other |
| 3 | up | ILMN_59713 | C9ORF52 | chromosome 9 open reading frame 52 | Unknown | other |
| 3 | up | ILMN_65242 | CALM1 | calmodulin 1 (phosphorylase kinase, delta) | Plasma Membrane | other |
| 3 | up | ILMN_50498 | CASP3 | caspase 3, apoptosis-related cysteine peptidase | Cytoplasm | peptidase |
| 3 | up | ILMN_52357 | CCBL1 | cysteine conjugate-beta lyase; cytoplasmic (glutamine transaminase K, kyneurenine aminotransferase) | Cytoplasm | enzyme |
| 3 | up | ILMN_57505 | CCDC116 | coiled-coil domain containing 116 | Extracellular Space | other |
| 3 | up | ILMN_69718 | CCNL1 | cyclin L1 | Nucleus | other |
| 3 | up | ILMN_60446 | CDC42EP1 | CDC42 effector protein (Rho GTPase binding) 1 | Extracellular Space | other |
| 3 | up | ILMN_61409 | CEACAM16 | carcinoembryonic antigen-related cell adhesion molecule 16 | Unknown | transcription regulator |
| 3 | up | ILMN_60309 | CEBPB | CCAAT/enhancer binding protein (C/EBP), beta | Nucleus | transcription regulator |
| 3 | up | ILMN_62816 | CFLAR | CASP8 and FADD-like apoptosis regulator | Cytoplasm | other |
| 3 | up | ILMN_58442 | CGREF1 | cell growth regulator with EF-hand domain 1 | Extracellular Space | other |
| 3 | up | ILMN_52950 | CH25H | cholesterol 25-hydroxylase | Cytoplasm | enzyme |
| 3 | up | ILMN_67315 | CHAC1 | ChaC, cation transport regulator homolog 1 (E. coli) | Unknown | other |
| 3 | up | ILMN_62763 | CHCHD4 | coiled-coil-helix-coiled-coil-helix domain containing 4 | Unknown | other |
| 3 | up | ILMN_69178 | CHPF | chondroitin polymerizing factor | Cytoplasm | enzyme |
| 3 | up | ILMN_60384 | CHSY-2 | chondroitin synthase-2 | Cytoplasm | enzyme |
| 3 | up | ILMN_63252 | CIRH1A | cirrhosis, autosomal recessive 1A (cirhin) | Nucleus | other |
| 3 | up | ILMN_52094 | CISD2 | CDGSH iron sulfur domain 2 | Unknown | other |
| 3 | up | ILMN_53884 | CLTB | clathrin, light chain (Lcb) | Plasma Membrane | other |
| 3 | up | ILMN_55700 | COL11A2 | collagen, type XI, alpha 2 | Extracellular Space | other |
| 3 | up | ILMN_62363 | COQ10B | coenzyme Q10 homolog B (S. cerevisiae) | Unknown | other |
| 3 | up | ILMN_69682 | CREB3L2 | cAMP responsive element binding protein 3-like 2 | Unknown | other |
| 3 | up | ILMN_49348 | CRELD1 | cysteine-rich with EGF-like domains 1 | Unknown | other |
| 3 | up | ILMN_65741 | CXCL3 | chemokine (C-X-C motif) ligand 3 | Extracellular Space | cytokine |
| 3 | up | ILMN_66174 | CXORF26 | chromosome X open reading frame 26 | Unknown | other |
| 3 | up | ILMN_49849 | CYP1B1 | cytochrome P450, family 1, subfamily B, polypeptide 1 | Cytoplasm | enzyme |
| 3 | up | ILMN_52124 | CYP4F16 | cytochrome P450, family 4, subfamily f, polypeptide 16 | Cytoplasm | enzyme |
| 3 | up | ILMN_64337 | DCTN4 | dynactin 4 (p62) | Nucleus | other |
| 3 | up | ILMN_69830 | DDIT3 | DNA-damage-inducible transcript 3 | Nucleus | transcription regulator |
| 3 | up | ILMN_49640 | DDIT4 | DNA-damage-inducible transcript 4 | Cytoplasm | other |
| 3 | up | ILMN_53214 | DDOST (includes EG:1650) | dolichyl-diphosphooligosaccharide-protein glycosyltransferase | Cytoplasm | enzyme |
| 3 | up | ILMN_54128 | DDX56 | DEAD (Asp-Glu-Ala-Asp) box polypeptide 56 | Nucleus | enzyme |
| 3 | up | ILMN_61313 | DES | desmin | Cytoplasm | other |
| 3 | up | ILMN_61057 | DMBT1 | deleted in malignant brain tumors 1 | Plasma Membrane | transmembrane receptor |
| 3 | up | ILMN_66692 | DNAJB5 | DnaJ (Hsp40) homolog, subfamily B, member 5 | Unknown | other |
| 3 | up | ILMN_59747 | DSC2 | desmocollin 2 | Plasma Membrane | other |
| 3 | up | ILMN_65350 | DTX2 | deltex homolog 2 (Drosophila) | Nucleus | other |
| 3 | up | ILMN_50424 | DUSP8 | dual specificity phosphatase 8 | Nucleus | phosphatase |
| 3 | up | ILMN_56473 | E2F5 | E2F transcription factor 5, p130-binding | Nucleus | transcription regulator |
| 3 | up | ILMN_61661 | EDG5 | endothelial differentiation, sphingolipid G-protein-coupled receptor, 5 | Plasma Membrane | G-protein coupled receptor |
| 3 | up | ILMN_67596 | EFEMP2 | EGF-containing fibulin-like extracellular matrix protein 2 | Extracellular Space | other |
| 3 | up | ILMN_54672 | EIF1AD | eukaryotic translation initiation factor 1A domain containing | Unknown | other |
| 3 | up | ILMN_64232 | EIF2C2 | eukaryotic translation initiation factor 2C, 2 | Cytoplasm | translation regulator |
| 3 | up | ILMN_62651 | EIF4EBP1 | eukaryotic translation initiation factor 4E binding protein 1 | Cytoplasm | translation regulator |
| 3 | up | ILMN_63845 | ELAVL1 | ELAV (embryonic lethal, abnormal vision, Drosophila)-like 1 (Hu antigen R) | Cytoplasm | other |
| 3 | up | ILMN_66456 | ELTD1 | EGF, latrophilin and seven transmembrane domain containing 1 | Plasma Membrane | G-protein coupled receptor |
| 3 | up | ILMN_59863 | EMD | emerin (Emery-Dreifuss muscular dystrophy) | Nucleus | other |
| 3 | up | ILMN_48153 | ENOPH1 | enolase-phosphatase 1 | Cytoplasm | enzyme |
| 3 | up | ILMN_59510 | EPAS1 | endothelial PAS domain protein 1 | Nucleus | transcription regulator |
| 3 | up | ILMN_54237 | ESAM | endothelial cell adhesion molecule | Plasma Membrane | other |
| 3 | up | ILMN_48844 | FAM3C | family with sequence similarity 3, member C | Extracellular Space | cytokine |
| 3 | up | ILMN_48965 | FBLIM1 | filamin binding LIM protein 1 | Plasma Membrane | other |
| 3 | up | ILMN_48958 | FGD6 | FYVE, RhoGEF and PH domain containing 6 | Cytoplasm | other |
| 3 | up | ILMN_55402 | FLJ11184 | hypothetical protein FLJ11184 | Unknown | other |
| 3 | up | ILMN_66963 | FLJ36031 | hypothetical protein FLJ36031 | Unknown | other |
| 3 | up | ILMN_56287 | FMO5 | flavin containing monooxygenase 5 | Cytoplasm | enzyme |
| 3 | up | ILMN_62708 | FNTA | farnesyltransferase, CAAX box, alpha | Cytoplasm | enzyme |
| 3 | up | ILMN_68654 | FOXK2 | forkhead box K2 | Nucleus | transcription regulator |
| 3 | up | ILMN_62183 | GADD45B | growth arrest and DNA-damage-inducible, beta | Cytoplasm | other |
| 3 | up | ILMN_58213 | GEM | GTP binding protein overexpressed in skeletal muscle | Plasma Membrane | enzyme |
| 3 | up | ILMN_51223 | GFPT1 | glutamine-fructose-6-phosphate transaminase 1 | Cytoplasm | enzyme |
| 3 | up | ILMN_54462 | GFPT2 | glutamine-fructose-6-phosphate transaminase 2 | Unknown | enzyme |
| 3 | up | ILMN_58858 | GGA3 | golgi associated, gamma adaptin ear containing, ARF binding protein 3 | Cytoplasm | transporter |
| 3 | up | ILMN_51511 | GJA1 | gap junction protein, alpha 1, 43kDa | Plasma Membrane | transporter |
| 3 | up | ILMN_58980 | GM2A | GM2 ganglioside activator | Cytoplasm | enzyme |
| 3 | up | ILMN_62583 | GOLT1B | golgi transport 1 homolog B (S. cerevisiae) | Cytoplasm | other |
| 3 | up | ILMN_63702 | GPR109A | G protein-coupled receptor 109A | Plasma Membrane | G-protein coupled receptor |
| 3 | up | ILMN_60501 | GPR4 | G protein-coupled receptor 4 | Plasma Membrane | G-protein coupled receptor |
| 3 | up | ILMN_67432 | GTF2IRD1 | GTF2I repeat domain containing 1 | Nucleus | transcription regulator |
| 3 | up | ILMN_48721 | GTPBP2 | GTP binding protein 2 | Unknown | enzyme |
| 3 | up | ILMN_57876 | HDGFRP3 | hepatoma-derived growth factor, related protein 3 | Nucleus | other |
| 3 | up | ILMN_63478 | HEATR6 | HEAT repeat containing 6 | Unknown | other |
| 3 | up | ILMN_51127 | HERPUD1 | homocysteine-inducible, endoplasmic reticulum stress-inducible, ubiquitin-like domain member 1 | Cytoplasm | other |
| 3 | up | ILMN_64022 | HIF1A | hypoxia-inducible factor 1, alpha subunit (basic helix-loop-helix transcription factor) | Nucleus | transcription regulator |
| 3 | up | ILMN_58516 | HIST1H2AL (includes EG:8332) | histone cluster 1, H2al | Nucleus | other |
| 3 | up | ILMN_52486 | HIVEP1 | human immunodeficiency virus type I enhancer binding protein 1 | Nucleus | transcription regulator |
| 3 | up | ILMN_61530 | HS6ST1 | heparan sulfate 6-O-sulfotransferase 1 | Extracellular Space | enzyme |
| 3 | up | ILMN_56818 | HSPBP1 | hsp70-interacting protein | Unknown | other |
| 3 | up | ILMN_59429 | HSPC152 | hypothetical protein HSPC152 | Unknown | other |
| 3 | up | ILMN_66194 | HTATIP | HIVdown Tat interacting protein, 60kDa | Nucleus | transcription regulator |
| 3 | up | ILMN_61139 | ICAM1 | intercellular adhesion molecule 1 (CD54), human rhinovirus receptor | Plasma Membrane | transmembrane receptor |
| 3 | up | ILMN_48292 | IFRD2 | interferon-related developmental regulator 2 | Unknown | other |
| 3 | up | ILMN_59914 | IL10RB | interleukin 10 receptor, beta | Plasma Membrane | transmembrane receptor |
| 3 | up | ILMN_49738 | IL1R2 | interleukin 1 receptor, type II | Plasma Membrane | transmembrane receptor |
| 3 | up | ILMN_50105 | IL1RN | interleukin 1 receptor antagonist | Extracellular Space | cytokine |
| 3 | up | ILMN_67252 | IMP3 | IMP3, U3 small nucleolar ribonucleoprotein, homolog (yeast) | Cytoplasm | other |
| 3 | up | ILMN_57038 | IRAK3 | interleukindown receptor-associated kinase 3 | Extracellular Space | kinase |
| 3 | up | ILMN_52177 | ISG20L1 | interferon stimulated exonuclease gene 20kDa-like 1 | Unknown | other |
| 3 | up | ILMN_59284 | ISY1 | ISY1 splicing factor homolog (S. cerevisiae) | Nucleus | other |
| 3 | up | ILMN_50826 | ISYNA1 | myo-inositol 1-phosphate synthase A1 | Unknown | enzyme |
| 3 | up | ILMN_63152 | ITGB1 | integrin, beta 1 (fibronectin receptor, beta polypeptide, antigen CD29 includes MDF2, MSK12) | Plasma Membrane | transmembrane receptor |
| 3 | up | ILMN_61930 | ITPKC | inositol 1,4,5-trisphosphate 3-kinase C | Unknown | kinase |
| 3 | up | ILMN_68011 | JAK2 | Janus kinase 2 (a protein tyrosine kinase) | Cytoplasm | kinase |
| 3 | up | ILMN_69136 | KCNJ8 | potassium inwardly-rectifying channel, subfamily J, member 8 | Plasma Membrane | ion channel |
| 3 | up | ILMN_65986 | KCTD13 | potassium channel tetramerisation domain containing 13 | Cytoplasm | ion channel |
| 3 | up | ILMN_51953 | KIAA0913 | KIAA0913 | Unknown | other |
| 3 | up | ILMN_66324 | KIAA1522 | KIAA1522 | Unknown | other |
| 3 | up | ILMN_68024 | KLF6 | Kruppel-like factor 6 | Nucleus | transcription regulator |
| 3 | up | ILMN_51410 | KLRK1 | killer cell lectin-like receptor subfamily K, member 1 | Plasma Membrane | transmembrane receptor |
| 3 | up | ILMN_55199 | LASS5 | LAG1 homolog, ceramide synthase 5 | Cytoplasm | transcription regulator |
| 3 | up | ILMN_61915 | LEMD2 | LEM domain containing 2 | Unknown | other |
| 3 | up | ILMN_61804 | LINCR | likely ortholog of mouse lung-inducible Neutralized-related C3HC4 RING domain protein | Unknown | other |
| 3 | up | ILMN_63657 | LITAF | lipopolysaccharide-induced TNF factor | Nucleus | transcription regulator |
| 3 | up | ILMN_57407 | LOC150223 | LOC150223 protein | Unknown | other |
| 3 | up | ILMN_57052 | LOC288526 | similar to DNA segment on chromosome X and Y (unique) 155 expressed sequence isoform 1 | Unknown | other |
| 3 | up | ILMN_51709 | LOC361750 | similar to eukaryotic translation elongation factor 1 beta 2 | Unknown | other |
| 3 | up | ILMN_55267 | LOC499828 | similar to copine VIII isoform 1 | Unknown | other |
| 3 | up | ILMN_65707 | LSM12 | LSM12 homolog (S. cerevisiae) | Unknown | other |
| 3 | up | ILMN_61943 | LTV1 | LTV1 homolog (S. cerevisiae) | Unknown | other |
| 3 | up | ILMN_59289 | MAD2L2 | MAD2 mitotic arrest deficient-like 2 (yeast) | Nucleus | enzyme |
| 3 | up | ILMN_60991 | MAFF | v-maf musculoaponeurotic fibrosarcoma oncogene homolog F (avian) | Nucleus | transcription regulator |
| 3 | up | ILMN_49960 | MAP1S | microtubule-associated protein 1S | Cytoplasm | enzyme |
| 3 | up | ILMN_57859 | MAPK8IP3 | mitogen-activated protein kinase 8 interacting protein 3 | Cytoplasm | other |
| 3 | up | ILMN_57148 | MAPKAPK2 | mitogen-activated protein kinase-activated protein kinase 2 | Nucleus | kinase |
| 3 | up | ILMN_48224 | MARCH3 | membrane-associated ring finger (C3HC4) 3 | Cytoplasm | other |
| 3 | up | ILMN_160667 | MARVELD1 | MARVEL domain containing 1 | Unknown | other |
| 3 | up | ILMN_59352 | MARVELD1 | MARVEL domain containing 1 | Unknown | other |
| 3 | up | ILMN_50647 | MCL1 | myeloid cell leukemia sequence 1 (BCL2-related) | Cytoplasm | transporter |
| 3 | up | ILMN_63025 | MDFIC | MyoD family inhibitor domain containing | Nucleus | other |
| 3 | up | ILMN_56790 | MED10 | mediator complex subunit 10 | Unknown | other |
| 3 | up | ILMN_51054 | MED27 | mediator complex subunit 27 | Nucleus | transcription regulator |
| 3 | up | ILMN_53676 | METTL3 | methyltransferase like 3 | Nucleus | enzyme |
| 3 | up | ILMN_51516 | MINPP1 | multiple inositol polyphosphate histidine phosphatase, 1 | Cytoplasm | phosphatase |
| 3 | up | ILMN_52438 | MINPP1 | multiple inositol polyphosphate histidine phosphatase, 1 | Cytoplasm | phosphatase |
| 3 | up | ILMN_57806 | MMD | monocyte to macrophage differentiation-associated | Plasma Membrane | other |
| 3 | up | ILMN_58150 | MMRN2 | multimerin 2 | Extracellular Space | other |
| 3 | up | ILMN_64275 | MTF1 | metal-regulatory transcription factor 1 | Nucleus | transcription regulator |
| 3 | up | ILMN_53680 | MTMR7 | myotubularin related protein 7 | Cytoplasm | phosphatase |
| 3 | up | ILMN_55733 | MYC | v-myc myelocytomatosis viral oncogene homolog (avian) | Nucleus | transcription regulator |
| 3 | up | ILMN_69812 | N4BP1 | Nedd4 binding protein 1 | Cytoplasm | other |
| 3 | up | ILMN_61211 | NFKB2 | nuclear factor of kappa light polypeptide gene enhancer in B-cells 2 (p49/p100) | Nucleus | transcription regulator |
| 3 | up | ILMN_69984 | NFKBIB | nuclear factor of kappa light polypeptide gene enhancer in B-cells inhibitor, beta | Nucleus | transcription regulator |
| 3 | up | ILMN_66446 | NGDN | neuroguidin, EIF4E binding protein | Nucleus | other |
| 3 | up | ILMN_62687 | NNMT | nicotinamide N-methyltransferase | Cytoplasm | enzyme |
| 3 | up | ILMN_58904 | NOB1 | NIN1/RPN12 binding protein 1 homolog (S. cerevisiae) | Nucleus | other |
| 3 | up | ILMN_58852 | NR4A2 | nuclear receptor subfamily 4, group A, member 2 | Nucleus | ligand-dependent nuclear receptor |
| 3 | up | ILMN_57893 | NUPL1 | nucleoporin like 1 | Nucleus | transporter |
| 3 | up | ILMN_52937 | NUPR1 | nuclear protein 1 | Nucleus | transcription regulator |
| 3 | up | ILMN_63115 | NXT1 | NTF2-like export factor 1 | Nucleus | transporter |
| 3 | up | ILMN_52469 | OGFRL1 | opioid growth factor receptor-like 1 | Unknown | other |
| 3 | up | ILMN_54067 | OSTF1 | osteoclast stimulating factor 1 | Nucleus | transcription regulator |
| 3 | up | ILMN_50336 | P4HA1 | procollagen-proline, 2-oxoglutarate 4-dioxygenase (proline 4-hydroxylase), alpha polypeptide I | Cytoplasm | enzyme |
| 3 | up | ILMN_69877 | P4HA2 | procollagen-proline, 2-oxoglutarate 4-dioxygenase (proline 4-hydroxylase), alpha polypeptide II | Cytoplasm | enzyme |
| 3 | up | ILMN_64346 | PATL1 | protein associated with topoisomerase II homolog 1 (yeast) | Unknown | other |
| 3 | up | ILMN_58021 | PBX3 | pre-B-cell leukemia homeobox 3 | Nucleus | transcription regulator |
| 3 | up | ILMN_69675 | PCDH18 | protocadherin 18 | Extracellular Space | other |
| 3 | up | ILMN_52017 | PCGF1 | polycomb group ring finger 1 | Unknown | other |
| 3 | up | ILMN_67180 | PCK2 | phosphoenolpyruvate carboxykinase 2 (mitochondrial) | Cytoplasm | kinase |
| 3 | up | ILMN_69986 | PCSK6 | proprotein convertase subtilisin/kexin type 6 | Extracellular Space | peptidase |
| 3 | up | ILMN_64433 | PDCD11 | programmed cell death 11 | Nucleus | other |
| 3 | up | ILMN_54860 | PDE4B | phosphodiesterase 4B, cAMP-specific (phosphodiesterase E4 dunce homolog, Drosophila) | Cytoplasm | enzyme |
| 3 | up | ILMN_65328 | PHC2 | polyhomeotic homolog 2 (Drosophila) | Nucleus | other |
| 3 | up | ILMN_66452 | PHLDA1 | pleckstrin homology-like domain, family A, member 1 | Cytoplasm | other |
| 3 | up | ILMN_60599 | PIK3R1 | phosphoinositide-3-kinase, regulatory subunit 1 (p85 alpha) | Cytoplasm | kinase |
| 3 | up | ILMN_66297 | PLOD3 | procollagen-lysine, 2-oxoglutarate 5-dioxygenase 3 | Cytoplasm | enzyme |
| 3 | up | ILMN_54048 | PLSCR1 | phospholipid scramblase 1 | Plasma Membrane | enzyme |
| 3 | up | ILMN_67883 | POFUT2 | protein O-fucosyltransferase 2 | Cytoplasm | enzyme |
| 3 | up | ILMN_56601 | POLR3D | polymerase (RNA) III (DNA directed) polypeptide D, 44kDa | Nucleus | transcription regulator |
| 3 | up | ILMN_59305 | POP7 | processing of precursor 7, ribonuclease P/MRP subunit (S. cerevisiae) | Nucleus | enzyme |
| 3 | up | ILMN_60376 | PPM1G | protein phosphatase 1G (formerly 2C), magnesium-dependent, gamma isoform | Nucleus | phosphatase |
| 3 | up | ILMN_67094 | PPP2CB | protein phosphatase 2 (formerly 2A), catalytic subunit, beta isoform | Cytoplasm | phosphatase |
| 3 | up | ILMN_52415 | PPP2R2D | protein phosphatase 2, regulatory subunit B, delta isoform | Nucleus | phosphatase |
| 3 | up | ILMN_64580 | PPP3R1 | protein phosphatase 3 (formerly 2B), regulatory subunit B, alpha isoform | Cytoplasm | phosphatase |
| 3 | up | ILMN_62843 | PPRC1 | peroxisome proliferator-activated receptor gamma, coactivator-related 1 | Extracellular Space | other |
| 3 | up | ILMN_53716 | PRKCH | protein kinase C, eta | Cytoplasm | kinase |
| 3 | up | ILMN_56690 | PRKCZ | protein kinase C, zeta | Cytoplasm | kinase |
| 3 | up | ILMN_69681 | PROCR | protein C receptor, endothelial (EPCR) | Plasma Membrane | other |
| 3 | up | ILMN_62102 | PSCD2 | pleckstrin homology, Sec7 and coiled-coil domains 2 (cytohesin-2) | Cytoplasm | other |
| 3 | up | ILMN_53409 | PSMD2 | proteasome (prosome, macropain) 26S subunit, non-ATPase, 2 | Cytoplasm | other |
| 3 | up | ILMN_61490 | PTGS2 | prostaglandin-endoperoxide synthase 2 (prostaglandin G/H synthase and cyclooxygenase) | Cytoplasm | enzyme |
| 3 | up | ILMN_70340 | PTPN1 | protein tyrosine phosphatase, non-receptor type 1 | Cytoplasm | phosphatase |
| 3 | up | ILMN_51386 | PVR | poliovirus receptor | Plasma Membrane | G-protein coupled receptor |
| 3 | up | ILMN_69626 | RAB24 | RAB24, member RAS oncogene family | Cytoplasm | enzyme |
| 3 | up | ILMN_55792 | RALB | v-ral simian leukemia viral oncogene homolog B (ras related; GTP binding protein) | Cytoplasm | enzyme |
| 3 | up | ILMN_56547 | RAMP2 | receptor (G protein-coupled) activity modifying protein 2 | Plasma Membrane | other |
| 3 | up | ILMN_55389 | RANBP1 | RAN binding protein 1 | Nucleus | other |
| 3 | up | ILMN_64159 | RASIP1 | Ras interacting protein 1 | Cytoplasm | other |
| 3 | up | ILMN_63703 | RASL11B | RAS-like, family 11, member B | Unknown | enzyme |
| 3 | up | ILMN_56915 | RASSF5 | Ras association (RalGDS/AF-6) domain family 5 | Plasma Membrane | other |
| 3 | up | ILMN_54791 | RBM4 | RNA binding motif protein 4 | Nucleus | other |
| 3 | up | ILMN_49161 | REG3G (includes EG:130120) | regenerating islet-derived 3 gamma | Extracellular Space | growth factor |
| 3 | up | ILMN_62516 | REL | v-rel reticuloendotheliosis viral oncogene homolog (avian) | Nucleus | transcription regulator |
| 3 | up | ILMN_70090 | RELA | v-rel reticuloendotheliosis viral oncogene homolog A, nuclear factor of kappa light polypeptide gene enhancer in B-cells 3, p65 (avian) | Nucleus | transcription regulator |
| 3 | up | ILMN_54899 | RGD1559929 PREDICTED | similar to methylenetetrahydrofolate dehydrogenase (NAD) (EC 1.5.1.15)/methenyltetrahydrofolate cyclohydrolase (EC 3.5.4.9) precursor (predicted) | Unknown | other |
| 3 | up | ILMN_49623 | RGD1560523 PREDICTED | similar to S-adenosylmethionine synthetase gamma form (Methionine adenosyltransferase) (predicted) | Unknown | other |
| 3 | up | ILMN_53681 | RGD1561238 PREDICTED | similar to ring finger protein 122 homolog (predicted) | Unknown | other |
| 3 | up | ILMN_63947 | RGD1561628 PREDICTED | similar to Chain A, T13s Mutant Of Bovine 70 Kilodalton Heat Shock Protein (predicted) | Unknown | other |
| 3 | up | ILMN_62771 | RGD1562274 PREDICTED | RGD1562274 (predicted) | Unknown | other |
| 3 | up | ILMN_63436 | RGD1562543 | similar to ribosomal protein L27a (predicted) | Unknown | other |
| 3 | up | ILMN_64872 | RGD1562884 | similar to CCTeta, eta subunit of the chaperonin containing TCPdown (CCT) (predicted) | Unknown | other |
| 3 | up | ILMN_51662 | RGD1563689 PREDICTED | similar to KIAA0853 protein (predicted) | Unknown | other |
| 3 | up | ILMN_67849 | RGD1564040 PREDICTED | similar to methylenetetrahydrofolate dehydrogenase (NAD) (EC 1.5.1.15)/methenyltetrahydrofolate cyclohydrolase (EC 3.5.4.9) precursor (predicted) | Unknown | enzyme |
| 3 | up | ILMN_58486 | RGD1564392 | similar to Ig heavy chain V region PJ14 precursor | Unknown | other |
| 3 | up | ILMN_70156 | RGD1564577 PREDICTED | similar to Der1-like domain family, member 3 (predicted) | Unknown | other |
| 3 | up | ILMN_66021 | RGD1566091 | similar to nidogen 2 (predicted) | Unknown | other |
| 3 | up | ILMN_53056 | RGD1566092 PREDICTED | RGD1566092 (predicted) | Unknown | other |
| 3 | up | ILMN_67128 | RGD1566340 PREDICTED | similar to CDNA sequence BC029103 (predicted) | Unknown | other |
| 3 | up | ILMN_48055 | RGS2 | regulator of G-protein signaling 2, 24kDa | Nucleus | other |
| 3 | up | ILMN_52932 | RGS4 | regulator of G-protein signaling 4 | Cytoplasm | other |
| 3 | up | ILMN_59536 | RLF | rearranged L-myc fusion | Nucleus | transcription regulator |
| 3 | up | ILMN_53140 | RND1 | Rho family GTPase 1 | Cytoplasm | enzyme |
| 3 | up | ILMN_65170 | RNF145 | ring finger protein 145 | Unknown | other |
| 3 | up | ILMN_70005 | RNF217 | ring finger protein 217 | Unknown | enzyme |
| 3 | up | ILMN_67490 | RPIA | ribose 5-phosphate isomerase A (ribose 5-phosphate epimerase) | Cytoplasm | enzyme |
| 3 | up | ILMN_67014 | RRP15 | ribosomal RNA processing 15 homolog (S. cerevisiae) | Nucleus | other |
| 3 | up | ILMN_69788 | RRP9 | RRP9, small subunit (SSU) processome component, homolog (yeast) | Nucleus | other |
| 3 | up | ILMN_61659 | RUNX1 | runt-related transcription factor 1 (acute myeloid leukemia 1; aml1 oncogene) | Nucleus | transcription regulator |
| 3 | up | ILMN_67952 | RYK | RYK receptor-like tyrosine kinase | Plasma Membrane | kinase |
| 3 | up | ILMN_58286 | SAC3D1 | SAC3 domain containing 1 | Nucleus | other |
| 3 | up | ILMN_68181 | SAMD4B | sterile alpha motif domain containing 4B | Unknown | other |
| 3 | up | ILMN_57861 | SARM1 (includes EG:23098) | sterile alpha and TIR motif containing 1 | Plasma Membrane | transmembrane receptor |
| 3 | up | ILMN_54362 | SAV1 | salvador homolog 1 (Drosophila) | Unknown | other |
| 3 | up | ILMN_58309 | SBNO2 | strawberry notch homolog 2 (Drosophila) | Unknown | other |
| 3 | up | ILMN_59002 | SCAMP1 | secretory carrier membrane protein 1 | Cytoplasm | transporter |
| 3 | up | ILMN_48587 | SCARB2 | scavenger receptor class B, member 2 | Plasma Membrane | other |
| 3 | up | ILMN_49378 | SDCBP | syndecan binding protein (syntenin) | Plasma Membrane | enzyme |
| 3 | up | ILMN_70252 | SELP | selectin P (granule membrane protein 140kDa, antigen CD62) | Plasma Membrane | other |
| 3 | up | ILMN_62842 | SERPINA11 | serpin peptidase inhibitor, clade A (alphadown antiproteinase, antitrypsin), member 11 | Extracellular Space | other |
| 3 | up | ILMN_64117 | SERPINE1 | serpin peptidase inhibitor, clade E (nexin, plasminogen activator inhibitor type 1), member 1 | Extracellular Space | other |
| 3 | up | ILMN_64117 | SERPINE1 | serpin peptidase inhibitor, clade E (nexin, plasminogen activator inhibitor type 1), member 1 | Extracellular Space | other |
| 3 | up | ILMN_64626 | SFPQ | splicing factor proline/glutamine-rich (polypyrimidine tract binding protein associated) | Nucleus | other |
| 3 | up | ILMN_63191 | SFT2D1 | SFT2 domain containing 1 | Unknown | other |
| 3 | up | ILMN_58365 | SH2B2 | SH2B adaptor protein 2 | Cytoplasm | other |
| 3 | up | ILMN_50677 | SH3BP4 | SH3-domain binding protein 4 | Unknown | other |
| 3 | up | ILMN_60038 | SKAP2 | src kinase associated phosphoprotein 2 | Cytoplasm | other |
| 3 | up | ILMN_53873 | SLC10A7 | solute carrier family 10 (sodium/bile acid cotransporter family), member 7 | Unknown | transporter |
| 3 | up | ILMN_64676 | SLC16A3 | solute carrier family 16, member 3 (monocarboxylic acid transporter 4) | Plasma Membrane | transporter |
| 3 | up | ILMN_47895 | SLC1A4 | solute carrier family 1 (glutamate/neutral amino acid transporter), member 4 | Plasma Membrane | transporter |
| 3 | up | ILMN_53534 | SLC20A1 | solute carrier family 20 (phosphate transporter), member 1 | Plasma Membrane | transporter |
| 3 | up | ILMN_47981 | SLC38A2 | solute carrier family 38, member 2 | Plasma Membrane | transporter |
| 3 | up | ILMN_52984 | SLC39A10 (includes EG:57181) | solute carrier family 39 (zinc transporter), member 10 | Unknown | transporter |
| 3 | up | ILMN_69749 | SLC3A2 | solute carrier family 3 (activators of dibasic and neutral amino acid transport), member 2 | Plasma Membrane | transporter |
| 3 | up | ILMN_65160 | SLC41A2 | solute carrier family 41, member 2 | Unknown | transporter |
| 3 | up | ILMN_58339 | SLC5A3 | solute carrier family 5 (inositol transporters), member 3 | Plasma Membrane | transporter |
| 3 | up | ILMN_51318 | SLC9A3R1 | solute carrier family 9 (sodium/hydrogen exchanger), member 3 regulator 1 | Plasma Membrane | other |
| 3 | up | ILMN_51608 | SOCS3 | suppressor of cytokine signaling 3 | Cytoplasm | other |
| 3 | up | ILMN_49409 | SOD2 | superoxide dismutase 2, mitochondrial | Cytoplasm | enzyme |
| 3 | up | ILMN_53299 | SOX17 | SRY (sex determining region Y)-box 17 | Nucleus | transcription regulator |
| 3 | up | ILMN_50745 | SRM | spermidine synthase | Unknown | enzyme |
| 3 | up | ILMN_63194 | SSR2 | signal sequence receptor, beta (translocon-associated protein beta) | Cytoplasm | other |
| 3 | up | ILMN_57996 | ST3GAL4 | ST3 beta-galactoside alpha-2,3-sialyltransferase 4 | Cytoplasm | enzyme |
| 3 | up | ILMN_65443 | STARD13 | StAR-related lipid transfer (START) domain containing 13 | Cytoplasm | other |
| 3 | up | ILMN_50314 | STIP1 | stress-induced-phosphoprotein 1 (Hsp70/Hsp90-organizing protein) | Cytoplasm | other |
| 3 | up | ILMN_52472 | STK11IP | serine/threonine kinase 11 interacting protein | Unknown | other |
| 3 | up | ILMN_55921 | STK35 | serine/threonine kinase 35 | Cytoplasm | kinase |
| 3 | up | ILMN_59880 | STOM | stomatin | Plasma Membrane | other |
| 3 | up | ILMN_59743 | STS | steroid sulfatase (microsomal), isozyme S | Cytoplasm | enzyme |
| 3 | up | ILMN_63287 | TES | testis derived transcript (3 LIM domains) | Plasma Membrane | other |
| 3 | up | ILMN_50620 | TEX10 | testis expressed 10 | Nucleus | other |
| 3 | up | ILMN_58272 | TFPI2 | tissue factor pathway inhibitor 2 | Extracellular Space | other |
| 3 | up | ILMN_61579 | TGM1 | transglutaminase 1 (K polypeptide epidermal type I, protein-glutamine-gamma-glutamyltransferase) | Plasma Membrane | enzyme |
| 3 | up | ILMN_52754 | TGM2 | transglutaminase 2 (C polypeptide, protein-glutamine-gamma-glutamyltransferase) | Cytoplasm | enzyme |
| 3 | up | ILMN_64907 | THG1L | tRNA-histidine guanylyltransferase 1-like (S. cerevisiae) | Cytoplasm | enzyme |
| 3 | up | ILMN_69764 | TIFA | TRAF-interacting protein with a forkhead-associated domain | Unknown | other |
| 3 | up | ILMN_62559 | TIMP1 | TIMP metallopeptidase inhibitor 1 | Extracellular Space | other |
| 3 | up | ILMN_69164 | TLN1 | talin 1 | Plasma Membrane | other |
| 3 | up | ILMN_63064 | TLR6 | toll-like receptor 6 | Plasma Membrane | transmembrane receptor |
| 3 | up | ILMN_53153 | TMEM185A | transmembrane protein 185A | Unknown | other |
| 3 | up | ILMN_69826 | TNFRSF12A | tumor necrosis factor receptor superfamily, member 12A | Plasma Membrane | other |
| 3 | up | ILMN_50114 | TNFRSF21 | tumor necrosis factor receptor superfamily, member 21 | Plasma Membrane | other |
| 3 | up | ILMN_65713 | TNIP2 | TNFAIP3 interacting protein 2 | Cytoplasm | other |
| 3 | up | ILMN_62392 | TNPO1 | transportin 1 | Nucleus | transporter |
| 3 | up | ILMN_61853 | TOMM40 | translocase of outer mitochondrial membrane 40 homolog (yeast) | Cytoplasm | ion channel |
| 3 | up | ILMN_64448 | TOPORS | topoisomerase I binding, arginine/serine-rich | Nucleus | enzyme |
| 3 | up | ILMN_49056 | TPR | translocated promoter region (to activated MET oncogene) | Nucleus | other |
| 3 | up | ILMN_69411 | TRAF2 | TNF receptor-associated factor 2 | Cytoplasm | other |
| 3 | up | ILMN_69704 | TRAF4 | TNF receptor-associated factor 4 | Cytoplasm | other |
| 3 | up | ILMN_52379 | TRAPPC4 | trafficking protein particle complex 4 | Cytoplasm | other |
| 3 | up | ILMN_48384 | TRIB1 | tribbles homolog 1 (Drosophila) | Cytoplasm | kinase |
| 3 | up | ILMN_63345 | TRIB3 | tribbles homolog 3 (Drosophila) | Nucleus | kinase |
| 3 | up | ILMN_56454 | TRIM3 | tripartite motif-containing 3 | Cytoplasm | other |
| 3 | up | ILMN_58628 | TWF1 | twinfilin, actin-binding protein, homolog 1 (Drosophila) | Cytoplasm | kinase |
| 3 | up | ILMN_50100 | TXNDC12 | thioredoxin domain containing 12 (endoplasmic reticulum) | Cytoplasm | enzyme |
| 3 | up | ILMN_54549 | UBAC1 | UBA domain containing 1 | Unknown | other |
| 3 | up | ILMN_51000 | UBE2J2 | ubiquitin-conjugating enzyme E2, J2 (UBC6 homolog, yeast) | Cytoplasm | enzyme |
| 3 | up | ILMN_48354 | UBE2S | ubiquitin-conjugating enzyme E2S | Unknown | enzyme |
| 3 | up | ILMN_53370 | UBFD1 | ubiquitin family domain containing 1 | Unknown | other |
| 3 | up | ILMN_49772 | UGCG | UDP-glucose ceramide glucosyltransferase | Cytoplasm | enzyme |
| 3 | up | ILMN_57568 | VCAN | versican | Extracellular Space | other |
| 3 | up | ILMN_49915 | VEZT | vezatin, adherens junctions transmembrane protein | Plasma Membrane | other |
| 3 | up | ILMN_64761 | VHL | von Hippel-Lindau tumor suppressor | Nucleus | other |
| 3 | up | ILMN_53654 | WARS | tryptophanyl-tRNA synthetase | Cytoplasm | enzyme |
| 3 | up | ILMN_55207 | WDR81 | WD repeat domain 81 | Unknown | other |
| 3 | up | ILMN_60302 | XBP1 | X-box binding protein 1 | Nucleus | transcription regulator |
| 3 | up | ILMN_49956 | YKT6 | YKT6 v-SNARE homolog (S. cerevisiae) | Cytoplasm | enzyme |
| 3 | up | ILMN_61050 | YTHDC1 | YTH domain containing 1 | Cytoplasm | other |
| 3 | up | ILMN_69696 | ZBED4 | zinc finger, BED-type containing 4 | Nucleus | other |
| 3 | up | ILMN_48245 | ZFAND2A | zinc finger, AN1-type domain 2A | Unknown | other |
| 3 | up | ILMN_51927 | ZFP36L2 | zinc finger protein 36, C3H type-like 2 | Nucleus | transcription regulator |
| 3 | up | ILMN_48923 | ZFYVE27 | zinc finger, FYVE domain containing 27 | Unknown | other |
| 3 | up | ILMN_65658 | ZMYND19 | zinc finger, MYND-type containing 19 | Plasma Membrane | other |
| 3 | up | ILMN_58745 | ZNF335 | zinc finger protein 335 | Nucleus | other |
| 3 | up | ILMN_56960 | ZNF598 | zinc finger protein 598 | Unknown | other |
| 3 | up | ILMN_55430 | ZRF1 | zuotin related factor 1 | Nucleus | other |
